# Tumor cell-specific loss of GPX4 reprograms triacylglycerol metabolism to escape ferroptosis and impair antitumor immunity in non-small cell lung cancer

**DOI:** 10.1093/procel/pwaf101

**Published:** 2025-11-19

**Authors:** Peng Wang, Shengdan Zhang, Xin Chen, Xu-Dong Yang, Shi Huang, Huiyong Yin, Hao-Yu Duan, Fuling Zhou, Jia Yu, Bo Zhong, Dandan Lin

**Affiliations:** Department of Gastrointestinal Surgery, Zhongnan Hospital of Wuhan University, Cancer Center, Renmin Hospital of Wuhan University, Medical Research Institute, Frontier Science Center of Immunology and Metabolism, College of Life Sciences, State Key Laboratory of Metabolism and Regulation in Complex Organisms, State Key Laboratory of Virology and Biosafety, Hubei Provincial Research Center for Basic Biological Sciences, Wuhan University, Wuhan 430071, China; Research Center for Lifespan Health, School of Nursing, Department of Hematology, Zhongnan Hospital of Wuhan University, Wuhan University, Wuhan 430071, China; Department of Stomatology, Zhongnan Hospital of Wuhan University, Wuhan 430071, China; AS Key Laboratory of Nutrition, Metabolism and Food Safety, Shanghai Institute of Nutrition and Health, University of Chinese Academy of Sciences, Chinese Academy of Sciences, Shanghai 200031, China; Department of Gastrointestinal Surgery, Zhongnan Hospital of Wuhan University, Cancer Center, Renmin Hospital of Wuhan University, Medical Research Institute, Frontier Science Center of Immunology and Metabolism, College of Life Sciences, State Key Laboratory of Metabolism and Regulation in Complex Organisms, State Key Laboratory of Virology and Biosafety, Hubei Provincial Research Center for Basic Biological Sciences, Wuhan University, Wuhan 430071, China; Department of Gastrointestinal Surgery, Zhongnan Hospital of Wuhan University, Cancer Center, Renmin Hospital of Wuhan University, Medical Research Institute, Frontier Science Center of Immunology and Metabolism, College of Life Sciences, State Key Laboratory of Metabolism and Regulation in Complex Organisms, State Key Laboratory of Virology and Biosafety, Hubei Provincial Research Center for Basic Biological Sciences, Wuhan University, Wuhan 430071, China; AS Key Laboratory of Nutrition, Metabolism and Food Safety, Shanghai Institute of Nutrition and Health, University of Chinese Academy of Sciences, Chinese Academy of Sciences, Shanghai 200031, China; Department of Gastrointestinal Surgery, Zhongnan Hospital of Wuhan University, Cancer Center, Renmin Hospital of Wuhan University, Medical Research Institute, Frontier Science Center of Immunology and Metabolism, College of Life Sciences, State Key Laboratory of Metabolism and Regulation in Complex Organisms, State Key Laboratory of Virology and Biosafety, Hubei Provincial Research Center for Basic Biological Sciences, Wuhan University, Wuhan 430071, China; Research Center for Lifespan Health, School of Nursing, Department of Hematology, Zhongnan Hospital of Wuhan University, Wuhan University, Wuhan 430071, China; Department of Hepatobiliary Surgery, Renmin Hospital of Wuhan University, Wuhan 430060, China; Department of Gastrointestinal Surgery, Zhongnan Hospital of Wuhan University, Cancer Center, Renmin Hospital of Wuhan University, Medical Research Institute, Frontier Science Center of Immunology and Metabolism, College of Life Sciences, State Key Laboratory of Metabolism and Regulation in Complex Organisms, State Key Laboratory of Virology and Biosafety, Hubei Provincial Research Center for Basic Biological Sciences, Wuhan University, Wuhan 430071, China; TaiKang Center for Life and Medical Sciences, Wuhan University, Wuhan 430071, China; Department of Gastrointestinal Surgery, Zhongnan Hospital of Wuhan University, Cancer Center, Renmin Hospital of Wuhan University, Medical Research Institute, Frontier Science Center of Immunology and Metabolism, College of Life Sciences, State Key Laboratory of Metabolism and Regulation in Complex Organisms, State Key Laboratory of Virology and Biosafety, Hubei Provincial Research Center for Basic Biological Sciences, Wuhan University, Wuhan 430071, China

**Keywords:** GPX4, triacylglycerol, lipid droplets, lipid release, non-small cell lung cancer, tumor microenvironment

## Abstract

Glutathione peroxidase 4 (GPX4) is a master regulator of ferroptosis, a process that has been proposed as a potential therapeutic strategy for cancer. Here, we have unexpectedly found that inducible knockout of GPX4 in tumor cells significantly promotes non-small cell lung cancer (NSCLC) progression in the autochthonous *Kras*^LSL-G12D/+^*Lkb1*^fl/fl^ (KL) and *Kras*^LSL-G12D/+^*Tp53*^fl/fl^ (KP) mouse models, whereas inducible overexpression of GPX4 in tumor cells exerts the opposite effect. GPX4-deficient tumor cells evade ferroptosis by upregulating the expression of DGAT1/2 to promote the synthesis of triacylglycerol (TAG) and oxidized TAG (oxTAG) and the formation of lipid droplets in cells. In addition, GPX4-deficient tumor cells secrete TAG and oxTAG into the extracellular space to induce dysfunction of antitumor CD8^+^ T cells, thereby coordinating an immunoinhibitory tumor microenvironment (TME). Consistently, treatment with DGAT1/2 inhibitors or inducible overexpression of GPX4 in tumor cells significantly resensitizes tumor cells to ferroptosis and ignites the activation of T cells in the TME to inhibit NSCLC progression. These findings highlight a previously uncharacterized role of tumor cell-specific GPX4 in NSCLC progression and provide potential therapeutic strategies for NSCLC.

## Introduction

Glutathione peroxidase 4 (GPX4) is an enzyme that catalyzes the reduction of cytotoxic phospholipid hydroperoxides (PL-OOH) into PL alcohols (PL-OH) and thereby protects against ferroptosis (Cozza et al., 2017; Ursini et al., 1982). Deletion of GPX4 leads to the accumulation of PL peroxides and induces cell death in *in vitro* cultured cells (Canli et al., 2016; Hambright et al., 2017; Matsushita et al., 2015; Muri et al., 2019; Seiler et al., 2008; Xu et al., 2021a; Yao et al., 2021). Accordingly, *Gpx4*^−/−^ mouse embryos die at E7.5 and Cre-ER; *Gpx4*^fl/fl^ mice exhibit lethality after 2 weeks of tamoxifen (Tam) treatment (Friedmann Angeli et al., 2014; Seiler et al., 2008; Yant et al., 2003; Yoo et al., 2012), whereas the human *GPX4* transgene rescues the lethal phenotype of *Gpx4*^−/−^ mice and inhibits PL peroxide-induced cell death (Ran et al., 2004), demonstrating an indispensable role of GPX4 in the survival and development of mice under homeostatic conditions. In RAS^mut^ cancer cells, genetic deletion or inhibition of GPX4 induces PL peroxidation and ferroptosis and inhibits tumor cell growth in mice (Dixon et al., 2012; Yang et al., 2014), suggesting a protumor role of GPX4 in xenograft or syngeneic graft models. However, conditional deletion of GPX4 in the pancreas (*Pdx1*-Cre; *Gpx4*^fl/fl^) of mice promotes pancreatic tumorigenesis in the KRas^G12D^ model (Dai et al., 2020), arguing a protective role of GPX4 in the spontaneous RAS^mut^ cancer model. It should be noted that such a strategy of GPX4 depletion might cause cell death or tissue injury at the early stage of cancer development or even before the malignant transformation of tumor cells, which might have an impact on the functions of GPX4 in tumor progression *in vivo*.

The tumor microenvironment (TME), which includes immune and non-immune cells, metabolites, and cytokines, critically regulates the progression and drug response of cancers (Herbst et al., 2018; Tang et al., 2023; You et al., 2023). Lung cancer is the leading cause of cancer-related mortality (Siegel et al., 2023; Wang et al., 2023), and approximately 85% of the diagnosed lung cancers are non-small cell lung cancer (NSCLC) (Herbst et al., 2018). Various mutations, including those in the tumor-driving genes *KRAS*, *EGFR*, and *ALK* and the tumor suppressor genes *TP53* and *LKB1*, have been identified in NSCLC (Chen et al., 2020; Gillette et al., 2020; Skoulidis and Heymach, 2019; Xu et al., 2020). Studies with genetically engineered mouse models (GEMMs) harboring the mutations (e.g., *Kras*^LSL-G12D/+^*Tp53*^fl/fl^, KP; *Kras*^LSL-G12D/+^*Lkb1*^fl/fl^, KL) have been conducted to elucidate the mechanisms of and screen effective therapies for NSCLC (Chen et al., 2012; Ji et al., 2007; Johnson et al., 2001). For example, we have found that the chemokine CCL7 secreted by alveolar macrophages in the TME recruits type 1 conventional dendritic cells to promote an antitumor T-cell response and inhibit NSCLC progression (Dong et al., 2023; Zhang et al., 2020), whereas neutrophils in the TME produce IL-36γ to alleviate the oxidative stress and promote NSCLC progression by increasing glutathione metabolism in the KL and KP models (Wang et al., 2021). In addition, antioxidants such as N-acetylcysteine and vitamin E accelerate KRas^G12D^-driven NSCLC progression by tuning down the global oxidative stress in the TME (Sayin et al., 2014). Considering the essential role of GPX4 in the reduction of lipid peroxides that contribute to the oxidative stress of cancer cells, whether and how the regulation of lipid peroxidation by GPX4 in tumor cells modulates NSCLC progression in the autochthonous mouse models remain to be investigated.

Here, we have adopted a dual recombinase-mediated gene mutation system (Cre-loxP and DreER^T2^-rox) and a Cre recombinase plus doxycycline (Dox)-rTTA system to specifically and inducibly delete and overexpress GPX4 in tumor cells in the autochthonous KL and KP NSCLC mouse models, respectively. Interestingly, inducible knockout of GPX4 in the tumor cells of the established autochthonous KL or KP tumors significantly promotes the progression of NSCLC and accelerates the death of mice, and inducible overexpression of GPX4 in tumor cells has the opposite effect. Mechanistically, inducible knockout of GPX4 in tumor cells leads to the accumulation of triacylglycerol (TAG) and oxidized TAG (oxTAG) in tumor cells and the formation of lipid droplets in a DGAT1/2-dependent manner, thereby protecting tumor cells from ferroptosis. In addition, inducible knockout of GPX4 in tumor cells promotes the secretion of TAG and oxTAG, which induces dysfunction and exhaustion of antitumor CD8^+^ T cells in the TME. Selective overexpression of GPX4 in tumor cells or pharmacological inhibition of DGAT1/2 impairs the synthesis of TAG and oxTAG, promotes the generation of oxPE/PC in tumor cells, and enhances antitumor immunity to inhibit NSCLC progression in autochthonous mouse models. These findings have demonstrated an unexpected role of tumor cell-specific GPX4 in reprogramming TAG metabolism and antitumor immunity in the TME and the progression of autochthonous NSCLC.

## Results

### Inducible knockout of GPX4 in tumor cells promotes NSCLC progression in autochthonous KP and KL mouse models

To investigate the role of GPX4 in tumor cells in autochthonous KP and KL NSCLC models, we took advantage of the Cre-loxP and the DreER^T2^-rox systems to inducibly delete GPX4 in tumor cells of the established KP and KL tumors. Specifically, the targeting vector consisting of rox-*Gpx4*-rox-DreER^T2^-loxP2272-STOP-loxP2272-CAG promoter and the flanking homologous sequences was knocked into the *Gpx4* gene locus to generate *Gpx4*-rox-CAG-LSL-DreER^T2^ mice (termed *Gpx4*^m/m^ hereafter) (Fig. S1A and S1B). Cre-mediated removal of the STOP cassette allowed the expression of the DreER^T2^ protein that would induce the knockout of GPX4 only after 4-hydroxytamoxifen (4-OHT) treatment (Fig. S1C). We next intranasally infected the *Kras*^LSL-G12D/+^*Lkb1*^fl/fl^*Gpx4*^m/m^ (KLG4^m/m^) mice with Ad-Cre, and 5 weeks later (when KRas^G12D^ induced malignant transformation and when the tumor lesions were formed) (Dong et al., 2023; Ji et al., 2007; Tang et al., 2024; Wang et al., 2021), the mice were intraperitoneally injected with corn oil or tamoxifen (Tam) (which was metabolized into 4-OHT in mice) every other day for 2 weeks. The mice were then euthanized for various analyses (Fig. S2A). The autochthonous NSCLC models in this study were induced in this way otherwise specified. PCR analysis of genomic DNA from different tissues suggested that exons 5–7 of the *Gpx4* gene were specifically deleted in tumors but not in lung non-cancerous tumor-adjacent tissue (NAT), heart, liver, spleen, kidney, or brain or in the organs of corn oil-treated KLG4^m/m^ counterparts (Fig. S2B). We further FACS-sorted different types of cells in the KL and the KLG4^m/m^ tumors (Fig. S2C), and found that the rearrangement of the *Gpx4* gene selectively occurred in tumor cells (EpCAM^+^CD31^−^) (Fig. S2D). Additionally, the protein levels of GPX4 were substantially decreased in EpCAM^+^CD31^−^ tumor cells but not in endothelial cells (CD31^+^CD49d^−/int^), stromal cells (CD31^−^CD49d^+^), or immune cells (CD45^+^) in KLG4^m/m^ tumors compared to the KL counterparts (Fig. S2E), suggesting efficient, specific and inducible deletion of GPX4 in tumor cells in the KLG4^m/m^ autochthonous NSCLC mouse model.

**Figure 1.**
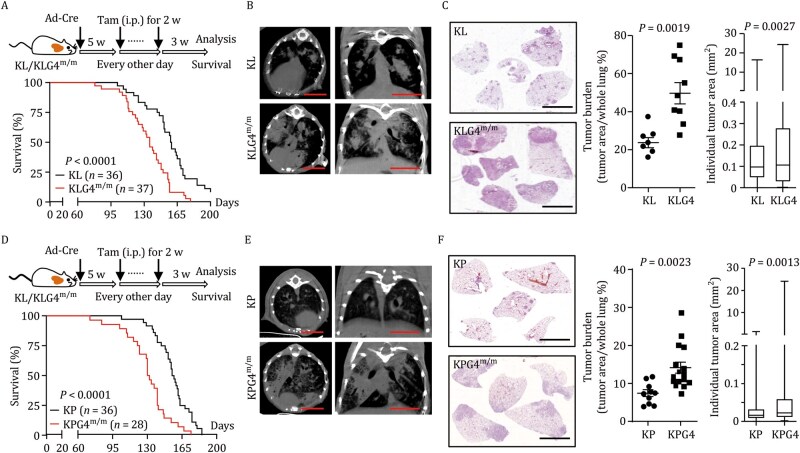
**Inducible knockout of GPX4 in tumor cells promotes NSCLC progression in the autochthonous KP and KL mouse models**. (A) A scheme of tumor induction (upper) and the survival (lower) of *Kras*^LSL-G12D/+^*Lkb1*^fl/fl^ (KL, *n *= 36) and *Kras*^LSL-G12D/+^*Lkb1*^fl/fl^*Gpx4*^m/m^ (KLG4^m/m^, *n *= 37) mice. The KL and the KLG4^m/m^ mice were intranasally injected with Ad-Cre (2 × 10^6^ PFU per mouse) for 5 weeks followed by intraperitoneal injection of tamoxifen every other day for 2 weeks. The mice were allowed to rest for another 3 weeks followed by various analyses or monitored for survival.(B, C) Representative images of micro-CT (B) and H&E staining (C, left) and statistics of tumor burdens (C, middle) and individual tumor sizes (C, right) of tumor-burdened lungs from the KL (*n *= 7) and KLG4^m/m^ (*n *= 9) mice treated as in (A). (D) A scheme of tumor induction (upper) and the survival (lower) of *Kras*^LSL-G12D/+^*Tp53*^fl/fl^ (KP, *n *= 36) and *Kras*^LSL-G12D/+^*Tp53*^fl/fl^*Gpx4*^m/m^ (KPG4^m/m^, *n *= 28) mice that were treated as described in (A). (E and F) Representative images of micro-CT (E) and H&E staining (F, left), and statistics of tumor burdens (F, middle) and individual tumor sizes (F, right) of tumor-burdened lungs from KP (*n *= 10) and KPG4^m/m^ (*n *= 16) mice treated as in (D). Statistical analyses were performed with log-rank analysis (A, D) or two-tailed Student’s *t*-test (C, F). Graphs show mean ± SEM (C, F). Scale bars represent 5 mm (B, C, E, F). Data are combined results of three independent experiments (A, D) or representative results of two independent experiments (B, C, E, F).

We next examined the effect of inducible knockout of GPX4 in tumor cells on the progression of autochthonous murine NSCLC. Interestingly, we found that the survival of KLG4^m/m^ mice was significantly shorter than that of KL mice after Ad-Cre infection and Tam treatment (Fig. 1A). In addition, the tumor burdens and the individual tumor sizes in the lungs of KLG4^m/m^ mice were significantly greater than those in the lungs of KL mice, as suggested by micro-computed tomography (micro-CT) imaging and hematoxylin and eosin (H&E) staining analyses (Fig. 1B and 1C). With similar treatments, the *Kras*^LSL-G12D/+^*Tp53*^fl/fl^*Gpx4*^m/m^ (KPG4^m/m^) mice exhibited shorter survival time, larger tumor sizes, and heavier tumor burdens than the KP mice (Fig. 1D–F). In addition, the KLG4^m/m^ mice had larger tumor sizes and heavier tumor burdens in the lungs than did the KL mice after intranasal injection of Ad-SPC-Cre, which has been shown to specifically and efficiently express the Cre recombinase in lung alveolar type II epithelial cells (Li et al., 2015; Perl et al., 2002; Sutherland et al., 2014), followed by Tam treatment (Fig. S3A and S3B). These data demonstrate that inducible knockout of GPX4 in tumor cells promotes autochthonous NSCLC progression in the KL and KP mouse models.

### Inducible knockout of GPX4 inhibits tumor growth in syngeneic graft mouse models

Previous studies have shown that knockdown or inhibition of GPX4 in RAS^mut^ tumor cell lines induces cancer cell ferroptosis and inhibits tumor growth (Dixon et al., 2012; Yang et al., 2014). We next examined the role of GPX4 in tumor cell growth in syngeneic graft models. The CD45^−^CD31^−^EpCAM^+^ tumor cells from KL and KLG4^m/m^ tumors were subcutaneously inoculated into the flanks of wild-type C57BL/6 mice followed by various analyses (Fig. S3C). The results suggested that GPX4 was efficiently deleted in the KLG4^m/m^ tumors and that the growth of KLG4^m/m^ tumor cells was severely compromised compared to the KL tumor cells (Fig. S3C and S3D), suggesting that GPX4 is indispensable for tumor cell growth in the subcutaneous syngeneic graft model.

To examine the role of GPX4 in maintaining tumor growth, we isolated the CD45^−^CD31^−^EpCAM^+^ tumor cells from KL and KLG4^m/m^ mice that were intranasally infected with Ad-Cre for 10 weeks and subcutaneously inoculated the cells into the flanks of wild-type C57BL/6 mice followed by Tam treatment (Fig. S3E). The results suggested that the growth of KLG4^m/m^ tumors was similar to that of KL tumors before Tam treatment, whereas the growth of KLG4^m/m^ tumors but not KL tumors was significantly inhibited after Tam treatment, which was accompanied by a decrease of GPX4 in KLG4^m/m^ tumors (Fig. S3E–G). These data demonstrate that GPX4 is required for tumor cell growth and maintenance in subcutaneous syngeneic graft models.

### Liprostatin-1 restores syngeneic KLG4^m/m^ tumor growth

Knockout or inhibition of GPX4 promotes lipid peroxidation and ferroptosis in *in vitro* cell cultures, which is rescued by lipophilic radical-trapping agents such as liprostatin-1 (Lip-1) or α-tocopherol (Friedmann Angeli et al., 2014; Seiler et al., 2008; Yang et al., 2014). Consistent with this notion, the tumor cells from Tam-treated syngeneic KLG4^m/m^ tumors exhibited higher lipid peroxidation and more severe ferroptotic death than did those from Tam-treated syngeneic KL tumors, as indicated by C11-BODIPY and SYTOX staining (Fig. S3H). Treatment with Lip-1 substantially decreased lipid peroxidation and ferroptotic cell death of KLG4^m/m^ tumor cells and restored the growth of KLG4^m/m^ tumors that should have shrunk after Tam treatment (Fig. S3E–H). These data suggest that inducible knockout of GPX4 results in ferroptosis of KL tumor cells in syngeneic subcutaneous models.

We next examined lipid peroxidation in the tumor cells of the autochthonous KL and KLG4^m/m^ tumors and found that the C11-BODIPY staining in CD45^−^CD31^−^EpCAM^+^ tumor cells from autochthonous KLG4^m/m^ tumors was significantly higher than in those from KL tumors (Fig. S4A). Interestingly, however, we did not observe increased death or mitochondrial damage in KLG4^m/m^ CD45^−^CD31^−^EpCAM^+^ tumor cells compared to the KL counterparts as revealed by the SYTOX staining and transmission electron microscopy (TEM) analyses (Fig. S4B and S4C). This phenomenon was further confirmed by immunofluorescence staining of 4-hydroxynonenal (4-HNE, a downstream metabolite of PL peroxidation, which is commonly used as a surrogate marker of ferroptosis), and terminal deoxynucleotidyl transferase (TdT)-mediated dUTP nick end labeling (TUNEL) within the EpCAM^+^ cells in KL and KLG4^m/m^ tumors (Fig. S4D). Lipidomic analysis revealed that the levels of classical ferroptosis-associated oxygenated phosphatidylcholine (PC) (PC 18:0/22:4[2O] and PC 18:0/20:4[2O]) and phosphatidylethanolamine (PE) (PE 18:0/20:4[2O] and PE 18:0a_HETE), polyunsaturated fatty acid (PUFA)-PC and PUFA-PE and free fatty acid (FFA) were comparable between the KL and the KLG4^m/m^ tumor cells (Doll et al., 2019; Friedmann Angeli et al., 2014; Kim et al., 2022; Li et al., 2024) (Fig. S4E–G; Table S1A), indicating that inducible GPX4 deficiency in tumor cells does not lead to the accumulation of ferroptotic PL hydroperoxides in the autochthonous NSCLC models.

### The autochthonous *Gpx4*-null tumor cells adapt to TAG synthesis and lipid droplet formation

**Figure 2.**
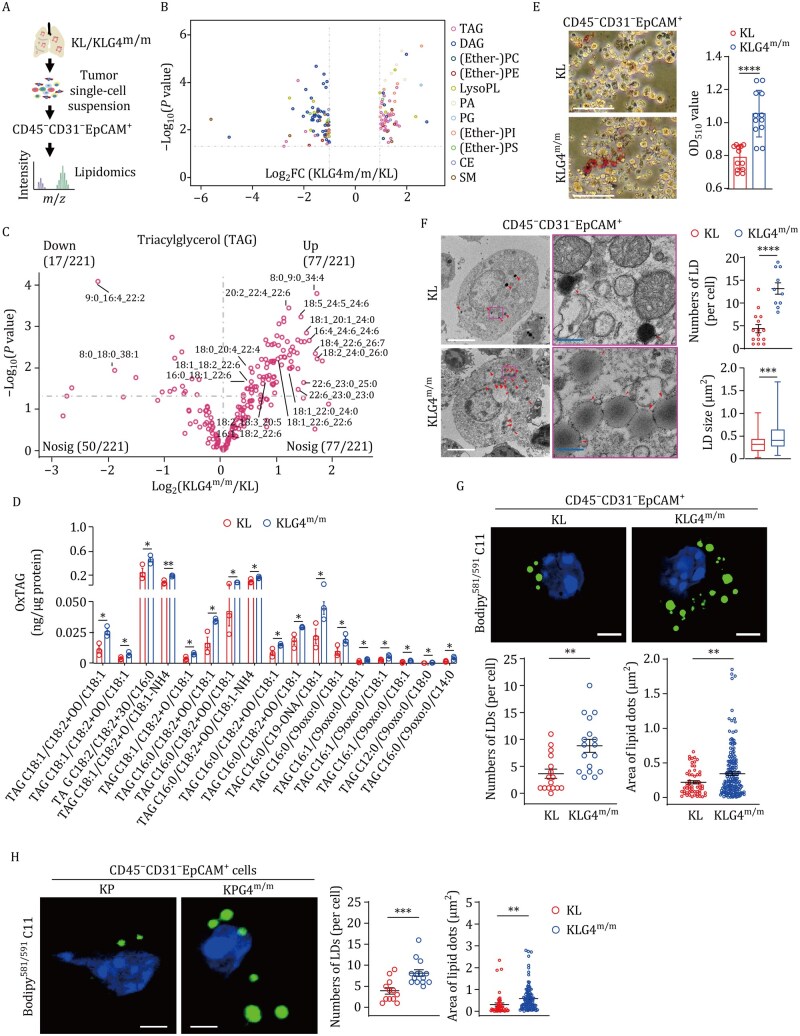
**Inducible knockout of GPX4 in tumor cells increases the TAG accumulation and the lipid droplet formation in the autochthonous NSCLC models**. (A) A workflow scheme of lipidomics analyses of KL and KLG4^m/m^ CD45^−^CD31^−^EpCAM^+^ tumor cells. (B) Volcano plot of differential lipids (*P* < 0.05 and |log_2_[fold change (FC)]| ≥ 1) in KLG4^m/m^ versus KL CD45^−^CD31^−^EpCAM^+^ tumor cells. (C) Volcano plot showing the levels of triacylglycerols (TAGs) in CD45^−^CD31^−^EpCAM^+^ tumor cells derived from KLG4^m/m^ mice (*n* = 3) versus KL-derived tumor cells (*n* = 3). (D) Quantitative assessment of oxidized triacylglycerols (oxTAGs) in CD45^−^CD31^−^EpCAM^+^ tumor cells from lung tumors of KL (*n* = 3) and KLG4^m/m^ (*n* = 3) mice. (E) Representative images (left) and statistical analysis (right) of oil red O staining of CD45^−^CD31^−^EpCAM^+^ tumor cells from KL (*n* = 12) and KLG4^m/m^ (*n* = 12) lung tumors. (F) Representative images from transmission electron microscopy (left, red arrowheads indicate lipid droplets) along with statistics of the numbers and sizes (right) of lipid droplets (LDs) in CD45^−^CD31^−^EpCAM^+^ tumor cells from KL (*n* = 15) and KLG4^m/m^ (*n* = 10) mice. The magenta boxed areas are shown at higher magnification on the right. (G) Representative images (upper images) and statistical analysis (lower graphs) of oxidized lipid-containing LDs in KL (*n* = 16) and KLG4^m/m^ (*n* = 17) CD45^−^CD31^−^EpCAM^+^ tumor cells. (H) Representative images (upper images) and statistical analysis (lower graphs) of oxidized lipid-containing LDs in KP (*n* = 12) and KPG4^m/m^ (*n* = 14) CD45^−^CD31^−^EpCAM^+^ tumor cells. Graphs show mean ± SEM (D–H). Statistical analyses were performed with multiple *t*-test (D) and two-tailed Student’s *t*-test (E–H). Scale bars represent 100 µm (E), 5 µm (white, F, G, and H) and 500 nm (blue, F); *n* = 3 mice per group (B–D). Data are representative of three independent experiments (E–H). *P < 0.05, **P < 0.01, ***P < 0.001, ****P < 0.0001.

Further analysis of our lipidomic data suggested that TAG (especially PUFA-TAG) was increased and diacylglycerol (DAG) was decreased in KLG4^m/m^ tumor cells compared to KL tumor cells (Fig. 2A–C; Table S1B). In addition, the results from targeted lipidomics assays suggested that oxidized TAG (oxTAG) was significantly increased in KLG4^m/m^ tumor cells compared to KL tumor cells (Fig. 2D; Table S1A). Consistent with the notion that cells convert excessive lipids (mainly TAG) into lipid droplets (Mathiowetz and Olzmann, 2024; Olzmann and Carvalho, 2019), we observed increased Oil Red O staining in KLG4^m/m^ tumor cells compared to KL tumor cells (Fig. 2E). In addition, the results from the ultrastructural section and TEM analysis revealed more and larger lipid droplets in KLG4^m/m^ tumor cells than in KL tumor cells (Fig. 2F). Notably, the lipid droplets contained more oxidized lipids in KLG4^m/m^ tumor cells than in KL tumor cells, as indicated by the C11-BODIOY staining (Fig. 2G), which is consistent with the observations that more oxTAG was identified in KLG4^m/m^ tumor cells than in KL tumor cells (Fig. 2D). Similarly, we also observed a higher abundance of lipid droplets containing oxidized lipid species in KPG4^m/m^ tumor cells than in KP tumor cells (Fig. 2H). Collectively, these data implicate that inducible deletion of GPX4 in tumor cells in the autochthonous NSCLC models leads to the synthesis and storage of TAG and oxTAG to evade ferroptosis. In support of this notion, it has been shown that lipid droplets protect the Drosophila glial cell niche and neural stem cells from harmful PUFA oxidation-induced ferroptosis (Bailey et al., 2015).

**Figure 3.**
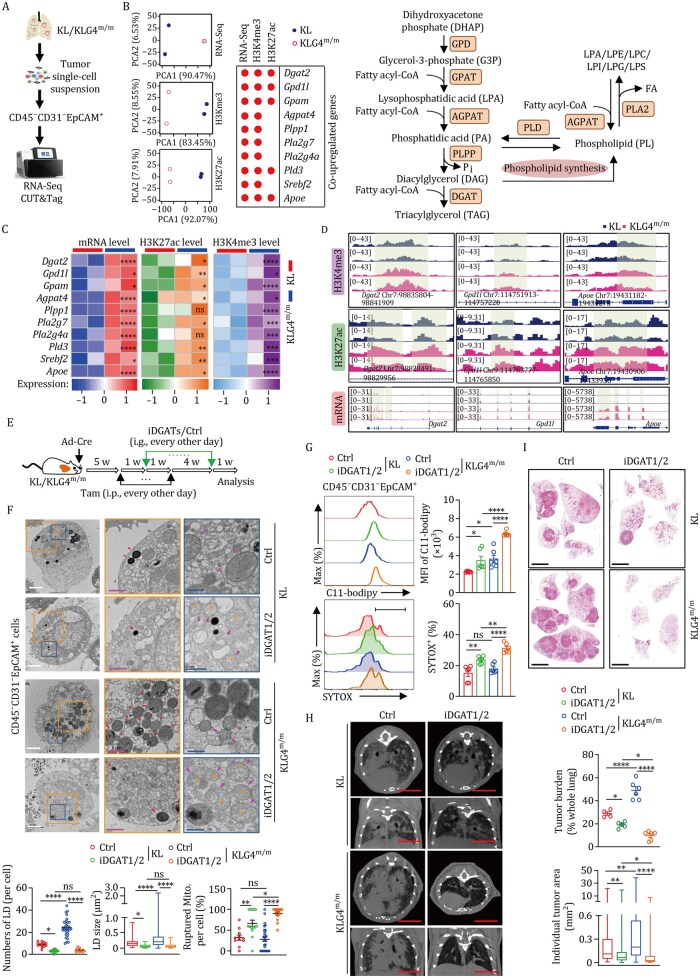
**Inhibition of TAG synthesis re-sensitizes *Gpx4*-deficient tumor cells to ferroptosis**. (A) A workflow scheme of RNA-seq and CUT&Tag (H3K4me3 and H3K27ac) of KL and KLG4^m/m^ CD45^−^CD31^−^EpCAM^+^ tumor cells. (B) The principal component analysis (PCA) plot showing the differentially expressed genes (DEGs) in RNA-Seq and the differential histone modifications in CUT&Tag assays of KL and KLG4^m/m^ CD45^−^CD31^−^EpCAM^+^ tumor cells (left). Dotplot showing the upregulated DEGs and the histone modifications in CUT&Tag assays of KL and KLG4^m/m^ CD45^−^CD31^−^EpCAM^+^ tumor cells (middle). Scheme of TAG and phospholipids synthesis pathway (right). GPD, glycerol-3-phosphate dehydrogenase; GPAT, glycerol-3-phosphate acyltransferase; AGPAT, 1-acylglycerol-3-phosphate O-acyltransferase; PLPP, phospholipid phosphatase; DGAT, diacylglycerol acyltransferase; PLD, phospholipase D; PLA2, phospholipase A2; FA, fatty acid. (C) Heatmap showing the expression levels, H3K4me3 and H3K27ac modifications of the 10 TAG synthesis-related DEGs. (D) Representative integrative genomics viewer (IGV) displaying CUT&Tag sequencing and RNA-sequencing signal profiles across *Dgat2*, *Gpd1l*, and *Apoe* loci in CD45^−^CD31^−^EpCAM^+^ tumor cells derived from lung tumors of KL and KLG4^m/m^ mice. (E) Schematic illustration depicts tumor induction and iDGAT1/2 (composed of T863 and PF06424439) treatment in KL and KLG4^m/m^ mice that were intranasally injected with Ad-Cre (2 × 10^6^ PFU per mouse) for 5 weeks followed by intraperitoneal injection of tamoxifen every other day for 2 weeks. One week after Tam treatment, the mice were injected with iDGAT1/2 (composed of T863 and PF06424439, 20 mg and 40 mg per kg body weight, respectively) every other day by gavage for 5 weeks. The mice were rested for 1 week followed by various analyses. (F) Representative images from transmission electron microscopy showing the morphology of lipid droplets (red arrowheads, in middle panel images) and mitochondria (bottom panel images, mitochondria were indicated by magenta arrowheads and ruptured mitochondria were highlighted with orange asterisks) along with statistics of the numbers and the sizes of lipid droplets (LDs) and the percentage of ruptured mitochondria in CD45^−^CD31^−^EpCAM^+^ tumor cells from lung tumors of KL and KLG4^m/m^ mice treated as in (E). The orange and blue boxed areas are shown at higher magnifications in the middle (orange box) and the bottom (blue box), respectively. (G) The levels of C11-bodipy staining (upper panels) and SYTOX staining (lower panels) of CD45^−^CD31^−^EpCAM^+^ tumor cells from lung tumors of KL and KLG4^m/m^ mice treated as in (E); *n *= 6 for each group. (H and I) Representative images of H&E staining (H, left), statistics of tumor burdens and individual tumor sizes (H, right) and representative images of micro-CT (I) of tumor-burdened lungs from the KL (*n *= 4 for control and *n *= 5 for iDGAT1/2, respectively) and KLG4^m/m^ (*n *= 6 for control and *n *= 6 for iDGAT1/2) mice treated as in (E); *n *= 2 mice per group (B–D). Graphs show mean ± SEM (F–H). Statistical analyses were performed with two-way ANOVA (F–H). Scale bars represent 2 µm (white, F), 1 µm (magenta, F), 500 nm (blue, F), and 5 mm (H and I). Data are representative of three (F–I) independent experiments. *P < 0.05, **P < 0.01, ***P < 0.001, ****P < 0.0001. ns, not significant.

To investigate the mechanism underlying the increase in TAG synthesis and storage in *Gpx4*-null tumor cells, we performed transcriptome sequencing (mRNA-seq) of KL and KLG4^m/m^ tumor cells (Fig. 3A), and found that the expression of genes involved in TAG synthesis such as *Gpd1l*, *Gpam*, *Agpat4*, *Plpp1*, *Dgat2* and *Srebf2* (Krahmer et al., 2009; Lee et al., 2024; Mathiowetz and Olzmann, 2024) was significantly upregulated in KLG4^m/m^ tumor cells compared to KL tumor cells (Fig. 3B and 3C; Table S2A), which was further confirmed by RT-qPCR assays (Fig. S5A and S5B), indicating that GPX4 reprograms the expression of genes involved in TAG metabolism. Consistent with this notion, analysis of scRNA-seq data suggested that tumor cells expressing high levels of *GPX4* mRNA (*GPX4*^high^) exhibited lower levels of *DGAT2*, *GPD1L*, and *APOE* mRNA than those expressing low levels of *GPX4* mRNA (*GPX4*^low^) in the human NSCLC tissues or the KL NSCLC mouse model (Hu et al., 2023; Wang et al., 2021) (Fig. S5C and S5D). Interestingly, however, the expression of *Dgat2* and *Apoe* became comparable between KLG4^m/m^ and KL tumor cells at 12 h after culture and was lower in KLG4^m/m^ tumor cells than in KL tumor cells at 24 h after culture (Fig. S5E). In addition, the expression of *Dgat2* and *Apoe* was not upregulated in subcutaneous syngeneic KLG4^m/m^ tumors compared to KL tumors (Fig. S5F and S5G), indicating that the microenvironment in the autochthonous NSCLC models licenses GPX4-mediated regulation of genes involved in TAG synthesis. These data suggest that the autochthonous *Gpx4*-null tumor cells upregulate the expression of *Dgat2* and adapt to (ox)TAG synthesis and lipid droplet formation.

### Knockout of GPX4 promotes H3K4me3 and K3K27ac modifications on *Dgat2* gene locus in autochthonous tumors

We next performed high-throughput Cleavage Under Targets and Tagmentation (CUT&Tag) sequencing assays (H3Kme3 or H3K27ac) to examine whether the transcriptional upregulation of genes involved in TAG metabolism was associated with epigenetic modifications on the loci of genes. The results suggested that the H3Kme3 and H3K27ac modifications on the locus of the *Dgat2* and *Gpd1l* genes in KLG4^m/m^ tumor cells were significantly increased compared to those in KL tumor cells (Fig. 3B–D; Table S3A and S3B), which was confirmed by ChIP-qPCR (chromatin immunoprecipitation–quantitative polymerase chain reaction) assays (Fig. S6A and S6B). In contrast, however, we found that the epigenetic markers (H3Kme3 or H3K27ac) on the loci of *Dgat2* and *Gpd1l* were comparable between the syngeneic KL and KLG4^m/m^ tumor cells or even lower in KLG4^m/m^ tumor cells than in KL tumor cells (Fig. S6C), consistent with the observations that the levels of *Dgat2* and *Gpd1l* were not upregulated in syngeneic KLG4^m/m^ tumor cells than in KL tumor cells (Fig. S5F and S5G). These data together suggest that inducible knockout of GPX4 in autochthonous tumor cells results in upregulation of (ox)TAG synthesis-related genes at both the epigenetic and the transcriptional levels.

### Inhibition of (ox)TAG synthesis sensitizes the autochthonous *Gpx4*-null tumor cells to ferroptosis

Diacylglycerol acyltransferase 1 and 2 (DGAT1/2) are rate-limiting enzymes in TAG synthesis that catalyze the conversion of DAG into TAG (Farese and Walther, 2023; Krahmer et al., 2009). T863 and PF06424439 are inhibitors of DGAT1 and DGAT2, respectively (collectively referred to as iDGAT1/2 hereafter) that substantially inhibit TAG synthesis (Lee et al., 2024; Wang et al., 2024). We further found that the injection of iDGAT1/2 by gavage significantly decreased the number of lipid droplets and promoted mitochondrial rupture in KL and KLG4^m/m^ tumor cells (Figs. 3E, 3F and S7A). Consistently, results from lipidomics and targeted lipidomics analyses suggested that TAG (especially PUFA-TAG) and oxTAG were substantially decreased in KL and KLG4^m/m^ tumor cells after iDGAT1/2 treatment (Fig. S7B–E; Table S1C). In addition, treatment with iDGAT1/2 increased levels of oxPE and oxPC and C11-BODIPY and SYTOX staining in KL and KLG4^m/m^ tumor cells (Figs. 3G and S7E; Table S1D), indicating that iDGAT1/2 promotes ferroptosis of autochthonous KL and KLG4^m/m^ tumor cells. Consistently, iDGAT1/2 attenuated tumor progression in the autochthonous KL and KLG4^m/m^ models (Fig. 3H and 3I). Notably, iDGAT1/2 promoted mitochondrial rupture, lipid oxidation (oxPE and oxPC), and cell death more potently in KLG4^m/m^ tumor cells than in KL tumor cells and inhibited tumor progression more extensively in KLG4^m/m^ mice than in KL mice (Figs. 3F–I, S7A and S7E; Table S1C and S1D). Taken together, these data indicate that the inhibition of (ox) TAG synthesis by iDGAT1/2 sensitizes KLG4^m/m^ tumor cells to ferroptosis and inhibits NSCLC progression in the autochthonous KL model.

### The autochthonous *Gpx4*-null tumor cells exhibit increased efflux of TAG and oxTAG

**Figure 4.**
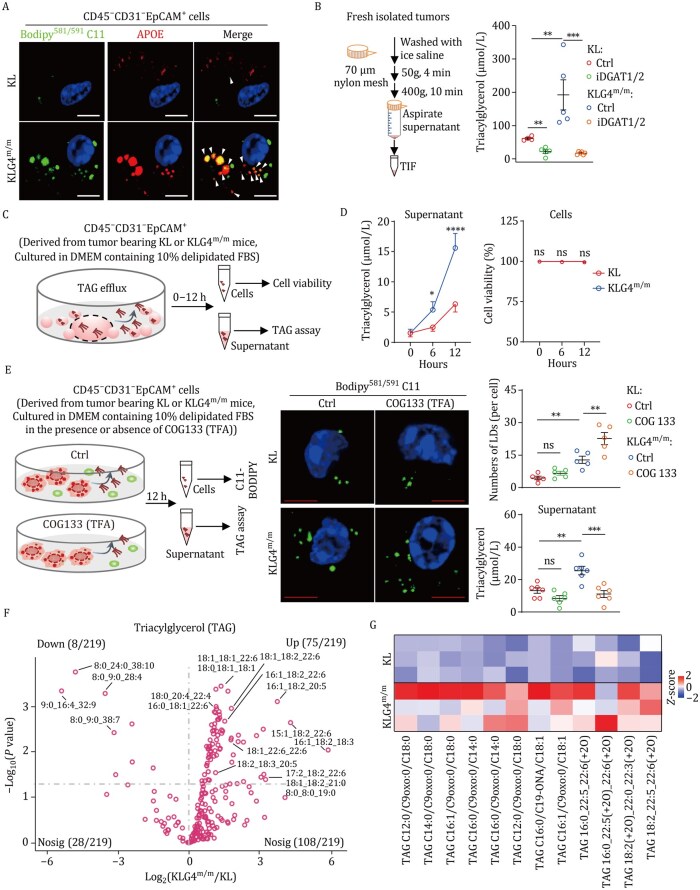
**The TAG efflux is elevated in *Gpx4*-null tumor cells of the autochthonous NSCLC models**. (A) Representative images of C11-bodipy and anti-APOE staining of CD45^−^CD31^−^EpCAM^+^ tumor cells from lung tumors of KL and KLG4^m/m^ mice. The white arrowheads indicated the simultaneous detection of Bodipy581/591 C11 (green) and APOE (red). (B) A scheme of tumor interstitial fluid (TIF) collection (left) and quantification of the TAG (right) in TIF from lung tumors of KL and KLG4^m/m^ mice that were treated as in Fig. 3E. KL, *n *= 4 and 5 for control and iDGAT1/2, respectively. KLG4^m/m^, *n *= 5 and 6 for control and iDGAT1/2, respectively. (C and D) A scheme diagram depicting the detection of TAG efflux (C) and the quantification of TAG efflux and cell viability (D) in CD45^−^CD31^−^EpCAM^+^ tumor cells from KL and KLG4^m/m^ mice (*n *= 4 for each group). (E) A scheme diagram (left) depicting CD45^−^CD31^−^EpCAM^+^ tumor cells from KL and KLG4^m/m^ mice treated with APOE inhibitor (COG133) for 12 h followed by Bodipy^581/591^ C11 staining and measurement of TAG in the culture supernatants. Representative images (middle images) and statistical analysis (top-right graph) of oxidized lipid-containing LDs in KL (*n *= 5 for Ctrl and COG133) and KLG4^m/m^ (*n *= 5 for Ctrl and COG133) CD45^−^CD31^−^EpCAM^+^ tumor cells, and quantification of the TAG (bottom-right graph) in culture supernatants from KL and KLG4^m/m^ CD45^−^CD31^−^EpCAM^+^ tumor cells with or without COG133 treatment (*n *= 6 per group). (F) Volcano plot showing the levels of triacylglycerols (TAG) in the supernatants of CD45^−^CD31^−^EpCAM^+^ tumor cells derived from KLG4^m/m^ mice (*n *= 5) compared to tumor cells derived from KL mice (*n *= 5). The tumor cells were cultured in a delipidated medium for 12 h, and the supernatants were harvested for lipidomics analysis. (G) Heatmap showing the representative oxidized TAGs (oxTAGs) in the supernatants of CD45^−^EpCAM^+^CD31^−^ tumor cells sorted from lung tumors of KL (*n *= 3) and KLG4^m/m^ (*n *= 3) mice. Each column represents *Z*-score normalized intensities of the detected oxTAG species. Graphs show mean ± SEM (B, D, and E). *P < 0.05, **P < 0.01, ***P < 0.001, ****P < 0.0001. ns, not significant. Statistical analyses were performed with one-way ANOVA (B and G) and two-way ANOVA (D). Scale bars represent 5 µm (A and E). Data are representative of three independent experiments (A, B, D, and E).

It has been recognized that liver cells synthesize very low-density apolipoprotein (APO) as the main carrier of TAG to promote the efflux of TAG (Getz and Reardon, 2009; Mensenkamp et al., 2004). Interestingly, we found that *Apoe* was more highly expressed in KLG4^m/m^ and KPG4^m/m^ tumor cells than in KL and KP tumor cells, respectively (Figs. 3B–D and S5A–D), and that a portion of APOE was colocalized with lipid droplets in KLG4^m/m^ and KL tumor cells (Fig. 4A). In addition, we observed elevated levels of TAG in the tumor interstitial fluid (TIF) of KLG4^m/m^ or KPG4^m/m^ tumors compared with those in KL or KP tumors, respectively (Figs. 4B and S8A), which were compromised by iDGAT1/2 treatment (Fig. 4B). The results from ultrastructural section and TEM analyses suggested the efflux of lipid droplets in KLG4^m/m^ and KL tumor cells (Fig. S8B), indicating that inducible knockout of GPX4 in tumor cells results in increased expression of APOE and efflux of TAG.

To further substantiate this notion, we isolated the KLG4^m/m^ or KPG4^m/m^ and the KL or KP tumor cells and cultured them in delipidated medium for 12 h followed by quantification and characterization of TAG in the supernatants (Figs. 4C and S8A). The results showed higher levels of TAG were detected in the supernatants of KLG4^m/m^ or KPG4^m/m^ tumor cells than in those of KL or KP tumor cells, respectively (Figs. 4D and S8A). Notably, the KLG4^m/m^ tumor cells did not die at 12 h after culture (Fig. 4D), which is consistent with the previous observations that deletion of GPX4 in cells results in ferroptosis at a later time (48–72 h) after culture (Friedmann Angeli et al., 2014). Treatment with APOE inhibitor COG133 (TFA) significantly downregulated the TAG levels in the supernatants of KLG4^m/m^ tumor cells and increased the lipid droplets in KLG4^m/m^ tumor cells (Fig. 4E). In addition, non-targeted and targeted lipidomic assays suggested that TAG and oxTAG levels were significantly increased in the supernatants of KLG4^m/m^ tumor cells compared to KL tumor cells (Fig. 4F and 4G; Table S1E and S1F). In contrast, we did not observe a dramatic increase in other ferroptosis-inducing lipid species, such as PC and PE, or FFAs (Doll et al., 2017; Liao et al., 2022; Qiu et al., 2024) in the supernatants of KLG4^m/m^ tumor cell cultures compared to the KL counterparts (Fig. S8C–E; Table S1E and S1F). Collectively, these data suggest that inducible knockout of GPX4 in the autochthonous KL NSCLC model upregulates the expression of APOE to promote the efflux of TAG and oxTAG.

### KLG4^m/m^ tumor cell-secreted lipids promote dysfunction and exhaustion of CD8^+^ T cells

Previous studies have shown that oxidized lipids impair CD8^+^ T cell effector functions in the TME (Ma et al., 2021; Xu et al., 2021). Consistent with the observation that KLG4^m/m^ tumor cells secreted higher levels of TAG and oxTAG than KL tumor cells did (Fig. 4C–G), the supernatants from KLG4^m/m^ tumor cell cultures more potently suppressed the production of TNFα, IFNγ, and granzyme B (GZMB) in P14 cells than those from KL tumor cell cultures in an *in vitro* acute activation model (Fig. S9A and S9B). Importantly, this suppression was abolished upon delipidation of the supernatants (Fig. S9A and S9B). In the chronic stimulation model involving repeated low-dose antigen exposure (Wu et al., 2023), culture supernatants from KLG4^m/m^ tumor cells promoted more robust upregulation of Tim3, PD-1, and TOX in P14 cells than those from KL tumor cells (Fig. S9C and S9D). This effect was reversed by the delipidation of the supernatants (Fig. S9C and S9D), indicating that GPX4-deficient tumor cells secrete lipid mediators that lead to dysfunction and exhaustion of CD8^+^ T cells.

### CD8^+^ T cells in the TME of KLG4^m/m^ tumors exhibit dysfunction and exhaustion

**Figure 5.**
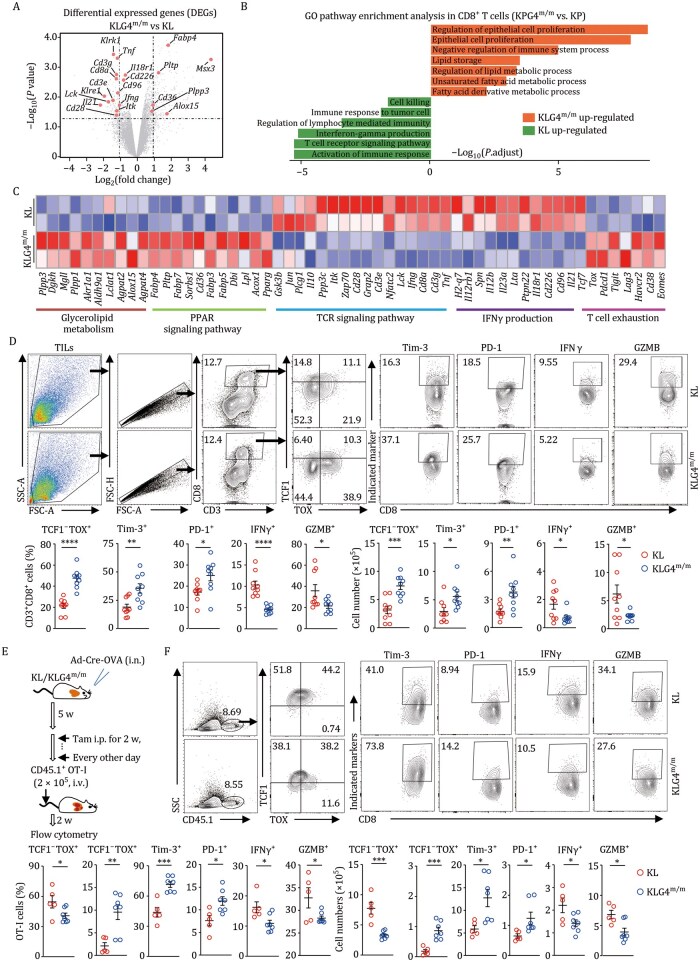
**Depletion of *Gpx4* in tumor cells of the autochthonous NSCLC models results in dysfunction of CD8^+^ T cells in the TME**. (A) Volcano plot of differentially expressed genes (DEGs, *P* < 0.05 and |log_2_(fold change)| ≥ 1) in tumor-infiltrated CD8^+^ T cells (CD8^+^ TILs) from KLG4^m/m^ (*n *= 2) and KL (*n *= 2) mice that were intranasally injected with Ad-Cre (2 × 10^6^ PFU per mouse) for 5 weeks followed by intraperitoneal injection of tamoxifen (Tam, 80 mg/kg, resolved in corn oil) every other day for 2 weeks and rest for 3 weeks. (B) Gene ontology (GO) analysis of the upregulated and downregulated genes (*P* < 0.05 and |fold change| ≥ 2) in CD8^+^ TILs from KLG4^m/m^ (*n *= 2) versus KL (*n *= 2) mice treated as in (A). (C) *Z*-score heatmap of representative genes from transcriptomic data of tumor-infiltrated CD8^+^ T cells from lung tumors of KLG4^m/m^ (*n *= 2) and KL (*n *= 2) mice treated as in (A). (D) Representative flow cytometry images (upper charts) and quantification analysis (lower graphs) of tumor-infiltrated lymphocytes (TILs) from lung tumors of KL (*n *= 9) and KLG4^m/m^ (*n *= 9) mice treated as in (A). (E) A scheme of tumor induction plus tamoxifen treatment of KL and KLG4^m/m^ mice followed by adaptive transfer of the naive CD45.1^+^ OT-I cells. KL and KLG4^m/m^ mice were intranasally injected with Ad-Cre (2 × 10^6^ PFU per mouse) for 5 weeks followed by intraperitoneal injection of tamoxifen (Tam, 80 mg/kg, resolved in corn oil) every other day for 2 weeks. Subsequently, CD45.1^+^ OT-I cells (2 × 10^5^ per mouse) were adoptively transferred into the mice for 2 weeks followed by flow cytometry analysis. (F) Representative flow cytometry images (upper charts) and quantification analysis (lower graphs) of the expression of TCF1, TOX, Tim-3, PD-1, IFNγ, and GZMB in OT-I cells from lung tumors of KL (*n *= 5) and KLG4^m/m^ (*n *= 7) mice treated as in (E). Graphs show mean ± SEM (graphs of D and E). Statistical analyses were performed using two-tailed Student’s *t*-test (D and E). Data are representatives of two independent experiments (D–F). *P < 0.05, **P < 0.01, ***P < 0.001, ****P < 0.0001.

Analysis of the transcriptomic data of CD8^+^ T cells in the TME of KLG4^m/m^ tumors revealed downregulated expression of genes involved in T cell activation pathways and upregulated expression of genes involved in T cell exhaustion compared to those in KL tumors (Figs. 5A–C and S10A; Tables S2B and S4), which was confirmed by RT-qPCR assays (Fig. S10B). Consistently, KLG4^m/m^ tumors showed significantly lower frequencies and absolute numbers of IFNγ^+^ and granzyme B^+^ (GZMB^+^) CD8^+^ T cells than KL tumors did (Fig. 5D). Furthermore, we observed increased proportions of exhausted TOX^+^TCF1^−^CD8^+^ T cells and upregulated surface expression of the exhaustion markers Tim-3 and PD-1 on CD8^+^ T cells (Huang et al., 2022) in KLG4^m/m^ tumors compared to KL tumors (Fig. 5D). Similarly, decreased IFNγ^+^CD8^+^ and GZMB^+^CD8^+^ T cells and increased TOX^+^TCF1^−^CD8^+^ T cells were found in KPG4^m/m^ tumors compared to KP tumors (Fig. S10C). In contrast, the IFNγ^+^CD8^+^ and GZMB^+^CD8^+^ T cells were comparable between the KLG4^m/m^ and the KL bronchial draining lymph nodes (Fig. S10D), indicating that inducible knockout of GPX4 in tumor cells results in CD8^+^ T cell dysfunction and exhaustion in the TME of KRas^G12D^ autochthonous NSCLC mice.

To examine whether CD8^+^ T cells were responsible for the tumor control in KL and KLG4^m/m^ mice, we induced tumors in KL and KLG4^m/m^ mice for 5 weeks followed by 2-week injection (i.p.) of tamoxifen and 5-week injection (i.p.) of αCD8α or the control IgG (Fig. S11A). Results from flow cytometry analysis demonstrated efficient depletion of CD8^+^ T cells in bronchial draining lymph nodes (dLNs) and spleens (Fig. S11B). Notably, depletion of CD8^+^ T cells substantially increased the burden and sizes in both KL and KLG4^m/m^ mice (Fig. S11C and S11D), indicating that CD8^+^ T cells are required to control tumor progression in the KL NSCLC model.

To examine whether tumor-specific CD8^+^ T cells in the TME were dysfunctional in the TME of KLG4^m/m^ tumors, we intranasally injected KL and KLG4^m/m^ mice with Ad-Cre-P2A-OVA virus for 5 weeks, followed by intraperitoneal injection of tamoxifen every other day for two successive weeks and by adoptive transfer of the CD45.1^+^ naive OT-I cells for 2 weeks (Tang et al., 2024) (Fig. 5E). The results from flow cytometry analyses revealed that the percentages and the absolute numbers of GZMB^+^ and IFNγ^+^ OT-I cells were significantly reduced in the TME of KLG4^m/m^ mice compared to those in KL mice (Fig. 5F). Moreover, compared to the counterparts from KL tumors, the OT-I cells from KLG4^m/m^ tumors showed increased TOX^+^TCF1^−^ subpopulations and upregulated expression of Tim3 and PD-1 (Fig. 5F). Collectively, these data demonstrate that inducible knockout of GPX4 in autochthonous KL tumor cells leads to dysfunction and exhaustion of antitumor CD8^+^ T cells to foster an immunosuppressive TME and promote NSCLC progression.

### Inhibition of DGAT1/2 restores CD8^+^ T cell function in the TME of KLG4^m/m^ tumors

**Figure 6.**
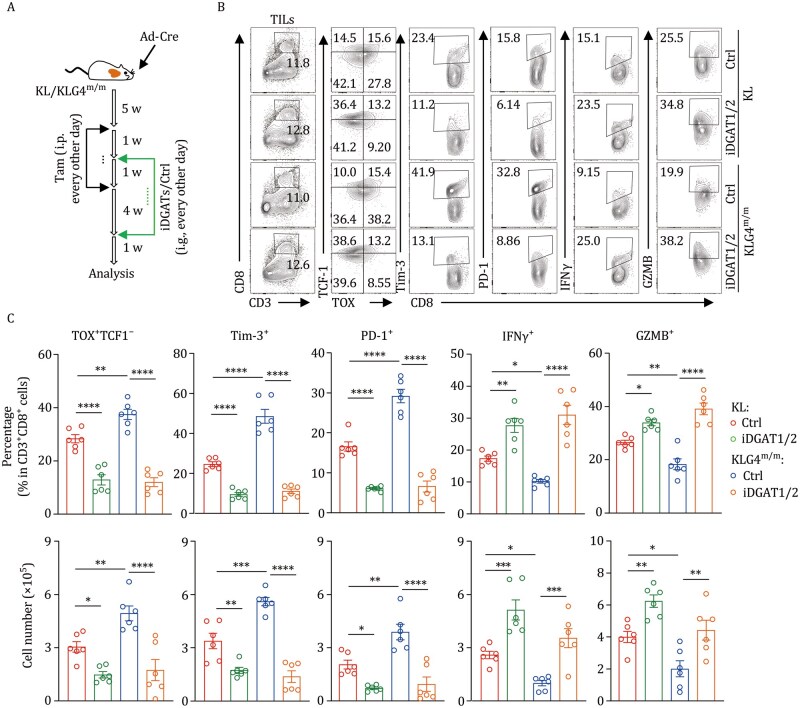
**Inhibition of DGAT1/2 restores CD8^+^ T cell function in the TME of KLG4^m/m^ tumors**. (A) Schematic illustration depicts tumor induction and iDGAT1/2 (composed of T863 and PF06424439) treatment in KL and KLG4^m/m^ mice that were intranasally injected with Ad-Cre (2 × 10^6^ PFU per mouse) for 5 weeks followed by intraperitoneal injection of tamoxifen every other day for 2 weeks. One week after Tam treatment, the mice were injected with iDGAT1/2 (composed of T863 and PF06424439, 20 mg and 40 mg per kg body weight, respectively) every other day by gavage for 5 weeks. The mice were rested for 1 week followed by various analyses. (B and C) Representative flow cytometry images (B) and quantification analysis (C) of tumor-infiltrated lymphocytes (TILs) from lung tumors of KL (*n *= 6 for iDGAT1/2 or control dissolvent) and KLG4^m/m^ (*n *= 6 for iDGAT1/2 or control dissolvent) mice treated as in (A) under indicated treatment. Graphs show mean ± SEM (C). *P < 0.05, **P < 0.01, ***P < 0.001, ****P < 0.0001. ns: not significant (two-way ANOVA for C). Data are representative results of two independent experiments (B and C).

We next examined whether treatment with iDGAT1/2 restored CD8^+^ T cell functions in the TME of KLG4^m/m^ mice (Fig. 6A). The results from flow cytometry analyses suggested that treatment with iDGAT1/2 significantly increased the intracellular levels of IFNγ and GZMB in and attenuated the expression of PD-1 and Tim-3 on CD8^+^ T cells from KLG4^m/m^ tumors (Fig. 6B and 6C). In addition, the percentages and the absolute numbers of exhausted TCF1^−^TOX^+^ CD8^+^ T cells in the TME of KLG4^m/m^ mice were significantly reduced after treatment with iDGAT1/2 (Fig. 6B and 6C). Notably, iDGAT1/2 promoted the expression of IFNγ and GZMB in CD8^+^ T cells and reduced the exhaustion of CD8^+^ T cells from KLG4^m/m^ tumors to a level similar to that in those from KL tumors (Fig. 6B and 6C), suggesting an essential role of (ox)TAG in the dysfunction and exhaustion of CD8^+^ T cells in the TME. Taken together, these findings indicate that KLG4^m/m^ tumor cells synthesize and secrete TAG (and oxTAG) to promote the dysfunction and exhaustion of CD8^+^ T cells in the TME.

### Inducible expression of GPX4 in tumor cells coordinates an immune-active TME and inhibits NSCLC progression

**Figure 7.**
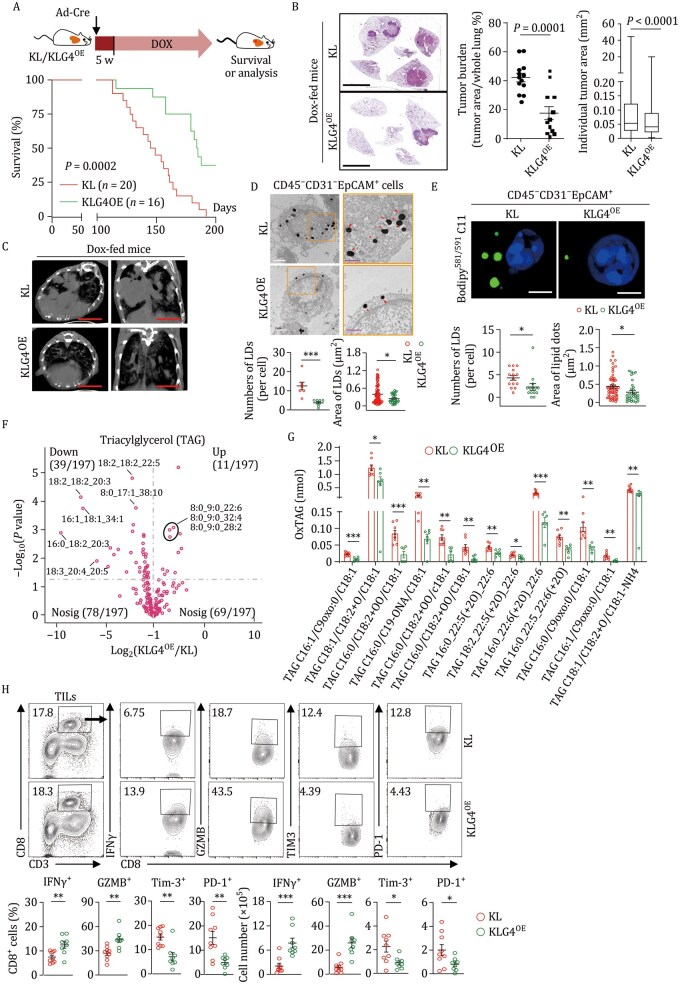
**Inducible overexpression of *Gpx4* in tumor cells alleviates NSCLC progression**. (A) A scheme of tumor induction (upper) and the survival (lower) of *Kras*^LSL-G12D/+^*Lkb1*^fl/fl^ (KL, *n* = 20) and *Kras*^LSL-G12D/+^*Lkb1*^fl/fl^*Gpx4*^OE^ (KLG4^OE^, *n* = 16) mice that were intranasally injected with Ad-Cre (2 × 10^6^ PFU/mice) for 5 weeks followed by feeding with Dox-supplemented chow diet until the endpoint of study. (B and C) Representative images of H&E staining (b, left) and statistics of tumor burdens (b, middle) and individual tumor sizes (b, right) and micro-CT (c) of tumor-burdened lungs from the KL (*n* = 7) and KLG4^OE^ (*n* = 9) mice treated as in (A). (D) Representative images from transmission electron microscopy (upper) and quantification of lipid droplets (LDs) (lower graphs) of CD45^−^CD31^−^EpCAM^+^ tumor cells from lung tumors of KL (*n* = 7) and KLG4^OE^ (*n* = 8) mice treated as in (A). The orange boxed areas were shown at higher magnification in the bottom images, in which arrowheads indicated LDs. (E) Representative images of confocal microscopy (upper) and quantification of the numbers and sizes (lower graphs) of oxidized lipids-containing LDs in CD45^−^CD31^−^EpCAM^+^ tumor cells from KL and KLG4^OE^ mice (*n* = 14 per group) treated as in (A). (F) Volcano plot displaying the triacylglycerols (TAGs) in the culture supernatant of CD45^−^CD31^−^EpCAM^+^ tumor cells from KLG4^OE^ mice (*n* = 6) compared to those from KL mice (*n* = 8). (G) Quantitative assessment showing the oxidized triacylglycerols (oxTAG) in the supernatant of CD45^−^CD31^−^EpCAM^+^ tumor cells culture from lung tumors of KL (*n* = 8) and KLG4^OE/+^ (*n* = 6) mice treated as in (A). (H) Representative images and quantitative analysis of flow cytometry assays of TILs from lung tumors of KL (*n* = 9) and KLG4^OE^ (*n* = 8) mice treated in (A). Graphs show mean ± SEM (B, D, E, G, and H). Statistical analysis was performed using log-rank analysis (A), two-tailed Student’s *t*-test (B, D, E, G, and H), or multiple *t*-tests (G). Scale bars represent 5 mm (B and C), 2 µm (white, D), 1 µm (magenta, D), or 5 µm (E). Data are combined results of three independent experiments (A) or representative results of two independent experiments (B–E, H). *P < 0.05, **P < 0.01, ***P < 0.001.

Having demonstrated that inducible knockout of GPX4 in tumor cells ignites an immunoinhibitory TME to promote NSCLC progression, we next investigated whether inducible expression of GPX4 in tumor cells would enhance antitumor immunity in the TME and inhibit the progression of NSCLC. To test this idea, we generated a line of mice in which GPX4 was inducibly overexpressed in tumor cells (termed G4^OE^ mice hereafter) based on Cre-loxP and tetracycline-controlled (tet-on) gene expression systems (Fig. S12A). PCR analysis of the tail genomic DNA indicated that the targeting vector was successfully knocked into the *H11* site (Fig. S12B). GPX4 was inducibly overexpressed only after Cre recombinase-mediated removal of the STOP cassette and in the presence of doxycycline (Dox) (Fig. S12C and S12D). Next, we obtained the *Kras*^LSL-G12D/+^*Lkb1*^fl/fl^*Gpx4*^OE^ (KLG4^OE^) mice and induced tumorigenesis in these mice by intranasal injection of Ad-Cre followed by feeding with Dox-supplemented food (Fig. 7A), and found that GPX4 was selectively upregulated in CD45^−^EpCAM^+^CD31^−^ tumor cells but not in endothelial cells (CD45^−^CD31^+^CD49d^−/int^), stromal cells (CD45^−^CD31^int^CD49d^+^) or CD45^−^CD31^−^EpCAM^−^ cells in tumors, or other organs (including the liver, spleen, kidney, and brain) from KLG4^OE^ mice fed Dox-supplemented food compared to those fed normal food (Fig. S12E and S12F), suggesting efficient, specific and inducible overexpression of GPX4 in tumor cells in the KLG4^OE^ autochthonous NSCLC mouse model.

We next assessed the effects of inducible GPX4 overexpression in tumor cells on NSCLC progression with the KLG4^OE^ mice. As shown in Fig. 7A, the KLG4^OE^ mice survived significantly longer than the KL mice did. The results of histological analysis and micro-CT imaging suggested that the total tumor burden and the individual tumor size were significantly reduced in the lungs of KLG4^OE^ mice compared to those in the lungs of KL mice (Fig. 7B and 7C), suggesting that inducible overexpression of GPX4 in tumor cells inhibits the progression of NSCLC in the autochthonous KL mouse model. Interestingly, genes involved in TAG synthesis and efflux, such as *Dgat1*/*2*, *Gpd1l*, *Gpam*, and *Apoe*, were significantly downregulated in the KLG4^OE^ tumor cells compared to the KL tumor cells (Fig. S12G), and the lipid droplets in KLG4^OE^ tumor cells were substantially less and smaller than in KL tumor cells (Fig. 7D), which aligns with the observation that higher *GPX4* mRNA levels correlated with lower mRNA levels of *DGAT2*, *GPD1L*, and *APOE* in human NSCLC tissue (Fig. S5C). Notably, the lipid droplets contained fewer oxidized lipids in KLG4^OE^ tumor cells than in KL tumor cells, as indicated by the C11-BODIPY staining (Fig. 7E). Consistently, fewer TAG and oxTAG were produced by the KLG4^OE^ tumor cells than the KL tumor cells (Fig. 7F and 7G; Table S1G and S1H). These data suggest that inducible overexpression of GPX4 in tumor cells reduces the synthesis and efflux of TAG and oxTAG.

We further found that the expression of intracellular IFNγ and GZMB in CD8^+^ T cells was potentiated, whereas the surface exhaustion markers Tim-3 and PD-1 were reduced in CD8^+^ T cells from the TME of KLG4^OE^ tumors compared to those from the control counterparts (Fig. 7H), which aligns with the TCGA and scRNA-seq data showing that high expression of *GPX4* is correlated with increased survival of pancreatic ductal adenocarcinoma patients (Dai et al., 2020) and decreased T cell exhaustion score in human NSCLC tissue (Hu et al., 2023; Maroni et al., 2021; Wu et al., 2021; Yan et al., 2025) (Fig. S13). Collectively, these data suggest that inducible overexpression of GPX4 in tumor cells promotes the antitumor functions of CD8^+^ T cells to inhibit NSCLC progression.

## Discussion

It has been demonstrated that knockout of GPX4 in cancer cell lines results in ferroptosis and that inducible deletion of GPX4 in mice causes renal failure and death within 2 weeks (Friedmann Angeli et al., 2014). However, our results showed that inducible deletion of GPX4 in tumor cells in the KL and KP autochthonous NSCLC mouse models had little effect on the ferroptosis of tumor cells, indicating that the tumor cells in these models adapt to GPX4 deficiency-induced ferroptotic stress. In support of this notion, we found that the peroxidation of PE or PC, whose products are classical ferroptosis inducers (Doll et al., 2019; Friedmann Angeli et al., 2014; Kim et al., 2022; Li et al., 2024), was comparable between the KLG4^m/m^ and the KL tumor cells. In contrast, genes involved in TAG synthesis, such as *Gpd1l*, *Gpam*, *Agpat4*, *Plpp1*, *Dgat2*, and *Srebf2*, were upregulated, and the levels of TAG and oxTAG and the lipid droplets were increased in KLG4^m/m^ tumor cells compared to KL tumor cells. In addition, inhibition of DGAT1/2 significantly downregulated the levels the PUFA-TAG and oxTAG and upregulated the levels of oxPE and oxPC in KL and KLG4^m/m^ tumor cells, indicating that the GPX4-DGAT1/2 axis reprograms ferroptotic oxPE/PC metabolism to oxTAG synthesis and thereby inhibits ferroptosis of KL and KLG4^m/m^ tumor cells. Together with the observations that TAG biosynthesis confers resistance to ferroptosis (Bailey et al., 2015; Lee et al., 2024; Ping et al., 2024), these findings suggest that tumor cell-specific GPX4 deficiency reprograms TAG metabolism to evade ferroptosis in autochthonous NSCLC mouse models (Fig. S14). When our study was in the final revision process, a publication reported that sgRNA-mediated knockout of *Gpx4* inhibited NSCLC progression in the *Kras*^LSL-G12D/+^*Tp53*^fl/fl^*Rosa26*^LSL-Cas9/LSL-Cas9^ mouse model in which *Gpx4* was deleted at the time of KRas^G12D^ expression (Wu et al., 2025). While KRas^G12D^-induced malignant transformation requires 2–3 weeks (Jackson et al., 2001; Johnson et al., 2001), acute deletion of GPX4 results in ferroptosis within 48–72 h (Friedmann Angeli et al., 2014). Therefore, such an early deletion of *Gpx4* would result in the death of normal and incompletely transformed epithelial cells, which is expectedly to inhibit NSCLC progression and might be the possible reason for the discrepancies between that study and ours.

In the syngeneic graft mouse models, however, knockout or inducible knockout of GPX4 in tumor cells resulted in compromised growth and potentiated ferroptosis that was restored by Lip-1, which is consistent with previous reports (Kim et al., 2022; Li et al., 2024; Yang et al., 2014). It should be noted that the TME between the autochthonous models and the syngeneic models is different, which might be responsible for the opposite phenotypes of the tumor cell-specific GPX4-knockout syngeneic and autochthonous NSCLC models. For the syngeneic model, a large number of tumor cells are inoculated subcutaneously, and few immune cells are identified within the tumors. In contrast, in the autochthonous NSCLC models and human NSCLC tissues, the tumor cells originate from a few mutated malignant cells, undergo constant immune editing by the immune cells in the TME, and account for a small portion of the whole tumor tissue (Wang et al., 2021; Zilionis et al., 2019). Although the exact TME factors responsible for the distinct behaviors between GPX4-deficient autochthonous and syngeneic lung tumors are unclear, our study highlights the caution in interpreting the syngeneic models that are extensively used to evaluate the pro- or anti-tumor activity of a specific gene or compound.

We observed that knockout of GPX4 in autochthonous KL tumor cells, but not in subcutaneous syngeneic KL tumor cells or normal lung epithelial cells, led to the upregulation of *Dgat2* and *Gpd1l* mRNA levels, and such expression difference of *Dgat2* and *Gpd1l* mRNA between the autochthonous KL and KLG4^m/m^ tumor cells was gradually diminished in *in vitro* cultures, indicating that factors in the TME license the upregulation of genes involved in TAG metabolism. It should be noted that the lung tumor identity may be altered during *in vitro* culture or subcutaneous transplantation, characterized by the loss of lineage-specific markers such as TTF-1 (also known as NKX2‑1) after the removal from the pulmonary microenvironment (Camolotto et al., 2018; Snyder et al., 2013). Because TTF-1 interacts with transcriptional factors Foxa1 and Foxa2 that bind to lung-specific loci to maintain pulmonary identity, the loss of TTF-1 might result in an altered binding of Foxa1/2 to genes involved in TAG synthesis (Minoo et al., 2007; Snyder et al., 2013). In this context, it has been demonstrated that Foxa1 is a potent inhibitor of hepatic TAG synthesis by repressing the expression of genes such as *GPAM* and *DGAT2* (Moya et al., 2012). In addition, we observed that H3K4me3 and H3K27ac modifications on the loci of *Dgat2* and *Gpd1l* were significantly increased in autochthonous KLG4^m/m^ tumor cells but not in syngeneic KLG4^m/m^ tumor cells compared to the respective KL counterparts, indicating that the epigenetic modification factors are involved in such differential regulatory processes. In support of this notion, we found that the autochthonous KLG4^m/m^ tumor cells produced higher amounts of TAG and oxTAG and exported them into the extracellular space, leading to CD8^+^ T-cell functional impairment, which was substantially inhibited by iDGAT1/2. A thorough understanding of how GPX4 coordinates the identity-maintaining machinery and epigenetic factors in autochthonous versus syngeneic to reprogram TAG metabolism requires further investigation. Nonetheless, these data indicate that TAG and oxTAG from tumor cells shape an immunosuppressive TME to promote NSCLC progression.

Our data demonstrate that inducible overexpression of GPX4 in tumor cells ignites an immune-active TME characterized by potentiated activation of CD8^+^ T cells, leading to compromised NSCLC progression of the autochthonous models. Together with the observations that lipid peroxidation suppresses CD8^+^ T cell effector functions (Ma et al., 2021; Wang et al., 2020; Xu et al., 2021), boosting the expression or the function of GPX4 in the TME would be a benefit for the NSCLC patients. In this context, it has been reported that high expression of GPX4 is correlated with increased survival in pancreatic ductal adenocarcinoma (PDAC) patients (Dai et al., 2020), and the levels of *GPX4* mRNA were negatively correlated with the levels of *DGAT2*, *GPD1L*, and *APOE* mRNA in human NSCLC tumor cells and the T cell exhaustion score in human NSCLC tumor tissues. In addition, we provided evidence that inhibition of TAG synthesis via iDGAT1/2 (Lee et al., 2024; Wang et al., 2024) significantly decreased the synthesis and the efflux of TAG (oxTAG) and the accumulation of lipid droplets in tumor cells. Accordingly, iDGAT1/2 treatment sensitizes tumor cells to ferroptosis and attenuates tumor progression in the autochthonous NSCLC models. Notably, two phase 2 clinical trials have shown high safety and efficacy of the DGAT2 inhibitor (ervogastat, PF-06865571) in non-alcoholic fatty liver disease metabolism (Calle et al., 2021). It is worth noting the future clinical use of ervogastat and PF-06865571 in NSCLC. Taken together, our findings reveal the preclinical outcomes of specific ablation of GPX4 in tumor cells and highlight potential therapeutic interventions for NSCLC patients.

## Supplementary Material

pwaf101_Supplementary_Data

## Data Availability

The RNA-seq data and CUT&Tag data have been deposited in the Gene Expression Omnibus (GEO) under accession codes GSE281154 and GSE287544, respectively, which can be found in Tables S2 and S3. All requests for materials and data should be directed to the corresponding authors.

## Code availability

This paper does not report the original code.
