# Supplementary material for: Tumor cell-specific loss of GPX4 reprograms triacylglycerol metabolism to escape ferroptosis and impair antitumor immunity in non-small cell lung cancer
Source: Protein Cell. 2025 Nov 19;17(5):421–37. doi: 10.1093/procel/pwaf101 (PMC13161477; doi:10.1093/procel/pwaf101)
Supplement: pwaf101_Supplementary_Data [file pwaf101_supplementary_data.zip › PAC-25311-ZB-Supplementary materials.pdf]

Methods

Mice

The *Kras*$^{\text{LSL-G12D/+}}$ (#008179), the *Tp53*$^{\text{fl/fl}}$ (#008462), and the *Lkb1*$^{\text{fl/fl}}$ (#014143) mice were purchased from the Jackson Laboratory and crossed to obtain the *Kras*$^{\text{LSL-G12D/+}}$*Tp53*$^{\text{fl/fl}}$ (KP) and the *Kras*$^{\text{LSL-G12D/+}}$*Lkb1*$^{\text{fl/fl}}$ (KL) strains as previously described (Dong et al., 2023; Wang et al., 2021; Zhang et al., 2020). The *Kras*$^{\text{LSL-G12D/+}}$*Tp53*$^{\text{fl/fl}}$*Gpx4*$^{\text{m/m}}$ (KPG4$^{\text{m/m}}$) and the *Kras*$^{\text{LSL-G12D/+}}$*Lkb1*$^{\text{fl/fl}}$*Gpx4*$^{\text{m/m}}$ (KLG4$^{\text{m/m}}$) mice were generated by GemPharmatech following these steps. The eggs from wild-type C57BL/6 mice were fertilized *in vitro* with sperm from KP or KL mice, both on the C57BL/6 background. The targeting vector containing the rox-*Gpx4* (exon 5 to exon 7)-rox-DreER$^{\text{T2}}$-loxP2272-STOP-loxP2272-CAG promoter and the flanking homologous sequences of the *Gpx4* gene locus, along with the gRNA-Cas9 complex, was subsequently injected into the zygotes to obtain the *Kras*$^{\text{LSL-G12D/+}}$*Tp53*$^{\text{fl/+}}$*Gpx4*$^{\text{m/+}}$or the *Kras*$^{\text{LSL-G12D/+}}$*Lkb1*$^{\text{fl/+}}$*Gpx4*$^{\text{m/+}}$ mice. Since the *Gpx4* gene locus is approximately 60 kb upstream of the *Lkb1* gene locus, which contains two loxp66 sites (5'-ATAACTTCGTATAGCATACATTATACGAAGTTAT-3'), two distinct Cre recombinase recognition sites, loxP2272 sites (5'-ATAACTTCGTATAAAGTATCCTATACGAAGTTAT-3') flanking the STOP cassette within the *Gpx4* locus, were introduced into the targeting vector to ensure the correct removal of the STOP cassette and the *Lkb1* exon 3 to 6 in the presence of Cre recombinase. The F0 *Kras*$^{\text{LSL-G12D/+}}$*Tp53*$^{\text{fl/+}}$*Gpx4*$^{\text{m/+}}$ or *Kras*$^{\text{LSL-G12D/+}}$*Lkb1*$^{\text{fl/+}}$*Gpx4*$^{\text{m/+}}$ mice were then crossed with KP or KL mice for at least five generations to obtain the *Kras*$^{\text{LSL-G12D/+}}$*Tp53*$^{\text{fl/fl}}$*Gpx4*$^{\text{m/+}}$ (KPG4$^{\text{m/+}}$) or the *Kras*$^{\text{LSL-G12D/+}}$*Lkb1*$^{\text{fl/fl}}$*Gpx4*$^{\text{m/+}}$ (KLG4$^{\text{m/+}}$) mice, respectively. The KPG4$^{\text{m/+}}$ or KLG4$^{\text{m/+}}$ mice were crossed to obtain the

Kras$^{\text{LSL-G12D/+}}$*Tp53*$^{\text{fl/fl}}$*Gpx4*$^{\text{m/m}}$ (KPG4$^{\text{m/m}}$) or *Kras*$^{\text{LSL-G12D/+}}$*Lkb1*$^{\text{fl/fl}}$*Gpx4*$^{\text{m/m}}$ (KLG4$^{\text{m/m}}$) mice for maintenance and experiments, respectively. In addition, the F0 *Tp53*$^{\text{fl/+}}$*Gpx4*$^{\text{m/+}}$ mice were crossed with wild-type C57BL/6 mice for at least three generations to obtain the *Gpx4*$^{\text{m/+}}$ mice that were crossed to generate the *Gpx4*$^{\text{m/m}}$ mice for maintenance and experiments.

The *Gpx4*^{OE} mice were generated by GemPharmatech through CRISPR/Cas9-mediated gene editing. In brief, the targeting vector consisting of pTRE3G promoter-loxp-STOP-loxp-m*Gpx4* (NM_001367995.1)-polyA-rTTA-CAG promoter and the flanking homologous sequences of the *H11* site and the gRNA-Cas9 RNPs were injected into the in vitro obtained zygotes of wild-type C57BL/6 mice. The F0 *Gpx4*^{OE} mice were crossed with the KL mice to obtain the *Kras*$^{\text{LSL-G12D/+}}$*Lkb1*$^{\text{fl/+}}$*Gpx4*^{OE} that were crossed with the KL mice for at least six generations to obtain the *Kras*$^{\text{LSL-G12D/+}}$*Lkb1*$^{\text{fl/fl}}$*Gpx4*^{OE} (KLG4^{OE}) mice for maintenance and experiments. Alternatively, the F0 *Gpx4*^{OE} mice were crossed with wild-type C57BL/6 mice for three generations to obtain the *Gpx4*^{OE} mice that were used for experiments.

The CD45.1^{+} OT-I mice carrying a transgenic TCR specific for H-2K^{b} and OVA$_{257–264}$ were purchased from Cyagen Biosciences (Suzhou, China). The wild-type C57BL/6 mice were purchased from GemPharmatech Co., Ltd (Nanjing, China). The sequences of gRNAs were 5’-GGCTGTCTTCCGGCCTTGGA-3’ and 5’-TGCATGCTTGAAGCCCTCCA-3’ for *Gpx4*$^{\text{m/+}}$ mice, and 5’-CTGAGCCAACAGTGGTAGTA-3’ for *Gpx4*^{OE} mice, respectively.

No statistical methods were used to predetermine the sample size. For all experiments presented in this study, age- and sex-matched mice were used and the sample sizes were large enough to determine statistically significant effects. The control and experimental groups of mice were cohoused and randomly allocated to different treatments. All mice were housed in

the specific pathogen-free animal facility at Medical Research Institute, Wuhan University with a housing temperature of 22±1°C and relative humidity of 50-60 % with a 12-hour dark/12-hour light cycle and fed with standard food and water otherwise indicated. All animal experiments were performed in accordance with protocols approved by the Institutional Animal Care and Use Committee of Wuhan University (Approval No.21020A).

Genotyping

Genomic DNA was prepared from the tails of 4-week-old mice. The tissues were lysed in the lysis buffer (0.5 M Tris-HCl, pH 8.5, 5 mM EDTA, 0.2 % SDS, and 0.8 μg/μL proteinase K) for overnight at 65°C. After incubation, the samples were centrifuged at 10,000 g for 10 min at room temperature to obtain a supernatant containing the genomic DNA. The supernatant was transferred to a new 1.5 mL Eppendorf tube containing 1 mL 100% ethanol and mixed thoroughly. Subsequently, the mixtures were centrifugated at 10,000 g for 10 min at room temperature. The supernatants were discarded, and the resulting precipitants containing DNA were dissolved in deionized water for subsequent PCR analysis. The PCR cycling conditions were as follows: 94ºC for 2 min; 94ºC for 30 seconds; 58ºC (61ºC for *Kras*) for 30 seconds; 72ºC for 30 seconds; repeat steps 2 through 4 for a total of cycles; 72ºC for 5 min; 16ºC for 10 min, unless indicated otherwise. The PCR products were analyzed by agarose gel electrophoresis as previously described (Dong et al., 2023; Wang et al., 2021; Zhang et al., 2020). The primers used for genotyping are summarized in Table S5.

Cell culture and *in vitro* treatment

Primary mouse lung fibroblasts (MLFs) were isolated from ~8-10-week-old mice. Lungs were minced and digested in calcium and magnesium-free HBSS buffer supplemented with 10 mg/mL type I collagenase (Worthington) and 20 μg/mL DNase I (Sigma-Aldrich) for 2.5 h at a cell culture incubator with intervals of pipette. The cell suspensions were centrifuged and filtered through a 70 μm nylon mesh followed by culture in DMEM containing 15% FBS (v/v), 1% streptomycin–penicillin. Two days later, the adherent fibroblasts were rinsed with pre-warmed PBS and cultured in a 6-well plate for subsequent experiments.

For MLFs derived from $Gpx4^{+/+}$ and $Gpx4^{m/m}$ mice, cells were infected with Ad-Cre for 48 h followed by treatment with 4-hydroxytamoxifen (4OHT, 1μM, Sigma, Cat# H6278) for 48 h. The cells were harvested for immunoblot analysis. For MLFs derived from $Gpx4^{+/+}$ and $Gpx4^{OE}$ mice, cells were infected with Ad-Cre, one day later, Doxycycline (20 μg/mL) was added to the cell medium for another 3 days. The cells were harvested for RT-qPCR and immunoblot analysis.

Preparation and infection of Ad-Cre-P2A-OVA viruses

The pDC316-mCMV-CRE-P2A-OVA vector (DesignGene, Shanghai) was co-transfected with the package plasmid pBHGlox(delta)E1,3Cre into HEK293A cells for 10 h (Tang et al., 2024). After transfection, the medium was replaced with fresh DMEM (supplemrnted with 10% FBS and 1% streptomycin-penicillin) and cells were cultured for an additional five days. Following incubation, cells were harvested via centrifugation at 3,000 g for 5 min, resuspended in DMEM, and subjected to three freeze-thaw cycles at -80°C and 37°C. The viruses in the supernatants were collected by centrifugation at 15,000 g for 10 min, followed

by three rounds of infection and collection from HEK293A cells. The resulting supernatants, containing Ad-Cre-OVA viruses, was filtered, collected, and stored at -80°C. To determine viral titers, aliquots or serial dilutions of the Ad-Cre-OVA viruses were used to infect 3T3$^{LSL-RFP}$ cells (kindly provided by Dr. Hong-Bin Ji, Chinese Academy of Science, Shanghai) for 48 h, followed by flow cytometry analysis to determine the titers. For tumor induction, age- and sex-matched KL/KLG4$^{m/m}$ mice were intranasally administered with Ad-Cre-P2A-OVA (5×10^6 PFU per mouse), and 5 weeks later, the mice were intraperitoneally injected with tamoxifen every other day for 2 weeks. The mice were either rested for 3 weeks followed by histological analyses or rested for 2 weeks followed by adoptive transfer of CD45.1$^+$ OT-I T cells (2×10^5 per mouse) and analyzed after an additional 2 weeks.

Induction of the autochthonous NSCLC tumors

The experiments were performed as previously described (Dong et al., 2023; Wang et al., 2021; Zhang et al., 2020). 8-10-week-old, sex-matched KL/KLG$^{m/m}$, KP/KPG4$^{m/m}$ or KL/KLG4^{OE} mice were anesthetized by intraperitoneal injection of 0.7% sodium pentobarbital (w/v, 10 μL/g body weight), followed by intranasal injection of Ad-Cre viruses (HANBIO, Shanghai) (2×10^6 PFU in 60 μl PBS per mouse) or Ad-SPC-Cre (5×10^6 PFU in 60 μL PBS per mouse) (kindly provided by Dr. Hongbin Ji, Chinese Academy of Science, Shanghai)(Li et al., 2015) for 5 weeks. Subsequently, the KL/KLG4$^{m/m}$ and KP/KPG4$^{m/m}$ mice were intraperitoneal injected with Tamoxifen (Tam, 10 mg/mL dissolved in corn oil and 80 mg/kg body weight) every other day for 2 weeks and rested until death for survival analysis, or rested for another 3 weeks for subsequent analysis. The KL/KLG4^{OE} mice were fed with

normal food until the fifth week after Ad-Cre injection. The mice were fed with Dox-supplemented food (+Dox) until death for survival analysis or for 8 weeks for subsequent analysis.

Treatment with iDGAT1/2 in autochthonous KL NSCLC mouse models

The KL/KLG4$^{m/m}$ mice were intranasally injected with Ad-Cre and administered tamoxifen intraperitoneally for two weeks as described above. One week after initiating the tamoxifen treatment, the mice were randomly assigned to different treatment groups, and followed by iDGAT1/2 treatments. The iDGAT1/2, consisting of T863 (a DGAT1 inhibitor(Lee et al., 2024; Wang et al., 2024), 20mg per kg of body weight, HY-32219, MedChemExpress) and PF-06424439 (a DGAT2 inhibitor(Wang et al., 2024), 40mg per kg of body weight, HY-108341, MedChemExpress), were solubilized in a solvent containing 5% DMSO, 30%PEG300, 5% Tween-80 and 60% H_2O. The iDGAT1/2 and the control were administered by gavage every other day and continued for 5 weeks. One week later, the mice were anesthetized for micro-CT scanning, and the lungs from tumor-bearing mice were collected for subsequent histological analysis or flow cytometry.

CD8$^+$ T cell depletion in tumor-bearing KL/KLG4$^{m/m}$ mice

The KL and KLG4$^{m/m}$ mice were intranasally injected with Ad-Cre for 5 weeks followed by intraperitoneal administration of tamoxifen for 2 weeks. After completion of tamoxifen treatment, the mice were randomly assigned to different treatment groups for intraperitoneal injection with αCD8α (200 μg per injection, clone: 2.43, Selleck, cat#A2102) or IgG2b (200

μg per injection, clone: LTF-2, Selleck, cat#A2116) twice a week for 5 consecutive weeks. One week later, the mice were anesthetized for micro-CT scanning, the spleens and the bronchial draining lymph nodes were collected for flow cytometry analysis, and the lungs from tumor-bearing mice were collected for subsequent histological analysis.

Hematoxylin and Eosin (HE) staining

HE staining was performed as previously(Dong et al., 2023; Wang et al., 2021; Zhang et al., 2020). Briefly, lung tissues were fixed with 2.5 mL 4% paraformaldehyde (PFA) for 4 hours followed by dehydratation in 75%, 95%, 100% ethanol successively (1 hour for each gradient). The lungs were embedded in paraffin and sectioned (5 μm) for subsequent staining with hematoxylin and eosin (Beyotime Biotech). Images were acquired using an Aperio VERSA 8 (Leica) multifunctional scanner. Tumor burden and individual tumor size were determined through ImageScope (Leica) as described previously (Dong et al., 2023; Wang et al., 2021; Zhang et al., 2020).

Multi-color immunohistochemistry (mIHC)

The experiments were performed with an Opal 5-color Manual IHC Kit (Absin, Cat #: abs50013) following the manufacturer's instructions. The slides were deparaffinized in xylene and successively rehydrated in 100%, 95%, and 75% ethanol. The antigen retrieval was performed by heating slides in a microwave for 30 minutes in sodium citrate buffer (pH 6.0). The sections were cooled down naturally to room temperature and quenched in 3% hydrogen peroxide to block endogenous peroxidase activity followed by blocking in 10% horse serum

in 1×PBS at room temperature for 15 min. The sections were treated with Click-iT™ TUNEL Colorimetric IHC Detection Kit (Invitrogen, Cat# C10625) according to the manufacturer's instructions and stained with the following antibodies: rabbit anti-mouse EpCAM (Abclonal, Cat #: A19301, 1:400) at room temperature for 1 h, and rabbit derived 4-HNE (Abcam, Cat #: ab46545, 1:500) at 4℃ overnight. A secondary horseradish peroxidase-conjugated antibody (Absin, cross-react with mouse/rabbit) was added and incubated at room temperature for 15 min. Signal amplification was performed using TSA working solution diluted at 1:100 in 1× amplification diluent (Absin) and incubated at room temperature for 15 min. After each cycle of staining, heat-induced epitope retrieval was performed to remove all the antibodies including primary antibodies and secondary antibodies. The samples were counterstained for nuclei with DAPI for 10 min after all the antigens above have been labelled and mounted in mounting medium. The multispectral images were scanned by ZEISS AXIOSCAN7 at 20× magnification and were analyzed with SliderViewer (Version 2.5).

Micro-CT scanning

Tumor-burdened mice were anesthetized under 1.0%–1.5% isoflurane via a respiratory mask of the inhalation anesthesia machine and scanned by a NEMO@ Micro-CT system (NMC200, PINGSENG TECHNOLOGY, China) to assess the lung tumor burdens. A pneumatic pillow was positioned on the thoraxes of tumor-burdened mice and connected to a pressure transducer to monitor respiratory motion and inform prospective gating. Reconstruction was performed using Avatat3 software with a 'CT-gating' strategy and an 'interaction' algorithm. Other parameters including the resolution of CT is 1k×1k, the number of iterations is 6, and

the gated phase is 8, unless indicated otherwise, reminding parameters were set with default parameters according to the manufacturer's instructions. The micro-CT images were analyzed by Avatar3 and saved in tiff format.

Quantitative Real-Time PCR

These experiments were performed as previously described (Wang et al., 2021; Yang et al., 2022). Total RNA was extracted from cells using TRIzol reagent (Takara), and the first-strand cDNA was reverse-transcribed with All-in-One cDNA Synthesis SuperMix (Aidlab, Cat: 342123AH). Gene expression was examined with a Bio-Rad CFX Connect system by a fast two-step amplification program with 2×SYBR Green Fast qPCR Master Mix (Aidlab, Cat: 342123AX). The expression levels of genes were normalized to that of the gene encoding β-actin. Gene-specific primers used in this study are summarized in Table S5.

Isolation of tumor interstitial fluid (TIF)

We used a previously reported method to isolate tumor tissue interstitial fluid via a centrifugation method(Chen et al., 2022; Eil et al., 2016). Briefly, tumor-bearing lungs were harvested and washed in pre-cold saline for three times, and drying the tumors via blotting paper after the saline rinse. Then the tumors were placed on 70 μm nylon mesh in a 50 mL Falcon tube and spun at < 50g for 5 min to remove surface liquid. Next, samples were centrifuged at 400g for an additional 10 min at 4 ℃ and carefully aspirate the supernatant for interstitial fluid collection.

Triacylglycerol (TAG) measurement

Triacylglycerol levels in supernatants from cultured cells or tumor interstitial fluid (TIF) from lung tumors were quantified using the Triglyceride-Glo^{TM} assay kit (Promega, Cat #J3160) according to the manufacturer's instructions. Briefly, primary CD45$^-$CD31$^-$EpCAM$^+$ tumor cells of KL and KLG4$^{m/m}$ were sorted and washed with cold PBS. These cells were then cultured in a 24-well plate at a density of 5×10^6 cells per well, using 300 μL DMEM supplemented 10% delipidated FBS (v/v, kindly provided by Dr. Yan Wang, Wuhan University). At the indicated time points (0, 6, and 12 h) after initiating the culture, the supernatants were collected. A 25 μL aliquot of the supernatant was transferred to a 96-well plate containing 25 μL Glycerol Lysis Solution with lipase. This mixture was gently shaken and incubated for 30 min at 37 °C. Following this, 50 μL glycerol detection mix was added to each well and incubated at room temperature for 1 h. Luminescence was measured using a plate-reading luminometer, and glycerol concentrations were calculated. For TAG measurement in TIF, the TIF was collected as described above and subsequently diluted and quantified according to the manufacturer's instructions..

Immunoblot assays

The immunoblot assays were performed as described previously (Wang et al., 2021; Yang et al., 2022). In brief, cells were lyzed and the normal tissue or tumor tissues were homogenized with NP-40 lysis buffer (150 mM NaCl, 1 mM EDTA, 1% nonidet P-40) supplemented with proteinase and phosphatase inhibitors (Biotool). The cell lysates or tissue homogenates were cleared by centrifuge at 15000 g for 10 min at 4°C. The supernatants were quantified and

loaded to 12% sodium dodecyl sulfate-polyacrylamide (SDS-PAGE) gel for electrophoresis followed by transfer onto nitrocellulose membranes. Blocking was performed in 5% skim milk (w/v) in PBS for 40 min at room temperature, and the membranes were incubated with primary antibodies for overnight at 4℃ and followed by TBST wash every 10 minutes for three times. Subsequently, the membranes were incubated with horseradish peroxidase (HRP)-conjugated secondary antibodies for 1 h followed by incubation with the enhanced chemiluminescence kit (Bio-rad, Cat# 1705061) for analysis. The primary and secondary antibodies used in this study were listed in Table S6.

Preparation of single-cell suspensions from lung tumors

The freshly tumor-burdened lungs from KL/KLG4$^{m/m}$ mice with or without indicated treatments, as well as KL/KLG4^{OE} mice were perfused through alveolar lavage and cardiac lavage with cold PBS. The tumor-burdened lungs or lung tumors were isolated and cut into small pieces (1~2 mm in diameter) followed by digesting into single-cell suspensions by Tumor Dissociation Kit (Miltenyi Biotech, Cat# 130-096-730) according to the manufacturer's instructions. Briefly, total small pieces were transferred into a gentleMACS C Tube with the enzyme mix containing 2.35 mL of DMEM, 100 μL of Enzyme D, 50 μL of Enzyme R, and 12.5 μL of Enzyme A. The C Tube was tightly closed and attached to the sleeve of the gentleMACS™ Octo Dissociator (Miltenyi Biotech) with the tumor isolation program. After termination of the program, the C tube was detached from the Dissociator and incubated at 37°C shaker with continuous rotation at 220 rpm for 40 min. Subsequently, repeat the tumor isolation program twice and perform a short spin up to 1,500 rpm to collect

the sample at the bottom of the tube. The dissociated cells were filtered through a 70 μm cell strainer and centrifuged at 3,000 g for 5 min to remove the supernatant, and then the pellets were resuspended in red blood cell lysis buffer. Cells were recovered by adding an equal volume of 1×PBS and centrifugation at 3,000 g to remove the supernatant. Finally, cells were resuspended in PBS containing 1% FBS (v/v) and used for further analysis.

Preparation of tumor-infiltrated lymphocytes (TILs)

The obtained single-cell suspensions of tumor-burdened lungs were centrifuged at 1,500 g for 5 min at room temperature, and the precipitants were re-suspended with 37% Percoll (Cat#17-0891-09, GE Healthcare) in 1×PBS (v/v). The suspension was centrifuged at 3,000 g for 30 min at 4°C and the supernatant was discarded. The precipitants containing TILs were re-suspended in 1×PBS containing 1% FBS and used for further analysis.

Cell sorting

The single-cell suspensions of tumor-burdened lungs or spleen were incubated with CD16/32 antibody for 20 min at 4°C before surface staining with fluorescence-conjugated antibodies for 30 min at 4°C. Information of antibodies used in fluorescence-activated cell sorting (FACS) was listed in Table S6. The stromal cells ($CD45.2^-CD31^-CD49d^+$), endothelial cells ($CD45.2^-CD31^+$) or tumor cells ($CD45.2^-EpCAM^+CD31^-$) were separated with a BD FACSAria II cell sorter (BD Biosciences) (> 90% purity). When necessary, the cells were isolated with CD45 magnetic Nanobeads sorting (Biolegend, Cat #:480028), Dynabeads™ FlowComp™ Mouse CD8 Kit (Invitrogen, Cat# 11462D), Dynabeads™ FlowComp™ Mouse

CD4 Kit (Invitrogen, Cat# 11461D) or MojoSort™ Mouse CD326 (EpCAM) Selection Kit (Biolegend, Cat# 480142) prior to cellular surface staining according to the manufacturer's instructions.

Single-cell suspensions from spleens of OT-I mice were prepared by mechanical disruption in ice-cold 1×PBS. Cells in suspension were centrifuged for 5 min at 1,500 g. After red blood cell lysis, cells were filtrated through a 70 μm nylon mesh. Splenic $CD8^+$ T cells or intratumoral $CD8^+$ T cells of lung tumors were isolated using a Dynabeads™ FlowComp™ Mouse CD8 Kit (Invitrogen, Cat# 11462D) according to the manufacturer's instructions.

Syngeneic graft mouse model and treatment

In the syngeneic graft mouse models, $CD45^-CD31^-EpCAM^+$ tumor cells were sorted from lung tumors of $KL/KLG4^{m/m}$ mice that were injected with Ad-Cre followed by tamoxifen treatment. The tumor cells were cultured in DMEM supplemented with 10% FBS (v/v, Gibco) in the presence of Lip-1 (25 μM, Selleck, Cat #S7699), and the medium was changed every other day until cells outgrew and stable cell lines were formed. The cells were harvested and injected in the left back flank of C57BL/6 mice (5×10^6/mouse, s.c.), the tumor length (L) and width (W) were measured and the tumor size was calculated as the formula: $0.5\times L\times W^2$. Once the tumor volume reaches approximately to 1,500 mm^3, the mice were euthanized and the tumors were harvested for various analyses.

To inducible knockout of GPX4 in syngeneic graft mouse models, primary $CD45^-CD31^-EpCAM^+$ tumor cells were sorted from lung tumors of $KL/KLG4^{m/m}$ mice (without tamoxifen treatment) after 8 weeks of Ad-Cre injection and subcutaneous transplanted into the left flanks

of wild-type C57BL/6 mice. When the tumors became palpable, the mice were assigned randomly into different treatment groups. Tamoxifen was intraperitoneally injected daily at a dosage of 80 mg per kg body weight (dissolved in corn oil) and continued for 5 days and followed by intraperitoneally injected with Lip-1 (10 mg/kg) or control (as described below) once a day for 15 successive days. The tumor length (L) and width (W) were measured and the tumor size was calculated as the formula: $0.5 \times L \times W^2$. Once the tumor volume approximates to 1500 mm^3, the tumors were harvested and prepared for various analyses.

Cell staining and flow cytometry analysis

The antibodies and reagents used for flow cytometry staining are summarized in Table S6. Flow cytometry protocol has been previously described (Dong et al., 2023; Zhang et al., 2020). In short, the single-cell suspensions of tumor-burdened lungs or the obtained TILs were re-suspended in 1×PBS containing 1% FBS (v/v) and blocked with anti-mouse CD16/32 antibodies for 20 min prior to staining with the antibody mixture. Surface staining was performed in PBS containing 1% FBS (v/v) at 4°C for 30 min. For intracellular cytokine staining, cells were fixed and permeabilized with a fixation and permeabilization solution kit (Cat#424401 and 421002, respectively, Biolegend) according to the manufacturer's instructions followed by staining with the specific antibodies against intracellular markers. For the detection of cytokine production, TILs were stimulated for 4 hours at the 37°C incubator (5% CO_2) in the presence of PMA (50 ng/mL, Cat#P8139, Sigma), Ionomycin (500 ng/mL, Cat#I0634, Sigma), and Golgi-stop (1:1000, Cat#554724, BD Biosciences), followed by intracellular staining. Staining of TCF1 and TOX was performed with the True-Nuclear

Transcription Factor Staining Buffer Set (Cat#424401, Biolegend) according to the manufacturer's instructions. Subsequently, cells were fixed in 4% paraformaldehyde for 15 min at 4 °C, and then centrifuged at 3,000 g for 5 min at 4°C and the supernatant was discarded. The bottom cells were re-suspended in 1×PBS containing 1% FBS and used for flow cytometry analysis. Flow cytometry data were acquired on a FACSCelesta or LSRFortessaX20 flow cytometer (BD Biosciences) and analyzed by using FlowJo (v10.8.1) software (Tree Star).

Lipid peroxidation and cell viability measurement

Experiments were performed according to the manufacturer's protocol. Briefly, single-cell suspensions of lung tumors were cultured in blank RPMI-1640 growth medium containing 30 nM SYTOX Green Dead Cell Stain (Invitrogen) or 5 μM C11-BODIPY (lipid peroxidation sensor, Invitrogen), and incubated for 30 min at 37°C in a cell culture incubator before surface markers staining. Cells were centrifuged, washed, and resuspended in 200 μL fresh PBS followed by flow cytometry analysis within 2 hours of staining.

Transmission electron microscopy (TEM) of cells

$CD45^-CD31^-EpCAM^+$ tumor cells with or without iDGAT1/2 treatment were sorted as described above. After washing for three times with ice-cold PBS, the cells were lifted and pelleted at progressively increasing g forces (1,000 g for 5 min, 3,000 g for 5 min, 6,000 g for 5 min). The cell pellets were fixed in 2.5% electron microscopy grade glutaraldehyde in PBS at 4°C for 2 hours, postfixed in 2% aqueous osmium tetraoxide, dehydrated in gradual ethanol

(30-100%), propylene oxide (two cycles at 4℃ for 5 minutes) followed by infiltrated sequentially in 1:1 (v:v) propylene oxide/epoxy resin for 4 h, 1:2 (v:v) propylene oxide/epoxy resin (overnight), 100% fresh epoxy resin for 4 h. Finally, the cell pellets were embedded in 100% fresh epoxy resin and cured for 48 h at 65℃. Ultrathin sections of 50 nm were collected onto 200 mesh copper grids, and stained with Sato lead Sodium citrate for 1 min and observed using a JEM-1400 plus electron microscope operated at 100kV. For analysis, mitochondria and lipid droplets were identified by a combination of manual and automatic segmentation. The area of each mitochondria and lipid droplet identified was calculated using ImageJ v.1.52a (Bethesda) based on pixel sizes during TEM image acquisition.

Oil red O staining

$CD45^{-}CD31^{-}EpCAM^{+}$ tumor cells were isolated from lung tumors of KL and $KLG4^{m/m}$ mice as previously described. The cells were washed twice with ice-cold PBS and then fixed in 4% formalin at room temperature for 15 minutes. After discarding the 4% formalin, the cells were washed with 60% isopropyl alcohol and dried at room temperature. Next, the cells were stained with 5% Oil Red O reagent (Beyotime, Cat#C0157M) for 10 min.

Imaging was performed using a LEICA microscope equipped with a 10×objective, and images were captured with a HAYEAR 8MP USB3.0 CMOS Video Electronic Microscope Camera (Shenzhen Hayear Electronics Co. Ltd.) using the S-EYE imaging software (Shenzhen Hayear Electronics Co. Ltd.). Three independent fields were acquired for each experimental condition, and representative images from one field of view are presented. After imaging, the Oil Red O solution was discarded, and the cells were dried at 37℃ for 1 hour.

The staining was quantified by extracting the Oil Red O stain with 100% isopropyl alcohol, and the absorbance was measured using a spectrophotometer at 510 nm.

Immunofluorescence and confocal microscopy analysis

$CD45^{-}CD31^{-}EpCAM^{+}$ tumor cells from lung tumors of $KL/KLG4^{m/m}$ or $KL/KLG4^{OE/+}$ were sorted as described above and washed with PBS three times. After that, the cells were incubated with 2 μM C11-bodipy solution in the dark for 30 min at 37 ℃ cell incubator and washed with PBS three times. Followed by fixing in 4% paraformaldehyde (PFA) for 15 min and washed with PBS three times. For APOE staining, the C11-bodipy labelling and 4% PFA fixed cells were permeabilized with 0.5% saponin in PBS for 5 min on ice and washed with PBS three times. Then, the cells were blocked in 1% BSA containing PBS (v/v) for 1 h and stained in blocking buffer with primary antibody overnight at 4℃. The cells were further stained with 594-conjugated secondary antibody for 1 h at 4℃. Finially, the cells were plated on slides and stained with In Situ Microplate Nuclear Stain and AntiFade (Sigma, Cat# DUO82064-1KIT), and the coverslips were mounted on slides. Images were acquired on an Olympus FV1000 fluorescence microscope.

Delipid of the culture supernatants of tumor cells

We used a density-gradient ultracentrifugation method to remove the majority of lipoprotein classes from the culture supernatants of $CD45^{-}CD31^{-}EpCAM^{+}$ tumor cells, derived from KL and $KLG4^{m/m}$ tumors (Chapman et al., 1981). In brief, the density of the culture supernatants was increased to 1.3 g/mL through the addition of solid potassium bromide. After

constructing the gradient, we centrifuged them at 27500 rpm for 24 hours at 4℃, without using a brake at the end of the run. Following this, we carefully aspirated the upper phase, which contained the lipoprotein, and transferred the lower aqueous phase to a sterile dialysis tubing with a molecular weight cutoff of 1000. Next, we immersed the dialysis tubing in 0.9% NaCl for 12 hours at 4℃, a process that was repeated four times. Finally, the lipid-free supernatants were filtered through a 0.22 μm filter and stored at -20℃, ready for use in *in vitro* cell stimulation experiments.

***In vitro* chronic and acute stimulation of P14 $CD8^+$ T cells with tumor cells-derived supernatants**

$CD8^+$ T cells were isolated from spleens of P14 transgenic mice (carrying a transgenic T cell antigen receptor that recognizes H-2$D^b$$GP_{33-41}$ epitope of LCMV, which were kindly provided by Prof. Liang Cheng, Wuhan University) and T-cell-depleted APCs were isolated from spleens of wild-type C57BL/6 mice and pulsed with 250 nM GP33-41 (KAVYNFATM, Synthesized by GL Biochem, Shanghai) in RPMI-1640 for 4 h at 37℃ followed by fixation in 4% paraformaldehyde and twice wash in PBS. The fixed APCs were cocultured with $CD8^+$ P14 cells at a 2:1 ratio (2×10^6 APCs + 1×10^6 P14 cells per well) in a flat-bottom 24-well plate. For chronic stimulation of T cells, at d2, d4, and d6 of coculture, $CD8^+$ P14 cells were harvested from a 24-well plate and isolated via a $CD8^+$ positive selection kit (Invitrogen, Cat: 11462D), replated into a 24-well plate (1×10^6 P14 cells per well) and chronically stimulated by coculture with 50 nM GP_{33-41} pulsed APCs. Both the acute and chronic *in vitro* conditions received recombinant mouse IL-2 (10 ng/mL, Cat: CM003-20MP, CHAMOT). On day 2,

cells were cultured with either tumor cells supernatants or their delipidated counterparts from KL or KLG4$^{m/m}$ tumor cells (100 μL/well of KL and KLG4$^{m/m}$, respectively). Finally, the cells were harvested and analyzed through flow cytometry on BD LSRFortessaX20.

Targeted oxi-lipidomic profiling and data analysis

The experimental procedure followed the methodology outlined in previous studies(Li et al., 2024; Yin et al., 2009). Tumor cells ($CD45^{-}CD31^{-}EpCAM^{+}$) from lung tumors of KL and KLG4$^{m/m}$ with or without indicated treatments were sorted as previoudly detailed. Alternatively, the tumor cells culture supernatants were lyophilized prior to analysis. For each sample, 5×10^{6} cells were collected and washed once with ice-cold PBS, after centrifuging for 5 min at 1, 000 rpm, cell pellets were resuspended in 140 μL ice-cold nuclease-free water and vortexed for 10 s, added 100ng phosphatidylethanolamine (PE) (D16:1) and 100ng phosphatidylcholine (PC) (D14:1) as internal standard. Lipids were extracted using the Folch method. Briefly, 450 μL ice-cold chloroform/methanol (v/v = 2:1) containing 0.005% BHT was added to each sample, vortexed for 1 min and the sample was then incubated on ice for 15 min to enhance extraction efficiency. Finally, samples were centrifuged for 10 min at 3, 000 g, 4 °C. The organic layers (lower) were collected into new tube and dried under nitrogen using a 12-port drying manifold. Dried samples were resuspended in 60 μL of 100% LC solvent B (methanol /isopropanol solution, v/v, 3:4, containing 5 mM amide acetate). A 50 μL aliquot of the sample was transferred to a new autosampler vial for analysis.

Chromatography was performed using a HILIC HPLC column (Luna 5μm, 100 Å, 50 × 2 mm, Phenomenex) at a flow rate of 0.350mL/ min. Mass spectrometric analysis was

performed in the negative ion mode using multiple-reaction monitoring (MRM) of specific precursor–product ion m/z transitions upon collision-induced dissociation. The precursor negative ions monitored were the molecular ions $[M-H]^-$ for PE, and the acetate adducts $[M+CH3COO]^-$ for PC. Identity was further verified by monitoring at the same time, using polarity switching, the positive molecular ions $[M+H]^+$ for both PC and PE molecular species. The product ions analyzed after collision-induced decomposition, and used for data comparison, were the carboxylate anions corresponding to the non-oxidized or oxidized arachidonoyl chains. The specific precursor–product pairs monitored in negative-ion mode and used for quantification were follows: PE(16:0e_22:5(O)), 766/345; PE(18:1a_22:4(O)), 808/347; PC (18:0a_20:4(2O)), 900/335; PC(18:0a_22:4(2O)),928/363; PE(18:0a_9'-oxo-nonanoyl), 634/171 ; PE(18:0a_5'-oxo-valeroyl), 578/115 ; PE(16:0a_22:4(O)), 782/347; PC(18:0a_HETE), 810/319(Doll et al., 2019; Friedmann Angeli et al., 2014; Li et al., 2024). Calculated oxygenated lipids were summarized in Table S1.

Lipidomic profiling and data analyses

Lipidomics procedures. $CD45^-CD31^-EpCAM^+$ tumor cells and their culture supernatants of KL and $KLG4^{m/m}$ with or without indicated treatments were obtained as described above. Briefly, the cells were washed with ice-cold PBS twice, for each sample, 5×10^6 tumor cells were collected and stored at -80°C. Cell pellets were resuspended in 300 µl methanol/water (v/v=3:1), containing of LPC 18:1(d7) as the internal standard. Then, each sample was sonicated for 30 s to ensure homogeneity and added with 750 µl MTBE, vortexed for 60 s, and gently vibrated for another 30 min. After that, the sample was added with 190 µl of nuclease-

free water and vortexed for 1 min. After equilibration at room temperature for 10 min, the sample was centrifuged at 14,000g for 15 min. Then 400 μl aliquot of the upper lipid extract was pipetted into the new centrifuge tube and vacuum-dried. The dried sample was dissolved with 150 μl of acetonitrile/isopropanol/water (v/v/v = 65:30:5) for the instrumental analysis in the positive and negative ion mode. The lipid profiling was acquired by using a liquid chromatography-mass spectrometry (LC-MS) system comprising ultra-performance liquid chromatography (UPLC, ExionLC™ AD, https://sciex.com.cn/) coupled to a Tandem Mass Spectrometry (MS/MS, QTRAP®6500+, https://sciex.com.cn/). Dissolved samples were centrifuged at 12,000 rpm for 10 min to remove residual cellular debris before injecting 2 μL onto a Thermo Accucore™ C30 column (100×2.1 mm i.d., 2.6 μm). Parameters on the chromatographic separation in the positive ion mode were the same as those in the negative ion mode. The column was eluted at 80% mobile phase A (60:40 v/v acetonitrile/water, containing 0.1% formic acid and 10 mM ammonium formate) and 20% mobile phase B (10:90 v/v acetonitrile/ isopropanol, containing 0.1% formic acid and 10 mM ammonium formate) between 0 and 2 min, raised to 30% B at 2 min, increased linearly from 30% to 95% B between 2 and 17.3 min, returned to 20% B at 17.5 min, and held at 20% B until 20 min. The flow rate was 0.35 mL/min from 0 to 20 min at 45°C. MS data were acquired using electrospray ionization (ESI) at 500°C, The spray voltage was 5.5 kV and 4.5 kV for positive and negative ionization modes, respectively. Sheath, auxiliary, and sweep gases were 45, 55 and 35 psi, respectively. Data was collected in full MS/dd-MS2 (top 5). Full MS was acquired from 100-1500 m/z with a resolution of 70,000, AGC target of 1×10^6 and a maximum injection time of 100 ms. MS2 was acquired with resolution of 17,500, a fixed first mass of

50 m/z, AGC target of 1×10^5 and a maximum injection time of 200 ms. Stepped normalized collision energies were 20, 30 and 40%.

Lipidomics data processing. Raw data were processed using Analyst 1.6.3 (SCIEX) software for the detection and integration of LC-MS peaks. Lipid identities were determined based on a homemade database-MWDB (metware database) according to the retention time (RT) and pairs of precursor- and product-ion of lipids to be tested and were denoted by total number of carbons in the lipid acyl chain(s) and total number of double bonds in the lipid acyl chain(s). Lipids quantitative analysis was performed on multiple reaction monitoring (MRM) to obtain the area of LC-MS peaks in a low noise background. Peak areas were used in data reporting, data was normalized using internal standards. Negative ion mode analyses of free fatty acids and bile acids (C18-neg) were conducted similarly to that previously described (Paynter et al., 2018). The abundance (nmol/g) of each lipid species was calculated by the following formula: $X = 0.001\times R\times c\times F\times V/m$, where X (nmol/g) is the concentration of the lipids, R is the ratio of peak areas of lipids to be tested related to internal standards, c is the concentration (μmol/L) of internal standards, F is the correlation factor of different internal standards, and V and m are the total volumes (μL) of the lipid extracts and weight of tumor tissues of each sample, respectively. Differential-abundance analysis was performed using 'DEP' R package (v 1.26.0). Calculated lipidomics datasets were summarized in Table S1.

Bulk RNA Sequencing

RNA-sequencing and high throughput sequencing were conducted by Seqhealth

Technology Co., LTD (Wuhan, China). Intratumoral $CD8^+$ T cells were isolated by MACS and tumor-infiltrated tumor cells were sorted by FACS as described above, respectively. And immediately homogenized in 2 mL of TRIzol (Takara), and total RNA was extracted using RNeasy Kit (QIAGEN). RNA concentration and purity were measured by using NanoDrop 2000 (Thermo Fisher Scientific, Wilmington, DE) and RNA integrity was assessed using the RNA Nano 6000 Assay Kit of the Agilent Bioanalyzer 2100 system (Agilent Technologies, CA, USA). Poly(A) mRNA was subsequently purified from 10μg total RNA using NEBNext Oligo $d(T)_{25}$ Magnetic Beads Isolation Module. First-strand complementary DNA was synthesized with NEBNext RNA First-Strand Synthesis Module. NEBNext Ultra II Non-Directional RNA Second Strand Synthesis Module was used for the synthesis of the complementary strand of first-strand cDNA. The resulting double-stranded DNA was purified and Vazyme TruePrep DNA Library Prep kit V2 was used to prepare libraries followed by sequencing on an Illumina Hiseq X platform with 150-bp paired-end reads strategy. Quality control of mRNA-seq data was performed by using Fatsqc (v0.11.9) and low-quality bases were trimmed by Trim_galore (0.6.4_dev). All RNA-seq data were mapped to the mouse genome (Mus_musculus_Ensemble_94) by Hisat2 (v.2.0.5) and allowed a maximum of two mismatches per read. Gene expression level was calculated by FeatureCounts (v.2.0.0) with default parameters and normalized by FPKM (Fragments Per Kilobase of exon model per Million mapped fragments). Differential expression analysis of two groups was performed using the DESeq2. Based on these differentially expressed genes, Gene Ontology (GO) and Kyoto Encyclopedia of Genes and Genomes (KEGG) enrichment analyses were implemented by using the clusterProfiler(Wu et al., 2021b) (v4.8.2) R package.

To analyze a positive or negative enrichment of the indicated pathways, we performed gene-set enrichment analyses (GSEA) with the mRNA-seq data as previously described (Dong et al., 2023; Wang et al., 2021; Yang et al., 2022). In such analyses, the gene sets annotated in MSigDB (Molecular Signatures Database, https://www.gsea-msigdb.org/gsea/msigdb) were listed in Table S4, and their corresponding expression values (Table S2) were analyzed through the GSEA software (http://www.gsea-msigdb.org/gsea/index.jsp).(Subramanian et al., 2005)

CUT & Tag sequencing and data analysis

CUT&Tag experiment and high throughput sequencing were conducted by Seqhealth Technology Co., LTD (Wuhan, China). Briefly, $CD45^{-}CD31^{-}EpCAM^{+}$ tumor cells were isolated from lung tumors of KL and $KLG4^{m/m}$ mice as previoudly detailed. 1×10^{6} cells from each mouse (three mice in total) were harvested and following the manufacturer's protocol of the CUT&Tag kit (Cell Signaling Technology, Cat#86652). the cells were permeabilized with digitonin and incubated with anti-H4K4me3 (Abcam, Cat# 8580) or anti-H3K27ac(Abcam, Cat# ab4729) (1:50 dilution for all antibodies) for 2 hours at 4°C. Permeabilized cells were washed and incubated with pAG-MNase enzyme, comprising the IgG-binding domain of protein A/G fused to micrococcal nuclease, for 1 hour at 4°C. MNase activity was dependent on Ca^{2+}, and remianed inactive before the addition of $CaCl_2$. One hour later, the permeabilized cells were washed and incubated with cold $CaCl_2$ for 30 minuates at 4°C to activate MNase, and to cleave and liberate the chromatin fragments bound by H3K4me3 and H3K27ac. The chromatin digestion reaction was then stopped, and chromatin fragments were purified with

DNA purification spin columns. We performed PCR to amplify the chromatin fragments and generated libraries with the SimpleChIP ChIP-seq DNA Library Prep Kit (Cell Signaling Technology), which was compatible with the CUT&Tag kit from the same company. The library products were enriched, quantified and finally sequenced on a DNBSEQ-17 sequencer (MGI Tech Co.,Ltd.China) with PE150 model.

Quality control of sequencing data was performed by using Fatsqc (v0.12.1) and low-quality bases were trimmed by Trim_galore (v0.6.10). The trimmed reads were mapped to the mouse genome (Mus_musculus_Ensemble_94) by BWA (v0.7.15) with default parameters. Reads that mapped to mitochondrial DNA or that had low mapping quality (<30) were excluded from downstream analysis. Next, Duplicate reads due to PCR amplification of single DNA fragments during library preparation were identified with Picard (v 2.17.3; available at http://broadinstitute.github.io/picard) and removed from downstream analysis. MACS2 (v 2.2.7.1) was used for transcription factor binding signal peak calling and the HOMER software suite (v 4.11.1) was used to assign identified peaks to genes. Multiple peaks could be assigned to a single gene as long as the peaks were located in the gene body or its promoter region (within 2.5 kb upstream). Finally, Integrative Genomics Viewer (IGV, v2.5.0) was used to display the signals of CUT&Tag sequencing.

Public datasets mining

Single-cell RNA sequencing (scRNA-seq) data were obtained from the GEO dataset GSE207422(Hu et al., 2023) (human NSCLC tissues) and GSE165641(Wang et al., 2021) (KL NSCLC mouse model). Cells annotated as “tumor cells” (specifically expressed *EPCAM*,

KRT8, and *KRT19*) in the original metadata were extracted for downstream analysis. Based on the distribution of *GPX4* expression levels, the top 25% of tumor cells were defined as the *GPX4*^{High} group, and the bottom 25% as the *GPX4*^{Low} group. The mRNA levels of *DGAT2*, *APOE*, and *GPD1L* were compared between the two groups. Statistical significance was determined using a two-tailed Student's *t*-test. Data visualization was performed using the ggplot2 package in R (version 4.3.2).

For correlation analysis between the mean *GPX4* expression in tumor cells and the exhaustion score of T cells across patients in the datasets of GSE148071(Wu et al., 2021a), HRA002509(Hu et al., 2023), HRA001033(Yan et al., 2025), and GSE136246(Maroni et al., 2021), respectively. For each patient from the above dataset, tumor cells were identified based on the cell type annotations provided in the original dataset. The mean *GPX4* expression level within tumor cells of each patient was calculated. To quantify the degree of $CD8^+$ T cell exhaustion from the same patients, we used the AddModuleScore function in the Seurat package (version 5.1.0) to compute exhaustion scores for T cells. The T cell exhaustion gene signature included the following genes: *PDCD1*, *HAVCR2*, *CTLA4*, *LAG3*, *TIGIT*, *ENTPD1*, *NT5E*, *BATF*, *NFATC1*, *CD38*, *EOMES*, *SLAMF6*, and *TOX*. Data visualization was performed using the ggplot2 package in R (version 4.3.2). And the relationship between the mean *GPX4* expression in tumor cells and the corresponding T cell exhaustion score across patients was evaluated using Pearson correlation analysis.

Chromatin Immunoprecipitation (ChIP) assay

The tumor cells ($CD45^-CD31^-EpCAM^+$) from the autochthonous and syngeneic $KL/KLG4^{m/m}$

tumors were sorted as described above. The tumor cells were fixed with DSG (2 mM) for 30 min and 1% formaldehyde for 15 min, and then quenched by glycine. After washing three times with PBS, they were harvested in ChIP lysis buffer (50 mM Tris·HCl, pH 8.0, 0.5% SDS, 5 mM EDTA) followed by sonication to generate DNA fragments of 250-1000 bp. The lysate was centrifuged at 12,000 rpm for 10 min at 4 °C and was diluted with ChIP dilution buffer (20 mM Tris·HCl, pH 8.0, 150 mM NaCl, 2 mM EDTA, 1% Triton X-100) (4:1 volume). The resulting lysate was then incubated with Protein G agarose and anti-H3K4me3 (Cell Signaling Technology, Cat# 9751), anti-H3K27ac (Cell Signaling Technology, Cat# 8173) or control IgG at 4 °C overnight. DNA was eluted using ChIP elution buffer (0.1 M NaHCO3, 1% SDS, 30 μg/mL proteinase K) by incubation at 65°C overnight, and the DNA was purified with a DNA purification kit (TIANGEN, DP209-03). The purified DNA was assayed by quantitative PCR using the SFX connect system by a fast two-step amplification program with 2×SYBR Green Fast qPCR Master Mix (Aidlab, Cat: 342123AX). The qPCR primer for the *Dgat2* and *Gpd1l* promoter were designed according to CUT&Tag results and sequences were listed in Table S5.

Statistical analysis

Statistical analysis was performed with GraphPad Prism 8.3.0 software by two-tailed Student's *t*-test and one-way ANOVA unless indicated otherwise, two-way ANOVA was performed for multiple comparisons, the log-rank (Mantel-Cox) test was performed for comparing mouse survival curves. n represents the number of mice or samples used in the experiment, with the number of individual experiments listed in the legend and no statistical methods were used to predetermine sample size. Experiments in the study were repeated at

least two times. Graphs show individual samples and center values indicate the mean. P values < 0.05 were considered significant (*:$P < 0.05$; **:$P < 0.01$; ***$P < 0.001$, ****$P < 0.0001$; ns stands not significant, P values > 0.05). Graphs show mean ± SEM of different mice or samples unless indicated otherwise. A hypergeometric test was performed for Gene pathway enrichment analysis. GSEA was used to rank the probes and analyze the enrichment based on t statistics (http://www.broadinstitute.org/gsea/). The heatmap of the signature genes was generated in GraphPad Prism 8.3.0 software based on their relative expression levels, which were normalized to their mean and then divided by their variance.

Figure S1

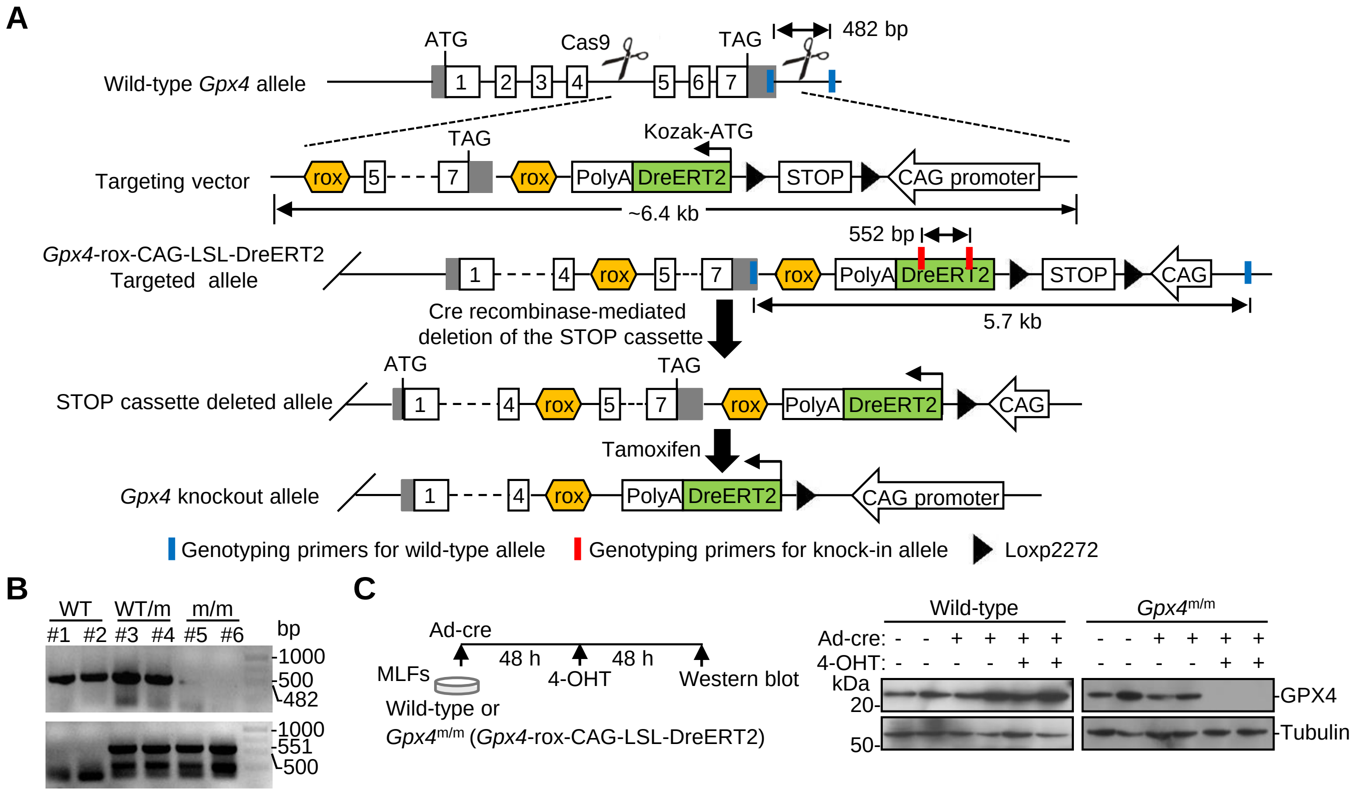

Figure S1 The generation of *Gpx4*$^{m/m}$ mice.

(A) A scheme of the generation of the *Gpx4*-rox-CAG-LSL-DreERT2 (*Gpx4*$^{m/m}$) mice.

(B) PCR analysis of the tail genomic DNAs from wild-type, *Gpx4*$^{m/+}$, *Gpx4*$^{m/m}$ mice.

(C) Experimental design (left scheme) and immunoblot (right panels) analysis of GPX4 in wild-type and *Gpx4*$^{m/m}$ MLFs infected with Ad-Cre (Ad-Cre) for 48 hours followed by treatment with 4-hydroxytamoxifen (4OHT, 1μM) for 48 hours.

Data are representative results of two independent experiments (B and C).

Figure S2

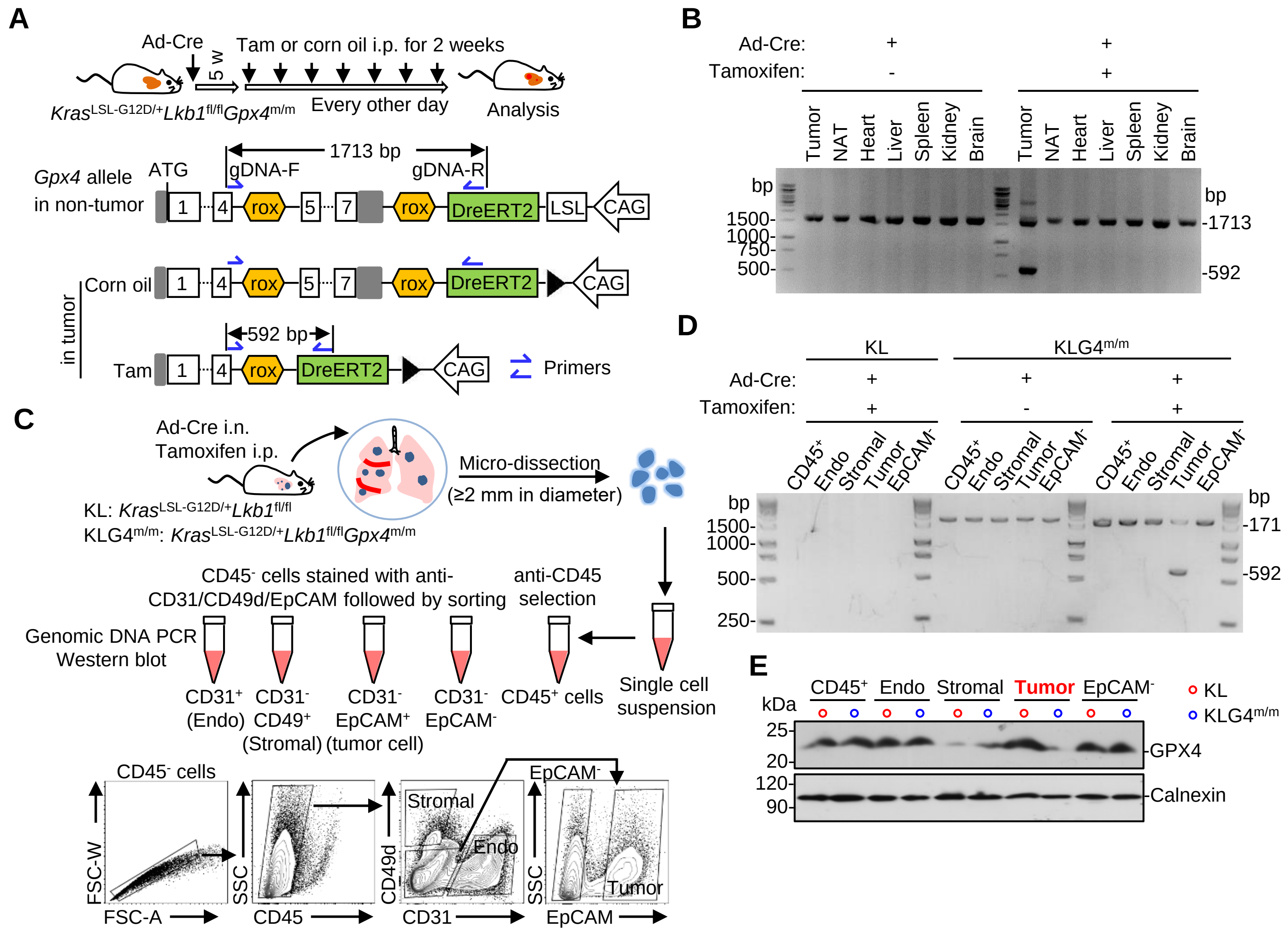

Figure S2 Tamoxifen-mediated knockout of GPX4 in tumor cells of *Kras*$^{LSL\text{-}G12D}$*Lkb1*$^{fl/fl}$*Gpx4*$^{m/m}$ (KLG4$^{m/m}$) mice.

(A) *In vivo* validation of the recombination of *Gpx4* gene locus in lung tumors of tumor-bearing *Kras*$^{LSL\text{-}G12D}$*Lkb1*$^{fl/fl}$*Gpx4*$^{m/m}$ (KLG4$^{m/m}$) mice. The KLG4$^{m/m}$ mice were intranasally injected with Ad-Cre (3$\times$10^6 PFU per mice) for 5 weeks followed by intraperitoneal injection of either tamoxifen (Tam, 80 mg/kg, resolved in corn oil) or corn oil every other day for 2 weeks for various analyzes (upper). A scheme of the *Gpx4* genomic locus in non-tumor or in Tam- or corn oil-treated tumor tissues of KLG4$^{m/m}$ mice (bottom).

(B) PCR analysis of genomic DNAs from different tissues from the Ad-Cre-infected Tam- or corn oil-treated KLG4$^{m/m}$ mice as in (A).

(C) Validation of tumor cell-specific recombinant of the *Gpx4* gene locus from lung tumors of KL and KLG4$^{m/m}$ mice treated as in (A). A scheme of magnetic-activated cell sorting (MACS) to obtain CD45$^+$ cells and fluorescence-activated cell sorting (FACS) to obtain endothelial cells, stromal cells, and tumor cells form the CD45$^-$ population (upper) and the representative gating images for cell sorting (bottom) were shown here.

(D-E) PCR (D) and immunoblot (E) analysis of the indicated cells from lung tumors of KL and KLG4$^{m/m}$ mice obtained in (C) to determine the knockout efficiency and specificity of GPX4.

Data are representative results of two independent experiments (B-E).

Figure S3

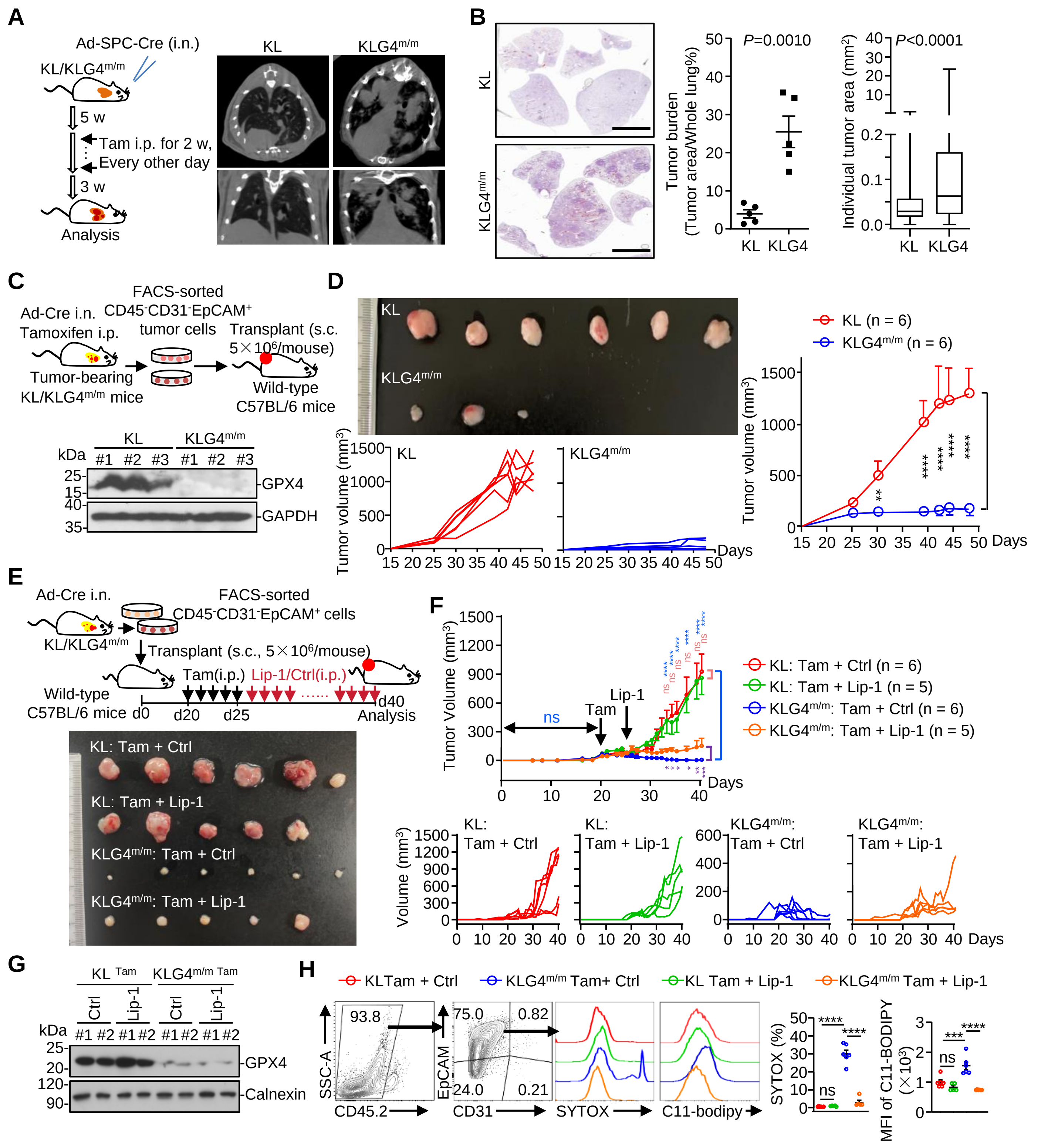

A
Ad-SPC-Cre (i.n.)
KL/KLG4m/m
5 w
Tam i.p. for 2 w,
Every other day
3 w
Analysis
KL
KLG4m/m
B
KL
KLG4m/m
P=0.0010
Tumor burden
(Tumor area/Whole lung%)
KL
KLG4
P<0.0001
Individual tumor area (mm2)
KL
KLG4
C
Ad-Cre i.n.
Tamoxifen i.p.
FACS-sorted
CD45-CD31-EpCAM+
tumor cells
Transplant (s.c.
5×106/mouse)
Tumor-bearing
KL/KLG4m/m mice
Wild-type
C57BL/6 mice
kDa
KL
KLG4m/m
#1 #2 #3 #1 #2 #3
GPX4
GAPDH
D
KL
KLG4m/m
Tumor volume (mm3)
KL (n = 6)
KLG4m/m (n = 6)
Days
E
Ad-Cre i.n.
FACS-sorted
CD45-CD31-EpCAM+ cells
KL/KLG4m/m
Transplant (s.c., 5×106/mouse)
Tam(i.p.)
Lip-1/Ctrl(i.p.)
Wild-type
C57BL/6 mice
d0
d20
d25
d40
Analysis
KL: Tam + Ctrl
KL: Tam + Lip-1
KLG4m/m: Tam + Ctrl
KLG4m/m: Tam + Lip-1
F
Tumor Volume (mm3)
ns
Tam
Lip-1
Days
KL: Tam + Ctrl (n = 6)
KL: Tam + Lip-1 (n = 5)
KLG4m/m: Tam + Ctrl (n = 6)
KLG4m/m: Tam + Lip-1 (n = 5)
Volume (mm3)
KL:
Tam + Ctrl
KL:
Tam + Lip-1
KLG4m/m:
Tam + Ctrl
KLG4m/m:
Tam + Lip-1
Days
G
KL Tam
KLG4m/m Tam
Ctrl
Lip-1
kDa
#1 #2 #1 #2 #1 #2 #1 #2
GPX4
Calnexin
H
KLTam + Ctrl
KLG4m/m Tam+ Ctrl
KL Tam + Lip-1
KLG4m/m Tam + Lip-1
93.8
SSC-A
CD45.2
75.0
0.82
24.0
0.21
EpCAM
CD31
SYTOX
C11-bodipy
SYTOX (%)
MFI of C11-BODIPY (×103)
ns

Figure S3 Tumor cell-specific deficiency of GPX4 inhibits tumor growth in syngeneic graft mouse models.

(A) KL (n = 5) and KLG4$^{m/m}$ (n = 5) mice were intranasally injected with Ad-SPC-Cre (5×10^6 PFU per mouse) for 5 weeks followed by intraperitoneal injection of tamoxifen every other day for 2 weeks (left scheme). The mice were rested for another 3 weeks followed by micro-CT imaging analysis (right images).

(B) Representative images of HE staining (left) and statistics of tumor burdens (middle) and individual tumor sizes (right) of tumor-burdened lungs from the KL (n=5) and KLG4$^{m/m}$ (n=5) mice treated as described in (A).

(C) The $CD45^-CD31^-EpCAM^+$ tumor cells were FACS-sorted from lung tumors of KL and KLG4$^{m/m}$ mice (with Ad-Cre i.n. infection followed by tamoxifen i.p. treatment) and subcutaneously transplanted into the flanks of wild-type C57BL/6 mice for further analysis (upper scheme). Immunoblot analysis of GPX4 and GAPDH in the subcutaneous KL or KLG4$^{m/m}$ tumors (lower panels).

(D) Images of the transplanted tumors as described in (C) (left top). Individual tumor growth curves (left bottom) and the overall tumor growth curves (right) of the subcutaneous KL and KLG4$^{m/m}$ (n = 6 in each group).

(E) The $CD45^-CD31^-EpCAM^+$ tumor cells were sorted from lung tumors of KL and KLG4$^{m/m}$ mice (with Ad-Cre i.n. infection only) for10 weeks and transplanted subcutaneously into the flanks of wild-type C57BL/6 mice. When the tumors became palpable, the mice were intraperitoneal injected with tamoxifen (80 mg/kg in corn oil) for 5 successive days followed by daily intraperitoneal injection of Liprostatin-1 (Lip-1, 10 mg/kg) or the control dissolvent (Ctrl) for 15 consecutive days followed by various analyses (upper scheme). The individual tumors were imaged at the end of the study (lower image).

(F) The overall tumor growth curves (top) and the individual tumor growth curves (bottom) for KL (n = 6 for Ctrl and n = 5 for Lip-1) or KLG4$^{m/m}$ (n = 6 for Ctrl and n = 5 for Lip-1) subcutaneous tumors as described in (E).

(G) Immunoblot analysis of GPX4 in the for KL (n = 2 for Ctrl and Lip-1) or KLG4$^{m/m}$ (n = 2 for Ctrl and Lip-1) subcutaneous tumors as described in (E).

(H) Flow cytometry analysis (left flow charts) and the percentages of cell death and lipid peroxidation (right graphs) of $CD45^-CD31^-EpCAM^+$ tumor cells from KL (n = 6 for Ctrl and n = 5 for Lip-1) or KLG4$^{m/m}$ (n = 6 for Ctrl and n = 5 for Lip-1) subcutaneous tumors as described in (E).

Graphs show mean ± SEM (B, D, F, and H). *P<0.05, **P<0.01, *** P < 0.001, **** P <0.0001, ns: not significant (two-tailed student's *t*-test for B or two-way ANOVA for D, F, and H). Scale bars represent 5 mm (A and B).Data are representative results of two independent experiments (A-H).

Figure S4

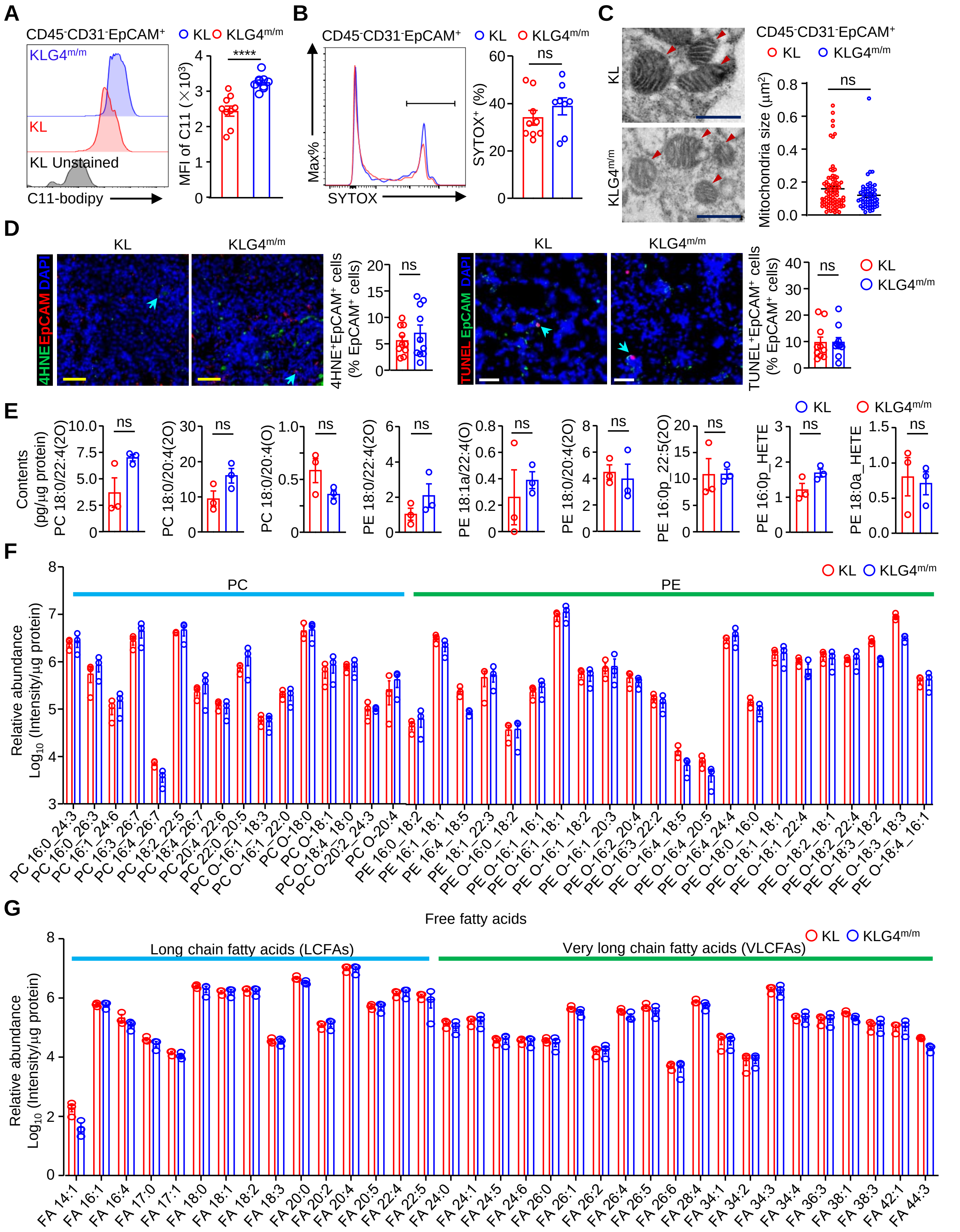

A
CD45-CD31-EpCAM+
KL KLG4m/m
KLG4m/m
KL
KL Unstained
C11-bodipy
MFI of C11 (×10³)
****
B
CD45-CD31-EpCAM+
Max%
SYTOX
SYTOX+ (%)
ns
C
KL
KLG4m/m
Mitochondria size (μm²)
D
4HNE EpCAM DAPI
4HNE+EpCAM+ cells (% EpCAM+ cells)
TUNEL EpCAM DAPI
TUNEL+EpCAM+ cells (% EpCAM+ cells)
E
Contents (pg/μg protein)
PC 18:0/22:4(2O)
PC 18:0/20:4(2O)
PC 18:0/20:4(O)
PE 18:0/22:4(2O)
PE 18:1a/22:4(O)
PE 18:0/20:4(2O)
PE 16:0p_22:5(2O)
PE 16:0p_HETE
PE 18:0a_HETE
F
PC
PE
Relative abundance Log10 (Intensity/μg protein)
PC 16:0_24:3
PC 16:0_26:3
PC 16:1_24:6
PC 16:3_26:7
PC 16:4_26:7
PC 18:2_22:5
PC 18:4_26:7
PC 20:4_22:6
PC 22:0_20:5
PC O-16:1_18:3
PC O-16:1_22:0
PC O-18:0
PC O-18:1
PC O-18:4_18:0
PC O-20:2_24:3
PC O-20:4
PE 16:0_18:2
PE 16:1_18:1
PE 16:4_18:5
PE 18:1_22:3
PE O-16:0_18:2
PE O-16:1_16:1
PE O-16:1_18:1
PE O-16:1_18:2
PE O-16:1_20:3
PE O-16:2_20:4
PE O-16:3_22:2
PE O-16:4_18:5
PE O-16:4_20:5
PE O-16:4_24:4
PE O-18:0_16:0
PE O-18:1_18:1
PE O-18:1_22:4
PE O-18:2_18:1
PE O-18:2_22:4
PE O-18:3_18:2
PE O-18:3_18:3
PE O-18:4_16:1
G
Free fatty acids
Long chain fatty acids (LCFAs)
Very long chain fatty acids (VLCFAs)
Relative abundance Log10 (Intensity/μg protein)
FA 14:1
FA 16:1
FA 16:4
FA 17:0
FA 17:1
FA 18:0
FA 18:1
FA 18:2
FA 18:3
FA 20:0
FA 20:2
FA 20:4
FA 20:5
FA 22:4
FA 22:5
FA 24:0
FA 24:1
FA 24:5
FA 24:6
FA 26:0
FA 26:1
FA 26:2
FA 26:4
FA 26:5
FA 26:6
FA 28:4
FA 34:1
FA 34:2
FA 34:3
FA 34:4
FA 36:3
FA 38:1
FA 38:3
FA 42:1
FA 44:3

Figure S4 Tumor cell-specific knockout of GPX4 in the KL autochthonous mouse model does not result in ferroptosis of tumor cells.

(A) Flow cytometry analysis of C11-bodipy staining of $CD45^{-}CD31^{-}EpCAM^{+}$ tumor cells from the lung tumors of KL (n = 9) and $KLG4^{m/m}$ (n = 9) mice that were intranasally injected with Ad-Cre (2×10^{6} PFU per mice) for 5 weeks followed by intraperitoneal injection of either tamoxifen (Tam, 80 mg/kg, resolved in corn oil) or corn oil every other day for 2 weeks and rest for 3 weeks.

(B) Flow cytometry analysis of SYTOX staining of $CD45^{-}CD31^{-}EpCAM^{+}$ tumor cells from the lung tumors of KL (n = 9) and $KLG4^{m/m}$ (n = 8) mice treated as in (A).

(C) Representative images of transmission electron microscopy analysis (left) and quantification of mitochondrial sizes (right) of $CD45^{-}CD31^{-}EpCAM^{+}$ tumor cells from the lung tumors of KL (n = 85 mitochondria from 15 cells) and $KLG4^{m/m}$ (n = 56 mitochondria from 10 cells) mice treated as in (A). Arrowheads indicated mitochondria.

(D) Representative images and quantitative results of immunofluorescence staining of EpCAM (red) and 4HNE (green) (left panel) or EpCAM (green) and TUNEL (red) (right panel) in lung tumors from KL (n = 10) and $KLG4^{m/m}$ (n = 10) mice treated as in (A). Light blue arrows indicated cells co-stained with DAPI, EpCAM and 4HNE (left) or DAPI, EpCAM and TUNEL (right).

(E) Quantification of the indicated peroxided phospholipid (mainly including PE and PC) in the $CD45^{-}CD31^{-}EpCAM^{+}$ tumor cells of KL (n = 3) and $KLG4^{m/m}$ (n = 3) mice treated as in (A).

(F, G) Relative quantification of PC and PE (F) or free fatty acid (FFA) (G) in $CD45^{-}CD31^{-}EpCAM^{+}$ tumor cells of KL (n = 3) and $KLG4^{m/m}$ (n = 3) mice treated as in (A).

Graphs show mean ± SEM (A-H). Scale bars represent 500 nm (C), 50 μm (D, yellow) or 25 μm (D, white). *** $P < 0.001$, ns: not significant (two-tailed student's *t*-test for A-E and multiple *t*-test for F and G). Data are representative results of two independent experiments (A-D).

Figure S5

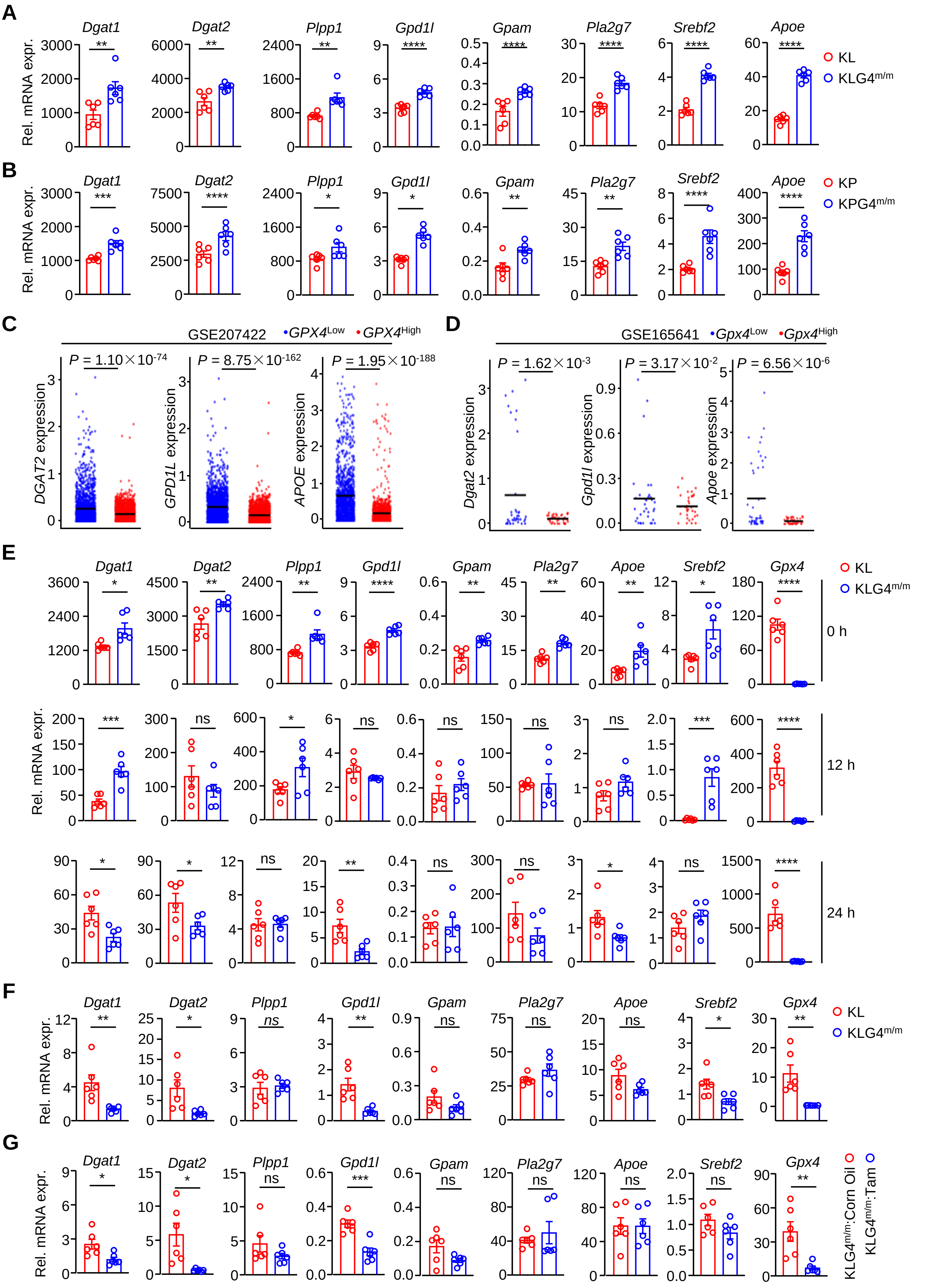
A
Rel. mRNA expr.
Dgat1
Dgat2
Plpp1
Gpd1l
Gpam
Pla2g7
Srebf2
Apoe
KL
KLG4m/m
B
KP
KPG4m/m
C
GSE207422
GPX4Low
GPX4High
P = 1.10×10-74
P = 8.75×10-162
P = 1.95×10-188
DGAT2 expression
GPD1L expression
APOE expression
D
GSE165641
Gpx4Low
Gpx4High
P = 1.62×10-3
P = 3.17×10-2
P = 6.56×10-6
Dgat2 expression
Gpd1l expression
Apoe expression
E
Gpx4
0 h
12 h
24 h
F
G
KLG4m/m:Corn Oil
KLG4m/m:Tam

Figure S5 Expressional patterns of TAG metabolism related genes across different contexts.

(A) Relative mRNA levels of the indicated genes involved in TAG metabolism in $CD45^{-}CD31^{-}EpCAM^{+}$ tumor cells from lung tumors of KL (n = 6) and $KLG4^{m/m}$ mice (n = 6) that were intranasally injected with Ad-Cre (2×10^{6} PFU per mouse) for 5 weeks followed by intraperitoneal injection of tamoxifen every other day for 2 weeks. The mice were rested for another 3 weeks followed by RT-qPCR analysis.

(B) Relative mRNA levels of the indicated genes involved in TAG metabolism in $CD45^{-}CD31^{-}EpCAM^{+}$ tumor cells from lung tumors of KP (n = 6) and $KPG4^{m/m}$ mice (n = 6) that were intranasally injected with Ad-Cre (2×10^{6} PFU per mouse) for 5 weeks followed by intraperitoneal injection of tamoxifen every other day for 2 weeks. The mice were rested for another 3 weeks followed by RT-qPCR analysis.

(C) Scatter plots show the mRNA levels of *DGAT2*, *GPD1L*, and *APOE* between *GPX4*^{Low} (bottom 25%,blue) and *GPX4*^{High} (top 25%, red) tumor cells in the human NSCLC tissues (GEO:207422).

(D) Scatter plots show the mRNA levels of *Dgat2*, *Gpd1l*, and *Apoe* between *Gpx4*^{Low} (bottom 25%,blue) and *Gpx4*^{High} (top 25%, red) tumor cells in the KL NSCLC mouse models (GEO:165641).

(E) Relative mRNA levels of the indicated genes involved in TAG metabolism in $CD45^{-}CD31^{-}EpCAM^{+}$ tumor cells from lung tumors of KL (n = 6) and $KLG4^{m/m}$ (n = 6) mice treated as in (A) that were cultured them *in vitro* for 0, 12, 24 hours followed by RT-qPCR analysis.

(F) Relative mRNA levels of the indicated genes involved in TAG metabolism in subcutaneous syngeneic KL and $KLG4^{m/m}$ tumors. The $CD45^{-}CD31^{-}EpCAM^{+}$ tumor cellss (5×10^{6}/mouse) sorted from lung tumors of Ad-Cre-infected tamoxifen-treated KL or $KLG4^{m/m}$ mice treated as in (A) were subcutaneously inoculated into the flanks of wild-type C57BL/6 mice (n = 6 for each group) for 50 days followed by RT-qPCR analysis.

(G) Relative mRNA levels of the indicated genes involved in TAG metabolism in Tam-treated subcutaneous syngeneic KL and $KLG4^{m/m}$ tumors. The $CD45^{-}CD31^{-}EpCAM^{+}$ tumor cells (5×10^{6}/mouse) sorted from Ad-Cre-infected $KLG4^{m/m}$ mice were subcutaneously inoculated into the flanks of wild-type C57BL/6 mice for 3 weeks (when the tumors were palpable) followed by one-weak tamoxifen treatment (80 mg/kg body weight daily) and RT-qPCR analysis (n = 6 each group).

Graphs show mean ± SEM (A-G). * $P < 0.05$, ** $P < 0.01$, *** $P < 0.001$, **** $P <0.0001$, ns: not significant (two-tailed student's *t*-test). Data are representative results of two independent experiments (A-B and E-G).

Figure S6

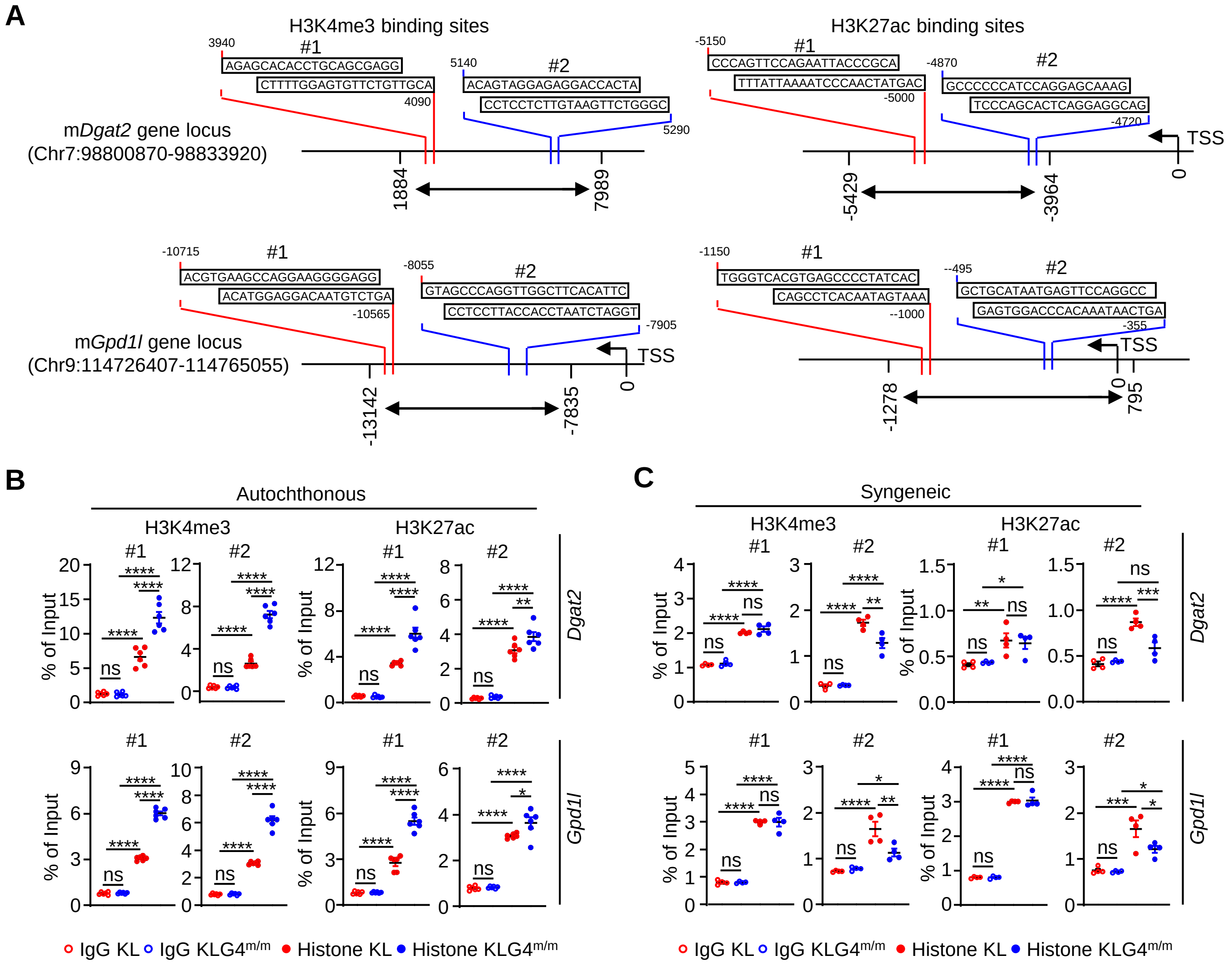

Figure S6 H3K4me3 and H3K27ac modifications on mouse *Dgat2* loci.

(A) A scheme of mouse *Dgat2* loci (top) and *Gpd1l* loci (bottom) that contained H3K4me3 (left) and H3K27ac (right) modifications.

(B) Chromatin immunoprecipitation qPCR (ChIP-qPCR) assay of H3K4me3 and H3K27ac modifications on the *Dgat2* loci (top) and *Gpd1l* loci (bottom) in CD45$^-$CD31$^-$EpCAM$^+$ tumor cells from lung tumors of Ad-Cre-infected Tam-treated KL (n = 6) and KLG4$^{m/m}$ (n = 6) mice.

(C) ChIP-qPCR assay of H3K4me3 and H3K27ac modifications on the *Dgat2* loci (top) and *Gpd1l* loci (bottom) in Tam-treated subcutaneous syngeneic KL (n = 4) and KLG4$^{m/m}$ (n = 4) tumors. The CD45$^-$CD31$^-$EpCAM$^+$ tumor cells from lung tumors of Ad-Cre-infected KL and KLG4$^{m/m}$ mice were subcutaneously inoculated into the flanks of wild-type C57BL/6 mice for 3 weeks (when the tumors were palpable) followed by one-weak tamoxifen treatment (80 mg/kg body weight daily) and ChIP-qPCR analysis. Graphs show mean ± SEM. * $P < 0.05$, ** $P < 0.01$, **** $P < 0.0001$, ns: not significant (one-way ANOVA). Data are representative results of two independent experiments.

Figure S7

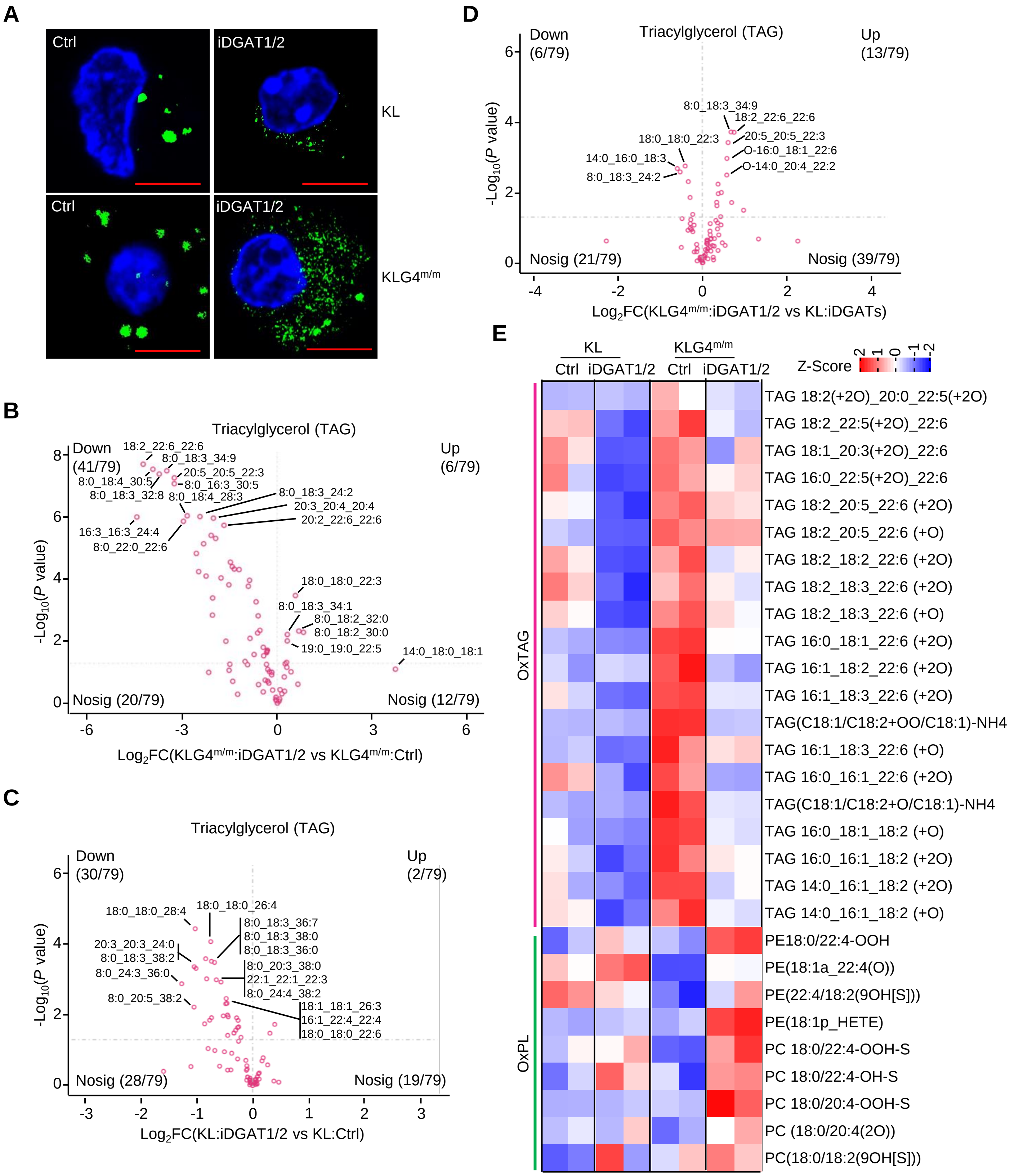

A
Ctrl
iDGAT1/2
KL
KLG4m/m
B
Triacylglycerol (TAG)
Down (41/79)
Up (6/79)
Nosig (20/79)
Nosig (12/79)
-Log10(P value)
Log2FC(KLG4m/m:iDGAT1/2 vs KLG4m/m:Ctrl)
C
Triacylglycerol (TAG)
Down (30/79)
Up (2/79)
Nosig (28/79)
Nosig (19/79)
-Log10(P value)
Log2FC(KL:iDGAT1/2 vs KL:Ctrl)
D
Triacylglycerol (TAG)
Down (6/79)
Up (13/79)
Nosig (21/79)
Nosig (39/79)
-Log10(P value)
Log2FC(KLG4m/m:iDGAT1/2 vs KL:iDGATs)
E
KL
KLG4m/m
Ctrl
iDGAT1/2
Z-Score
OxTAG
OxPL
TAG 18:2(+2O)_20:0_22:5(+2O)
TAG 18:2_22:5(+2O)_22:6
TAG 18:1_20:3(+2O)_22:6
TAG 16:0_22:5(+2O)_22:6
TAG 18:2_20:5_22:6 (+2O)
TAG 18:2_20:5_22:6 (+O)
TAG 18:2_18:2_22:6 (+2O)
TAG 18:2_18:3_22:6 (+2O)
TAG 18:2_18:3_22:6 (+O)
TAG 16:0_18:1_22:6 (+2O)
TAG 16:1_18:2_22:6 (+2O)
TAG 16:1_18:3_22:6 (+2O)
TAG(C18:1/C18:2+OO/C18:1)-NH4
TAG 16:1_18:3_22:6 (+O)
TAG 16:0_16:1_22:6 (+2O)
TAG(C18:1/C18:2+O/C18:1)-NH4
TAG 16:0_18:1_18:2 (+O)
TAG 16:0_16:1_18:2 (+2O)
TAG 14:0_16:1_18:2 (+2O)
TAG 14:0_16:1_18:2 (+O)
PE18:0/22:4-OOH
PE(18:1a_22:4(O))
PE(22:4/18:2(9OH[S]))
PE(18:1p_HETE)
PC 18:0/22:4-OOH-S
PC 18:0/22:4-OH-S
PC 18:0/20:4-OOH-S
PC (18:0/20:4(2O))
PC(18:0/18:2(9OH[S]))

Figure S7 Inhibition of DGAT1/2 reprograms PUFA-PE/PC and oxPE/PC accumulation in KL and KLG4$^{m/m}$ tumor cells.

(A) Representative images of $CD45^{-}CD31^{-}EpCAM^{+}$ tumor cells from lung tumors of KL and KLG4$^{m/m}$ mice with or without iDGAT1/2 treatment. The KL and KLG4$^{m/m}$ mice that were intranasally injected with Ad-Cre (2×10^{6} PFU per mouse) for 5 weeks followed by intraperitoneal injection of tamoxifen every other day for 2 weeks. One week after Tam treatment, the mice were injected with iDGAT1/2 (composing of T863 and PF06424439, 20 mg and 40 mg per kg body weight, respectively) every other day by gavage for 5 weeks. The mice were rest for one week followed by subsequent analyses.

(B) Volcano plot showing the levels of triacylglycerols (TAGs) in $CD45^{-}CD31^{-}EpCAM^{+}$ tumor cells derived from iDGAT1/2 treated KLG4$^{m/m}$ mice (n = 2) versus Control treated KLG4$^{m/m}$ mice (n = 2) treated as in (A) under indicated treatment

(C) Volcano plot showing the levels of triacylglycerols (TAGs) in $CD45^{-}CD31^{-}EpCAM^{+}$ tumor cells derived from iDGAT1/2 treated KL mice (n = 2) versus Control treated KL mice (n = 2) treated as in (A) under indicated treatment.

(D) Volcano plot showing the levels of triacylglycerols (TAGs) in $CD45^{-}CD31^{-}EpCAM^{+}$ tumor cells derived from iDGAT1/2 treated KLG4$^{m/m}$ mice (n = 2) versus iDGAT1/2 treated KL mice (n = 2) treated as in (A) under indicated treatment.

(E) Heat map showing the changes of classical PUFA-oxTAG and ferroptosis-related oxidized PC and PE in tumor cells from KL and KLG4$^{m/m}$ mice with or without iDGAT1/2 treatment. Rows represent Z-score normalized intensities, columns represent samples, color-coded from red (high intensity) to blue (low intensity).

Figure S8

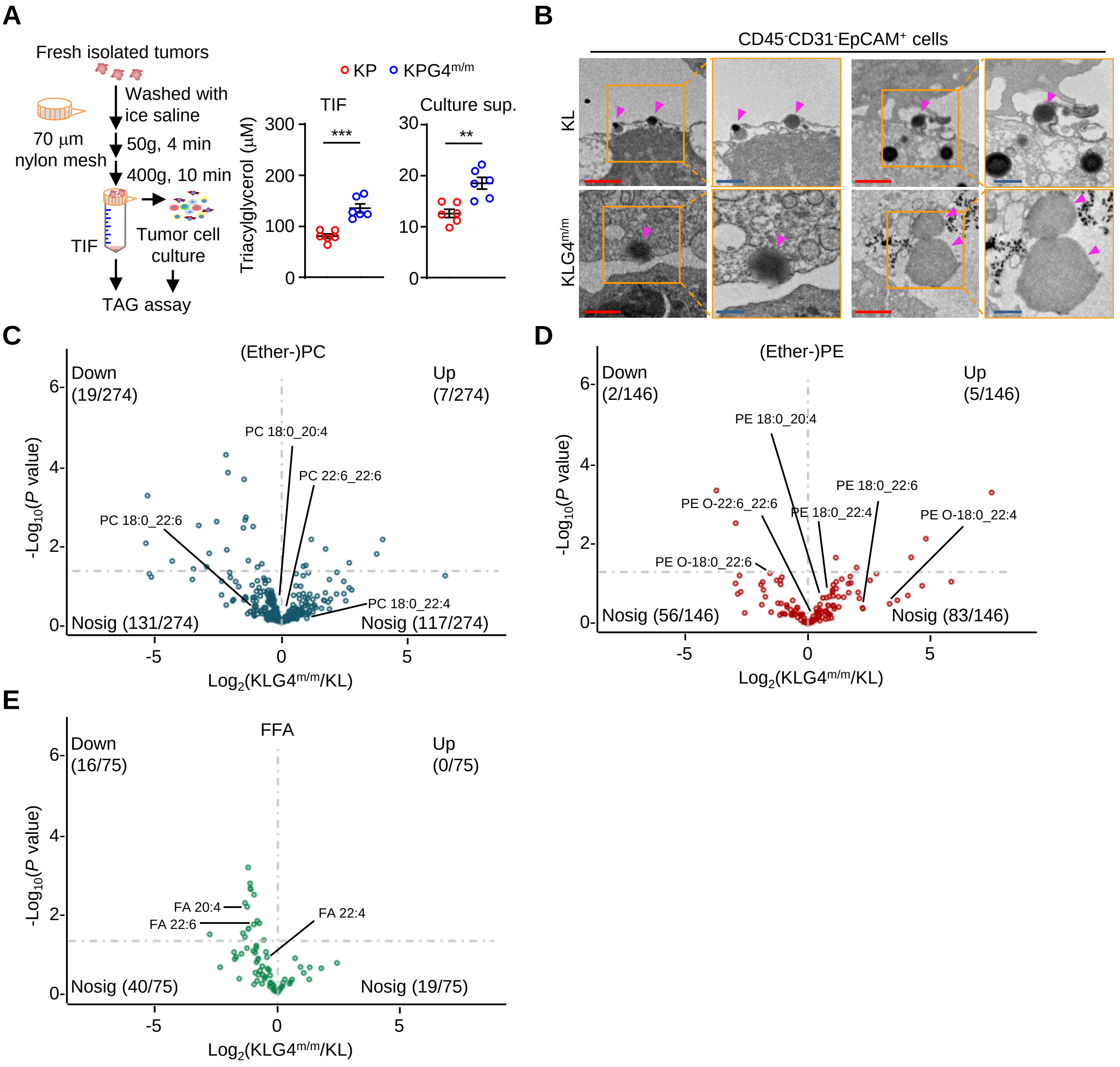

Figure S8 Quantification of the lipids released from tumor cells.

(A) A scheme of tumor interstitial fluid (TIF) and tumor cells (CD45-CD31-EpCAM+) cultured supernatants collection (left) and quantification of the TAG (right) in TIF and tumor cells cultured supernatants from lung tumors of KP (n = 6) and KPG4$^{m/m}$ (n = 6) mice that were intranasally injected with Ad-Cre (2×10^6 PFU per mouse) for 5 weeks followed by intraperitoneal injection of tamoxifen every other day for 2 weeks. The mice were allowed to rest for another 3 weeks followed by TAG assessment.

(B) Representative images of transmission electron microscopy of CD45-CD31-EpCAM+ tumor cells from the lung tumors of KL (n = 4) and KLG4$^{m/m}$ (n = 4) mice that were intranasally injected with Ad-Cre (2×10^6 PFU per mice) for 5 weeks followed by intraperitoneal injection of either tamoxifen (Tam, 80 mg/kg, resolved in corn oil) or corn oil every other day for 2 weeks and rest for 3 weeks. The orange boxed areas were shown at a higher magnification on the right.

(C-E) Lipidomic profile of phosphatidylcholine (PC) and ether-linked PC (ether-PC) (B), phosphatidylethanolamine (PE) and ether-linked PE (ether-PE) (C), and free fatty acid (FFA) (D) in the cultured supernatants of CD45-CD31-EpCAM+ tumor cells from lung tumors of KL (n = 5) and KLG4$^{m/m}$ (n = 5) mice treated as in (B).

Scale bars represent 1 µm (red, B) and 500 nm (blue, B). Data are representative results of two independent experiments (A-B).

Figure S9

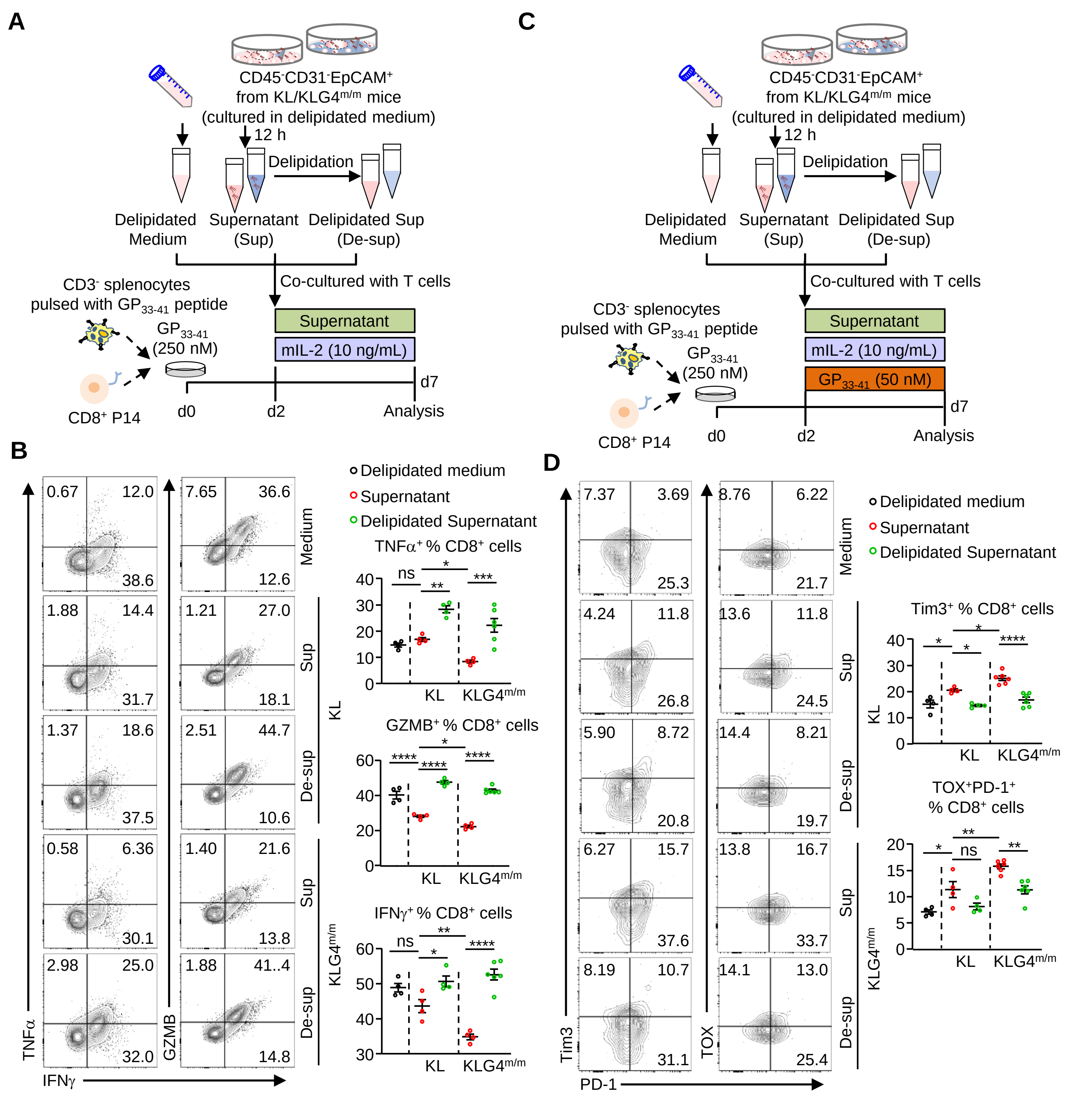

Figure S9 KPG4$^{m/m}$ tumor cell-secreted lipid mediators promote dysfunction and exhaustion of CD8$^+$ T cells.

(A-B) A scheme of acute *in vitro* activation (A) and representative flow charts (B, left) and quantitative analysis (B, right) of P14 cells (n = 4 or 6 for each group) in the presence of the supernatants or the delipidated supernatants from KL or KLG4$^{m/m}$ CD45$^-$CD31$^-$EpCAM$^+$ tumor cells.

(C-D) A scheme of chronic *in vitro* activation (C) and representative flow charts (D, left) and quantitative analysis (D, right) of P14 cells (n = 4 or 6 for each group)in the presence of the supernatants or the delipidated supernatants from KL or KLG4$^{m/m}$ CD45$^-$CD31$^-$EpCAM$^+$ tumor cells.

Graphs show mean ± SEM (B and D). * $P < 0.05$, ** $P < 0.01$, *** $P < 0.001$, **** $P < 0.0001$. ns: not significant (two-way ANOVA for B and D). Data are representative results of two independent experiments (B and D).

Figure S10

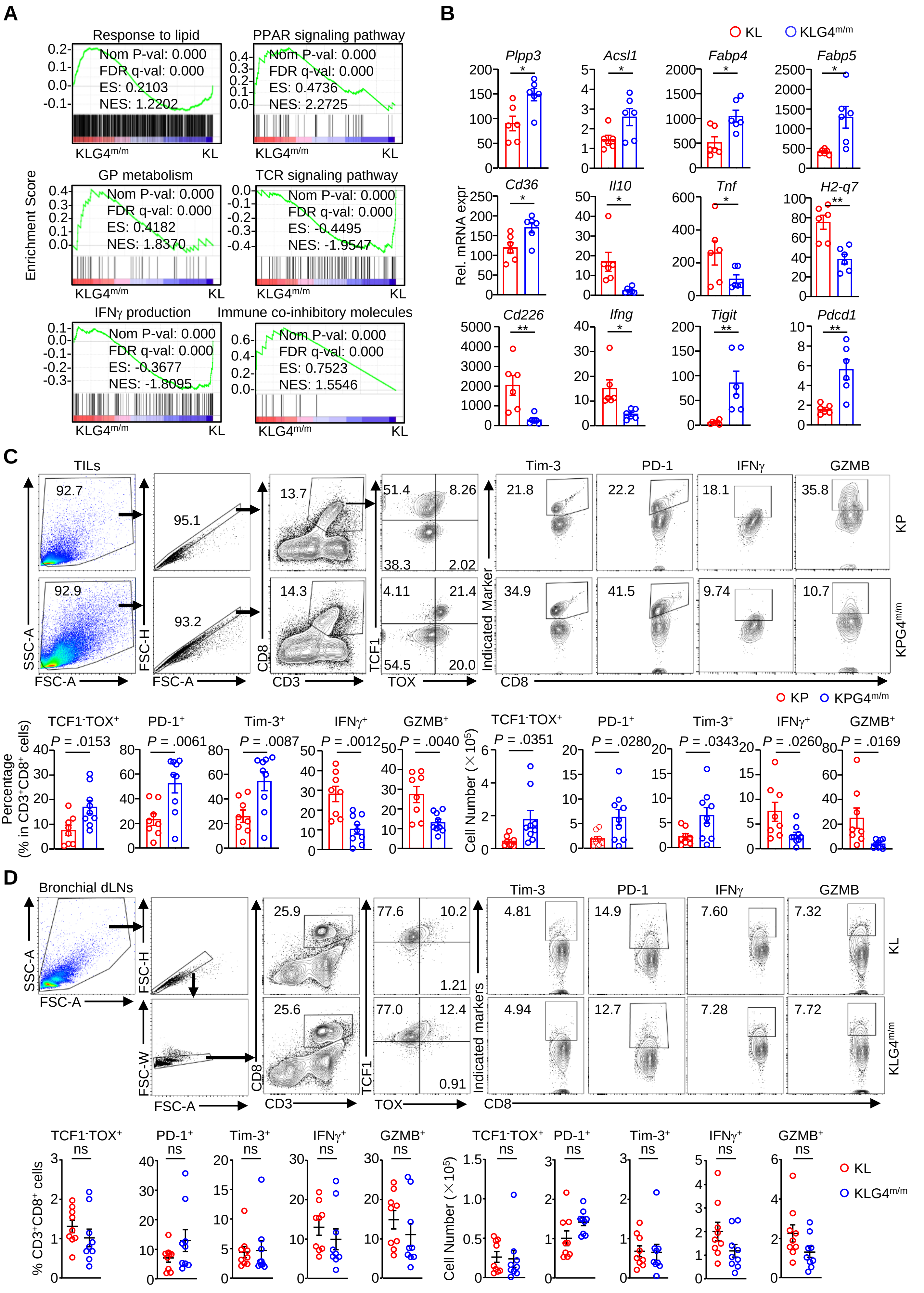

A
Response to lipid
Nom P-val: 0.000
FDR q-val: 0.000
ES: 0.2103
NES: 1.2202
PPAR signaling pathway
Nom P-val: 0.000
FDR q-val: 0.000
ES: 0.4736
NES: 2.2725
GP metabolism
Nom P-val: 0.000
FDR q-val: 0.000
ES: 0.4182
NES: 1.8370
TCR signaling pathway
Nom P-val: 0.000
FDR q-val: 0.000
ES: -0.4495
NES: -1.9547
IFNγ production
Nom P-val: 0.000
FDR q-val: 0.000
ES: -0.3677
NES: -1.8095
Immune co-inhibitory molecules
Nom P-val: 0.000
FDR q-val: 0.000
ES: 0.7523
NES: 1.5546
Enrichment Score
KLG4m/m
KL
B
KL
KLG4m/m
Rel. mRNA expr
Plpp3
Acsl1
Fabp4
Fabp5
Cd36
Il10
Tnf
H2-q7
Cd226
Ifng
Tigit
Pdcd1
C
TILs
92.7
95.1
13.7
51.4
8.26
38.3
2.02
92.9
93.2
14.3
4.11
21.4
54.5
20.0
Tim-3
PD-1
IFNγ
GZMB
21.8
22.2
18.1
35.8
34.9
41.5
9.74
10.7
KP
KPG4m/m
SSC-A
FSC-A
FSC-H
CD8
CD3
TCF1
TOX
Indicated Marker
CD8
Percentage (% in CD3+CD8+ cells)
TCF1-TOX+
P = .0153
PD-1+
P = .0061
Tim-3+
P = .0087
IFNγ+
P = .0012
GZMB+
P = .0040
Cell Number (×10^5)
TCF1-TOX+
P = .0351
PD-1+
P = .0280
Tim-3+
P = .0343
IFNγ+
P = .0260
GZMB+
P = .0169
D
Bronchial dLNs
SSC-A
FSC-A
FSC-H
FSC-W
25.9
77.6
10.2
1.21
25.6
77.0
12.4
0.91
Tim-3
PD-1
IFNγ
GZMB
4.81
14.9
7.60
7.32
4.94
12.7
7.28
7.72
KL
KLG4m/m
CD8
CD3
TCF1
TOX
Indicated markers
% CD3+CD8+ cells
Cell Number (×10^5)
TCF1-TOX+
PD-1+
Tim-3+
IFNγ+
GZMB+
ns

Figure S10 KPG4$^{m/m}$ tumor cell-secreted lipid mediators promote dysfunction and exhaustion of CD8$^+$ T cells.

(A) GSEA analysis of the response to lipid, PPAR signaling pathway, Glyerophospholipid metabolism, TCR signaling pathway, IFNγ production and immune co-inhibitory molecules based on the transcriptome data of CD8$^+$ TILs from lung tumors of KLG4$^{m/m}$ (n = 2) and KL (n = 2) mice that were intranasally injected with Ad-Cre (2×10^6 PFU per mice) for 5 weeks followed by intraperitoneal injection of either tamoxifen (Tam, 80 mg/kg, resolved in corn oil) or corn oil every other day for 2 weeks and rest for 3 weeks.

(B) RT-qPCR analysis of the signature genes involved in lipid metabolism and T cells response in CD45$^-$CD31$^-$EpCAM$^+$ tumor cells from lung tumors of KL (n = 6) and KLG4$^{m/m}$ (n = 6) mice treated as in (A).

(C) Representative flow cytometry images (upper charts) and quantification analysis (lower graphs) of tumor-infiltrated lymphocytes (TILs) from lung tumors of KP (n = 8) and KPG4$^{m/m}$ (n = 9) mice that were intranasally injected with Ad-Cre (2×10^6 PFU per mouse) for 5 weeks followed by intraperitoneal injection of tamoxifen (Tam, 80 mg/kg, resolved in corn oil) every other day for 2 weeks and rest for 3 weeks.

(D) Representative flow cytometry images (upper charts) and quantification analysis (lower graphs) of bronchial draining lymph nodes (dLNs) from lung tumors of KL (n = 9) and KLG4$^{m/m}$ (n = 9) mice that were intranasally injected with Ad-Cre (2×10^6 PFU per mouse) for 5 weeks followed by intraperitoneal injection of tamoxifen (Tam, 80 mg/kg, resolved in corn oil) every other day for 2 weeks and rest for 3 weeks.

Graphs show mean ± SEM (B, lower graphs of C and D). * $P < 0.05$, ** $P < 0.01$, *** $P < 0.001$, **** $P < 0.0001$. ns: not significant (two-tailed student's *t*-test for B and lower graphs of C and D). Data are representative results of two independent experiments (B and lower graphs of C and D).

Figure S11

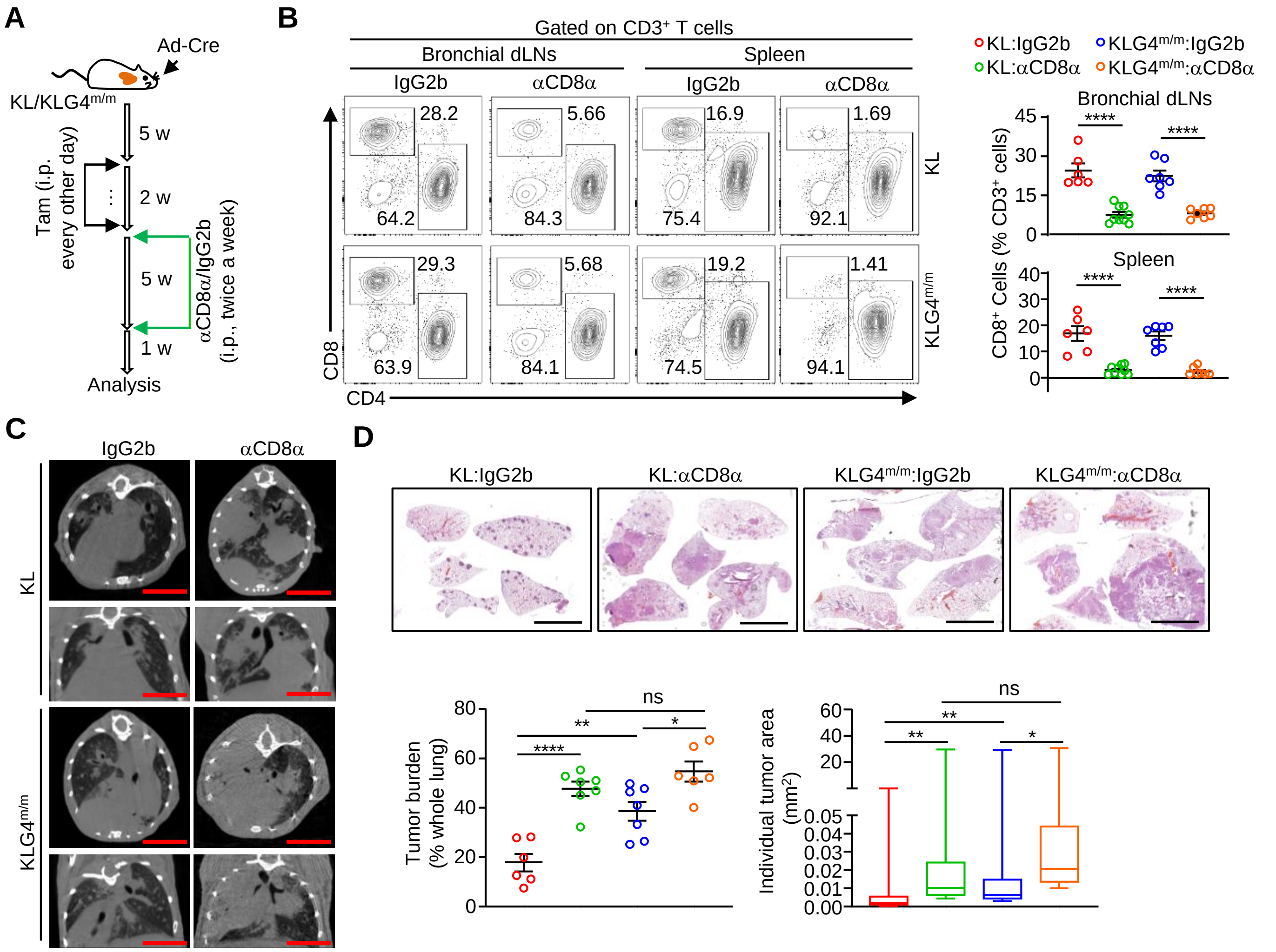

Figure S11 Depletion of CD8⁺ T cells accelerates tumor progression of tumor-bearing KL/KLG4$^{m/m}$ mice.

(A) Schematic illustration depicts tumor induction and CD8⁺ T cells depletion (anti-mouse CD8α, αCD8α, Clone:2.43) in KL and KLG4$^{m/m}$ mice that were intranasally injected with Ad-Cre (2×10^{6} PFU per mouse) for 5 weeks followed by intraperitoneal injection of tamoxifen every other day for 2 weeks. After completion of tamoxifen treatment, the mice were intraperitoneally injected with αCD8α or IgG2b (200 µg per injection, respectively) twice a week for 5 weeks. The mice were rest for one week followed by various analyses.

(B) Representative flow cytometry images (left charts) and quantification analysis (right graphs) of the percentage of CD8⁺ T cells in bronchial draining lymph nodes (top-right of B) or spleen (bottom-right of B) from the tumor-bearing KL (n=9 for αCD8α and n=6 for IgG2b, respectively) and KLG4$^{m/m}$ (n=7 for αCD8α and n=7 for IgG2b, respectively) mice treated as described in (A).

(C) Representative images of micro-CT of tumor-burdened lungs from the KL (n=7 for αCD8α and n=6 for IgG2b, respectively) and KLG4$^{m/m}$ (n=6 for αCD8α and n=7 for IgG2b, respectively) mice treated as described in (A).

(D) Representative images of HE staining (top) and statistics of tumor burdens and individual tumor sizes (bottom) of tumor-burdened lungs from the KL (n=7 for αCD8α and n=6 for IgG2b, respectively) and KLG4$^{m/m}$ (n=6 for αCD8α and n=7 for IgG2b, respectively) mice treated as described in (A).

Graphs show mean ± SEM (B, D). * $P < 0.05$, ** $P < 0.01$, *** $P < 0.001$, **** $P < 0.0001$. ns: not significant (one-way ANOVA for B and D). Scale bars represent 5 mm (B and D). Data are representative results of two independent experiments (B-D).

Figure S12

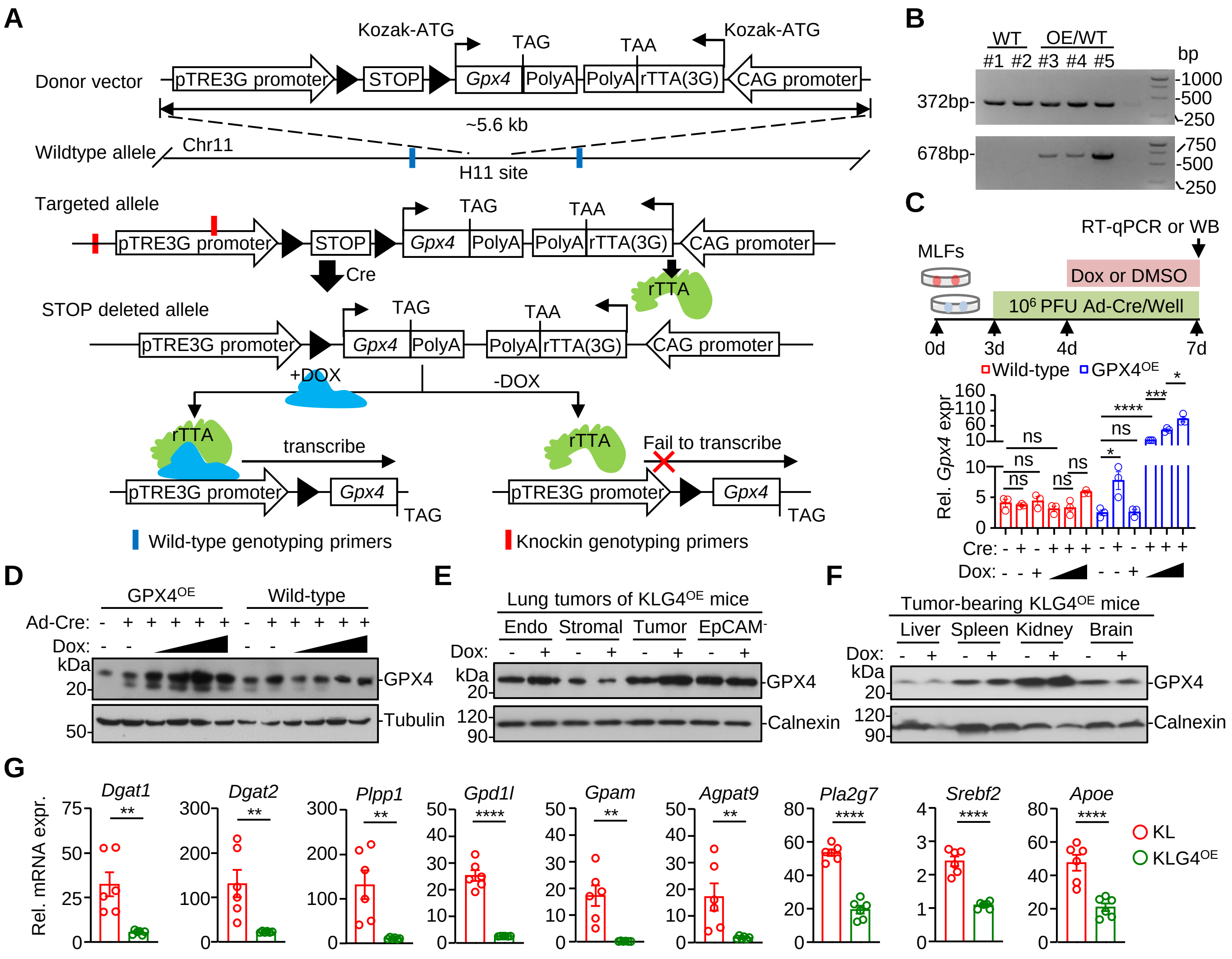

Figure S12 Generation and analysis of tumor cell-specific *Gpx4* overexpression mouse strain.

(A) A scheme of the strategy for the generation of H11-pTRE3G-LSL-m*Gpx4*- CAG-rTTA (*Gpx4*^{OE}) mice.

(B) PCR analysis of the tail genomic DNAs from wild-type (Lane #1, #2) or *Gpx4*^{OE} (Lane #3-5) mice.

(C, D) Experimental design (C, upper scheme), RT-qPCR (C, lower graph) (n = 3 biological replicates) or immunoblot analysis (D) of GPX4 in MLFs lysis from wild-type and *Gpx4*$^{m/m}$ MLFs infected with Ad-Cre for 24 hours followed by treatment with Doxycycline (Dox, 20, 50, and 100 µg/mL) for 72 hours.

(E, F) Immunoblot analysis of GPX4 in FACS-sorted endothelial cells, stromal cells, tumor cells or EpCAM$^-$ cells in the tumors (E) or in liver, spleen, kidney or brain from KLG4^{OE} mice that were infected with Ad-Cre (2×10^6 PFU per mouse) for 5 weeks followed by feeding of normal (-Dox) or Dox-supplemented (+Dox) chow food for 8 weeks.

(G) RT-qPCR analysis of the signature genes involved in TAG synthesis in CD45$^-$CD31$^-$EpCAM$^+$ tumor cells from lung tumors of KL (n = 6) and KLG4^{OE} (n = 6) mice treated as in (E).

Graphs show mean ± SEM (C and G). * $P < 0.05$, ** $P < 0.01$, *** $P < 0.001$, **** $P < 0.0001$. ns: not significant (one-way ANOVA for C and two-tailed student's *t*-test for G). Data are representative results of two independent experiments (B-G).

Figure S13

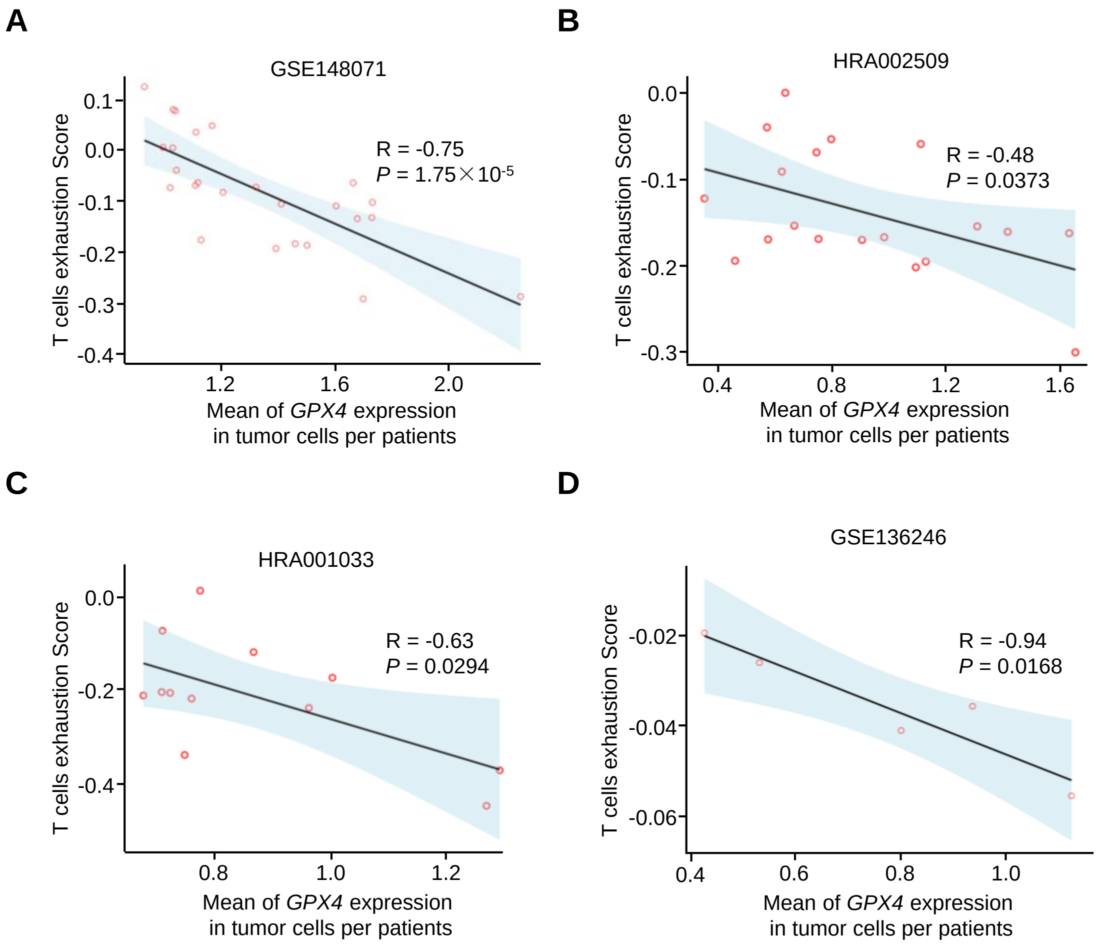

Figure S13 *GPX4* expression of tumor cells negatively correlates with T cells exhaustion score of T cells in NSCLC patients.

(A-D) Correlation analysis between the mean *GPX4* expression in tumor cells and the exhaustion score of T cells across patients in the GSE148071 (A), HRA002509 (B), HRA001033 (C), and GSE136246 (D) datasets. Each dot represents one patient. The x-axis indicates the mean *GPX4* expression level in tumor cells from single-cell RNA sequencing data, while the y-axis represents the T cell exhaustion score of T cells from the same patient. R: Pearson correlation coefficient. *P* value were calculated using a two-tailed test based on the *t*-distribution.

Figure S14

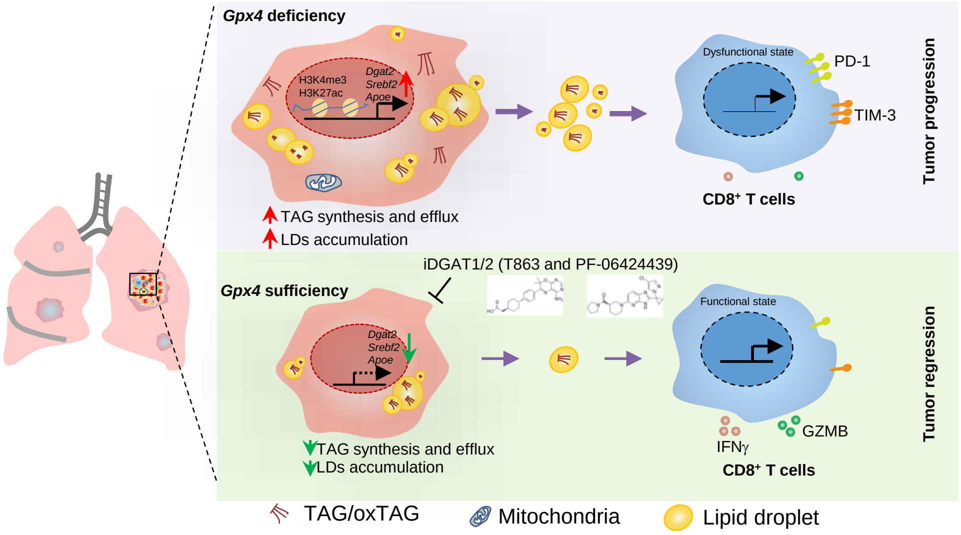

Figure S14 A model of metabolic crosstalk between *Gpx4*-deficient tumor cells and immune cells in the TME of autochthonous NSCLC.

Inducible tumor cell-specific knockout of *Gpx4* reprograms the TAG metabolism and efflux to evade ferroptosis in tumor cells and induce dysfunction and exhaustion of $CD8^+$ T in the TME was compromised and the progression of NSCLC is aggravated. Inhibition of TAG synthesis re-sensitizes tumor cells to ferroptosis and inhibits the progression of NSCLC. See the Discussion for a detailed description. TAG: Triacylglycerol; MHCI: major histocompatibility complex I; PLs: phospholipids; LDs: lipid droplets; *Dgat2*: Diacylglycerol acyltransferase 2; *Srebf2*: Sterol regulatory element binding transcription factor 2; *Apoe*: Apolipoprotein E.
